# Predictors of mortality in patients with coronavirus disease 2019: a systematic review and meta-analysis

**DOI:** 10.1186/s12879-021-06369-0

**Published:** 2021-07-08

**Authors:** Changcheng Shi, Limin Wang, Jian Ye, Zhichun Gu, Shuying Wang, Junbo Xia, Yaping Xie, Qingyu Li, Renjie Xu, Nengming Lin

**Affiliations:** 1 Department of Clinical Pharmacy, Key Laboratory of Clinical Cancer Pharmacology and Toxicology Research of Zhejiang Province, Affiliated Hangzhou First People’s Hospital, Zhejiang University School of Medicine, Hangzhou, China; 2 Department of Respiratory Medicine, Affiliated Hangzhou First People’s Hospital, Zhejiang University School of Medicine, Hangzhou, China; 3 Department of Pharmacy, Renji Hospital, School of Medicine, Shanghai Jiaotong University, Shanghai, China; 4 Department of Nosocomial Infection Control, Affiliated Hangzhou First People’s Hospital, Zhejiang University School of Medicine, Hangzhou, China; 5 Department of Hematology, Affiliated Hangzhou First People’s Hospital, Zhejiang University School of Medicine, Hangzhou, China; 6 Department of Clinical Pharmacy, Shaoxing Women and Children’s Hospital, Shaoxing, China; 7 Department of Clinical Pharmacology, Translational Medicine Research Center, Key Laboratory of Clinical Cancer Pharmacology and Toxicology Research of Zhejiang Province, Affiliated Hangzhou First People’s Hospital, Zhejiang University School of Medicine, No.261 Huansha Road, Hangzhou, China

**Keywords:** COVID-19, Mortality, Predictors, Systematic review, Meta-analysis

## Abstract

**Background:**

Coronavirus disease 2019 (COVID-19) is associated with a high mortality rate, especially in patients with severe illness. We conducted a systematic review and meta-analysis to assess the potential predictors of mortality in patients with COVID-19.

**Methods:**

PubMed, EMBASE, the Cochrane Library, and three electronic Chinese databases were searched from December 1, 2019 to April 29, 2020. Eligible studies reporting potential predictors of mortality in patients with COVID-19 were identified. Unadjusted prognostic effect estimates were pooled using the random-effects model if data from at least two studies were available. Adjusted prognostic effect estimates were presented by qualitative analysis.

**Results:**

Thirty-six observational studies were identified, of which 27 were included in the meta-analysis. A total of 106 potential risk factors were tested, and the following important predictors were associated with mortality: advanced age, male sex, current smoking status, preexisting comorbidities (especially chronic kidney, respiratory, and cardio-cerebrovascular diseases), symptoms of dyspnea, complications during hospitalization, corticosteroid therapy and a severe condition. Additionally, a series of abnormal laboratory biomarkers of hematologic parameters, hepatorenal function, inflammation, coagulation, and cardiovascular injury were also associated with fatal outcome.

**Conclusion:**

We identified predictors of mortality in patients with COVID-19. These findings could help healthcare providers take appropriate measures and improve clinical outcomes in such patients.

**Supplementary Information:**

The online version contains supplementary material available at 10.1186/s12879-021-06369-0.

## Background

Coronavirus disease 2019 (COVID-19) is a severe emerging infection caused by a novel coronavirus (severe acute respiratory syndrome coronavirus 2, SARS-CoV-2). In December 2019, the first case of COVID-19 infection was reported in Wuhan, China [1]. Since then, the disease has spread rapidly around the world within a short period of time. As of May 9, 2020, there were more than 3.85 million laboratory-confirmed cases of COVID-19 and 265 thousand deaths, with a case fatality rate of 6.9% [2].

To help better understand factors associated with an increased risk of mortality, increasing prognostic studies have been published [3–38]. However, most of the studies included relatively small sample sizes and presented inconsistent findings. To obtain an adequate number of cases for precision estimation of the correlations between predictors and a fatal outcome, we conducted a systematic review and meta-analysis to identify and summarize predictors of mortality in patients with COVID-19 infection.

## Methods

This study was conducted in accordance with the guidelines for systematic review and meta-analysis of prognostic factors [39] and reported according to the Preferred Reporting Items for Systematic Reviews and Meta-Analyses (PRISMA) statement [40].

### Literature search

PubMed, EMBASE, the Cochrane Library, and three electronic Chinese databases (Chinese National Knowledge Infrastructure, Wanfang, and VIP databases) were searched from December 1, 2019 to April 29, 2020. For those not familiar with Chinese electronic databases, details of these databases are presented in Additional file 1. The search terms were “(coronavirus OR SARS-CoV-2 OR COVID-19 OR 2019-nCoV) AND (mortality OR survivor OR death OR fatality OR deceased)”. To identify additional eligible studies, reference lists of included studies and relevant review articles were scanned. No language restriction was imposed.

### Inclusion and exclusion criteria

Two reviewers independently reviewed and selected studies for inclusion. Randomized controlled trials and observational studies reporting potential predictors of mortality in patients with COVID-19 infection were included in the meta-analysis. The exclusion criteria were as follows: (1) reviews, case reports, editorials, and conference abstracts, (2) studies for which mortality data were not available, (3) studies with small sample sizes (≤ 30 participants or ≤ 10 nonsurvivors), and (4) studies that included only pregnant women or children.

### Data extraction and quality assessment

The following data were extracted from each study by two authors independently: author, design, setting, patient recruitment period, location, sample size, patient characteristics, severity of illness, mortality rate, and potential risk factors. Any disagreement was resolved by consensus. The quality of the included studies was assessed using the Quality in Prognostic Factor Studies (QUIPS) tool. This tool is composed of six items: study participation, study attrition, prognostic factor measurement, outcome measurement, study confounding, and statistical analysis and reporting [41].

### Statistical analysis

The pooled unadjusted estimates for each predictor were performed if data from at least two studies were available. The results as risk ratios (RRs) for dichotomous data and weighted mean differences (WMDs) for continuous outcomes were presented, both with 95% confidence intervals (CIs). For studies that presented data as medians and interquartile ranges, we calculated the means and standard deviations based on the formulas used by Wan et al. [42]. To provide conservative pooling estimates, we applied a random-effects model in all analyses. Heterogeneity was assessed using the I^2^ statistic, and a value of > 50% was considered significant heterogeneity [43]. In the presence of significant heterogeneity, sensitivity analyses were performed to assess the stability of the results by omitting the largest (or smallest) study. We also calculated the fixed-effects model for additional sensitivity analyses. Publication bias was examined by Egger’s test. All statistical analyses were performed using RevMan version 5.3 (the Cochrane Collaboration) and Stata 15.0 software (StataCorp, College Station, Texas, USA). Pooled adjusted estimates for predictors were not available because only few original studies reported adjusted data, different types of effect measures were used (such as odds ratios and hazard ratios), and potential overlap of the patients existed between the included studies. Therefore, we presented adjusted data using qualitative analysis.

## Results

### Literature search

**Fig. 1:**
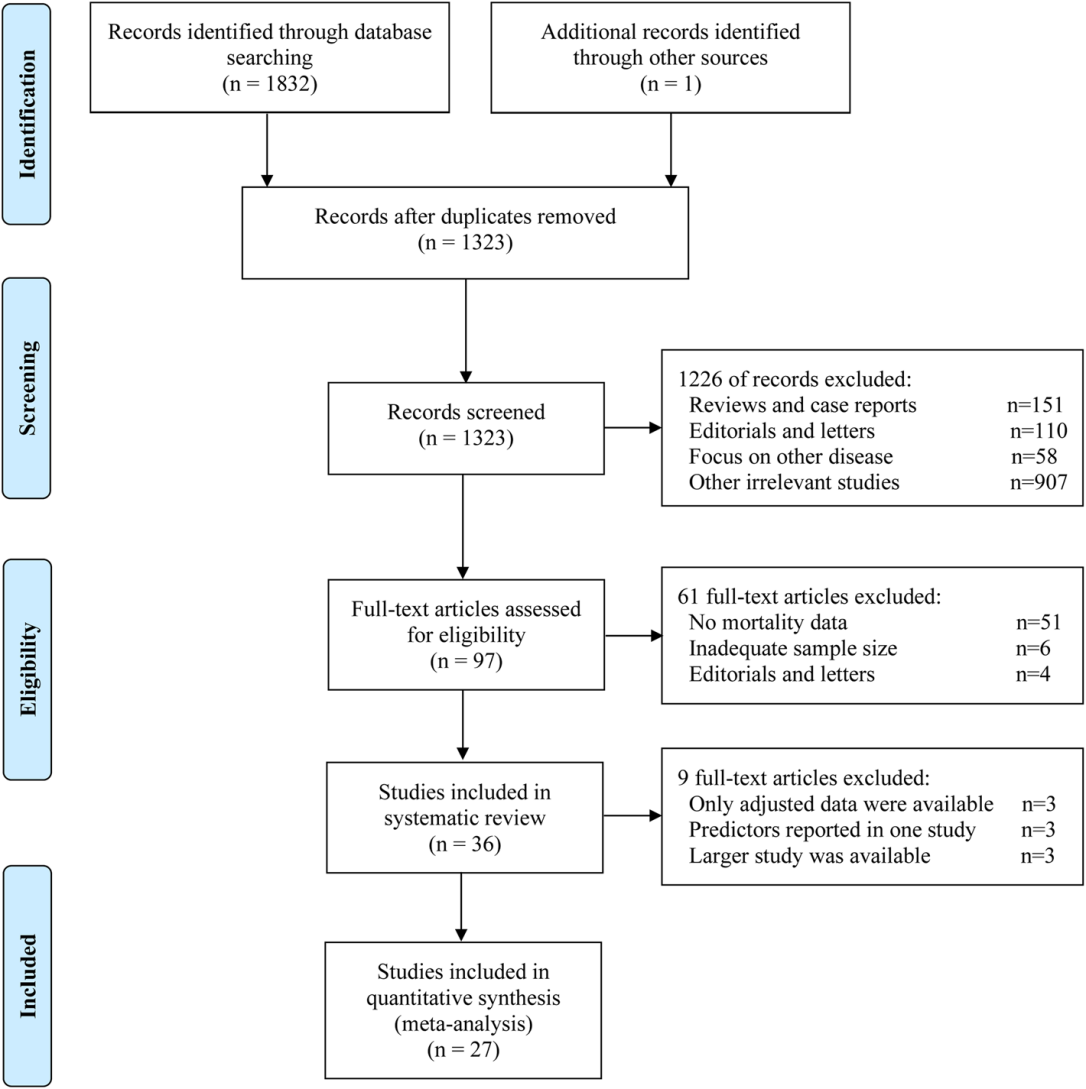
Flow diagram of the literature search and selection process

The search process identified 1832 publications in the initial search. After screening, we included 36 studies in the systematic review [3–38], of which 27 were included in the meta-analysis [3, 5, 6, 9, 11–13, 15, 16, 19–26, 28–36, 38]. The literature selection process is shown in Fig. 1. We noted that several included studies were from the same hospital and had overlapping patient recruitment periods. To avoid potential patient overlap, we extracted only the data from the studies with the largest sample size for each evaluated predictor if multidata were available from the same hospital.

The characteristics of the studies included in the systematic review are presented in Table 1. The sample size in each study ranged from 52 to 5688, and the mortality rate ranged from 3.1 to 61.5%. All the studies were from China with the exception of two studies conducted in the USA [22, 24] and one study conducted in Italy [11]. The mean ages of the patients ranged from 47 to 70 years, and the proportion of female patients ranged from 18.0 to 60.2%. The quality assessment of the included studies is presented in Table S1. The item showing greatest potential risk of bias was study confounding. The quality of some studies was also sub-optimal with respect to the remaining five items.

**Table 1:** Characteristics of the studies included in the systematic review

Study	Country	Design	Setting	Recruitment periods ^a^	Severity of illness (%)	Sample size	Female (%)	Age (years) ^c^	Mortality (%)
Cao J et al. [3]	China	SC, RS	Zhongnan Hospital	Jan 3-Feb 1	ICU (17.6)	102	48.0	52.7 (22.6)	16.7
Chen R et al. [4]	China	MC, RS	A total of 575 hospitals across mainland China	Dec 11 ^b^-Jan 31	ICU (6.2)	1590	42.7	48.9 (16.3)	3.1
Chen T et al. (a) [5]	China	SC, RS	Tongji Hospital	Jan13-Feb 12	MI, SI, and CI	274	37.6	58.7 (19.4)	41.2
Chen T et al. (b) [6]	China	SC, RS	Zhongnan Hospital	Jan 1-Feb 10	SI (36) and CI (16.7)	203	46.8	54.3 (20.2)	12.8
Cheng Y et al. [7]	China	SC, RS	Tongji Hospital	Jan28-Feb 11	SI (42.7)	701	47.6	61.3 (15.6)	16.1
Deng Y et al. [8]	China	MC, RS	Tongji Hospital and Central Hospital of Wuhan	Jan 1-Feb 21	SI (87.2)	225	44.9	NA	48.4
Du RH et al. [9]	China	SC, PS	Wuhan Pulmonary Hospital	Dec 25 ^b^ -Feb 7	NA	179	45.8	57.6 (13.7)	11.7
Gao L et al. [10]	China	SC, RS	Hubei General Hospital	NA	SI (100)	54	55.6	60.4 (16.1)	33.3
Grasselli L et al. [11]	Italy	MC, RS	ICUs in 72 hospitals	Feb 20-Mar18	ICU (100)	1591	18.0	63.0 (10.4)	25.5
Guan WJ et al. [12]	China	MC, RS	A total of 575 hospitals across mainland China	Dec 11 ^b^ -Jan 31	ICU (6.2)	1590	42.7	48.9 (16.3)	3.1
Guo T et al. [13]	China	SC, RS	Seventh Hospital of Wuhan City	Jan 23-Feb 23	NA	187	51.3	58.5 (14.7)	23.0
He XW et al. [14]	China	SC, RS	Tongji Hospital	Feb 3-Feb 24	SI and CI (100)	56	37.0	67.4 (11.0)	46.4
Hu H et al. [15]	China	SC, RS	Renmin Hospital of Wuhan University	Feb 7-Mar 7	CI (100)	105	49.1	60.8 (16.3)	18.1
Li J et al. [16]	China	SC, RS	Central Hospital of Wuhan	Jan 1- Mar 3	NA	658	54.9	55.5 (18.6)	9.7
Li X et al. [17]	China	SC, RS	Tongji Hospital	Jan 26-Feb 5	SI (100)	268	43.1	63.7 (13.4)	32.5
Liang WH et al. [18]	China	MC, RS	A total of 575 hospitals across mainland China	Dec 11 ^b^ -Jan 31	ICU (6.2)	1590	42.7	48.9 (16.3)	3.1
Liu Y et al. (a) [19]	China	SC, RS	Zhongnan Hospital	Jan 1-Feb 29	NA	245	53.5	53.9 (16.9)	13.5
Liu Y et al. (b) [20]	China	SC, RS	Central Hospital of Wuhan	Jan 1-Mar 1	NA	383	57.7	47.0 (20.1)	12.8
Luo M et al. [21]	China	SC, RS	Wuhan Tongren Hospital	Jan 17-Feb 25	SI and CI (32.0)	475	51.4	61.7 (14.9)	14.5
Miyashita H et al. [22]	USA	SC, MC	Mount Sinai Health System in New York City	Mar 1-Apr 6	NA	5688	NA	NA	9.7
Peng YD et al. [23]	China	SC, RS	Union Hospital	Jan 20-Feb 15	CI (14.3)	112	52.7	61.3 (9.01)	15.2
Richardson S et al. [24]	USA	MC, RC	A total of 12 hospitals in the USA	Mar 1-Apr 4	NA	2634	39.7	63.3 (17.1)	21.0
Shi S et al. [25]	China	SC, RS	Renmin Hospital of Wuhan University	Jan 20- Feb 10	NA	416	50.7	61.0 (12.5)	13.7
Tang N et al. (a) [26]	China	SC, RS	Tongji Hospital	Jan 1- Feb 13	SI (100)	449	40.3	65.1 (12.0)	29.8
Tang N et al. (b) [27]	China	SC, RS	Tongji Hospital	Jan 1-Feb 3	SI (100)	183	46.4	54.1 (16.2)	11.5
Wang L et al. (a) [28]	China	SC, RS	Renmin Hospital of Wuhan University	Jan 31-Feb 5	SI and CI (61.9)	202	56.4	61.3 (14.2)	16.3
Wang L et al. (b) [29]	China	SC, RS	Renmin Hospital of Wuhan University	Jan 1- Feb 6	MI (29.5), SI (46.9), and CI (23.6)	339	51.0	70.0 (8.19)	19.2
Wu C et al. [30]	China	SC, RS	Wuhan Jinyintan Hospital	Dec 25 ^b^ -Jan 26	ICU (26.4)	201	36.3	51.3 (12.7)	21.9
Xie J et al. [31]	China	SC, RS	Union Hospital	Jan 28-Feb 28	MI (30.7), SI (52.1), and CI (17.1)	140	48.6	58.3 (15.7)	25.7
Xu B et al. [32]	China	SC, RS	Hubei Provincial Hospital of Traditional Chinese and Western Medicine	Dec 26 ^b^-Mar 1	SI (24.1), CI (24.1)	187	44.9	60.5 (16.8)	15.0
Yang X et al. (a) [33]	China	SC, RC	Wuhan Jin Yintan hospital	Dec ^b^ -Feb 25	NA	1476	47.4	57.0 (14.8)	16.1
Yang X et al. (b) [34]	China	SC, RC	Wuhan Jin Yintan hospital	Dec 24 ^b^ -Jan 26	CI (100)	52	32.7	59.7 (13.3)	61.5
Yao Q et al. [35]	China	SC, RC	Huanggang Central Hospital	Jan 30-Feb 11	SI (23.1)	108	60.2	49.0 (15.8)	11.1
Zhang J et al. [36]	China	SC, RS	Renmin Hospital of Wuhan University	Jan 11-Feb 6	MI (37.8), SI (47.5), and CI (14.2)	663	51.6	56.2 (18.6)	3.77
Zhang L et al. [37]	China	SC, RC	Wuhan Asia General Hospital	Jan 12-Mar 15	NA	343	50.7	59.7 (15.6)	3.8
Zhou F et al. [38]	China	MC, RC	Jinyintan Hospital and Wuhan Pulmonary Hospital	Dec 29 ^b^ -Jan 31	ICU (26.2), SI (35), and CI (28)	191	37.7	56.3 (15.7)	28.3

*Abbreviations*: *CI* critically ill, *ICU* intensive care unit, *MC* multicenter, *MI* moderately ill, *NA* not available, *PS* prospective study, *RS* retrospective study, *SI* severely ill

^a^ The default year is 2020 unless otherwise noted

^b^ The year is 2019

^c^ Values are expressed as means ± standard deviations. For studies that presented data as medians and interquartile ranges, we calculated the means and standard deviations based on the formulas by Wan et al. [42]

### Meta-analyses of unadjusted estimates

**Fig. 2:**
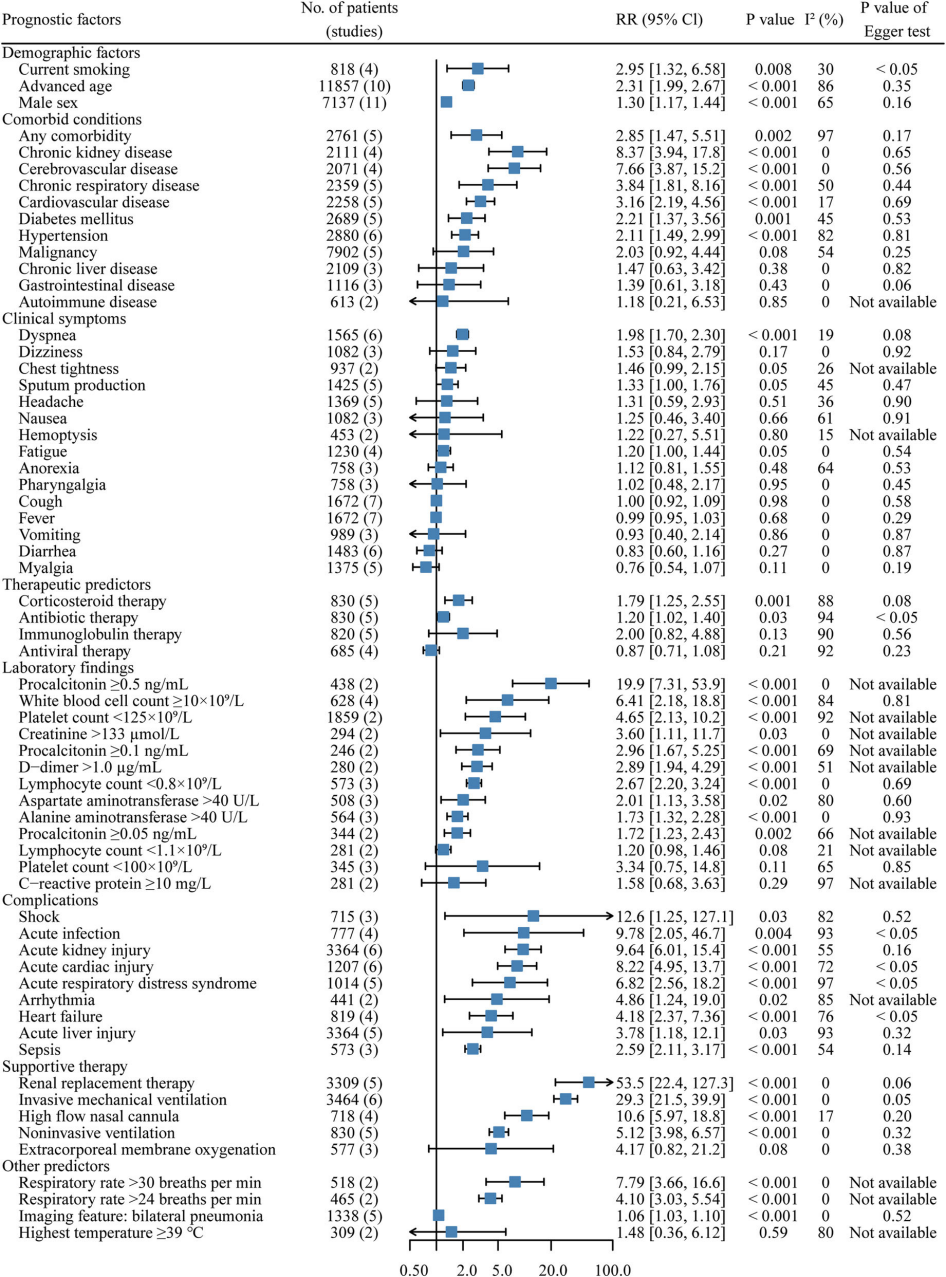
Pooled analyses of predictors for mortality in COVID-19 patients stratified by risk factor type

We conducted meta-analyses of unadjusted estimates for 106 potential predictors of mortality in patients with COVID-19 (Table 2 and Fig. 2). All individual forest plots and further details are presented in Table S2-S107 and Figure S1-S106 in Additional file 2.

**Table 2:** Continuous variables and risk of mortality in patients with COVID-19

Variables	No. of patients (studies)	MD (95% Cl)	*P* value	I^2^ (%)	*P* value of Egger test
Age, years	1829 (8)	13.9 [8.95, 18.9]	< 0.001	89	0.20
Time from illness onset to hospital admission, days	1036 (6)	0.62 [− 0.04, 1.27]	0.07	0	0.20
Respiratory rate, breaths per min	922 (5)	2.94 [1.10, 4.79]	0.002	70	0.84
Heart rate, beat per min	974 (6)	3.91 [1.09, 6.72]	0.006	9	0.28
Partial pressure of oxygen, mm Hg	358 (3)	−28.7 [−52.7, −4.65]	0.02	89	0.46
Partial pressure of carbon dioxide, mm Hg	358 (3)	−5.03 [−6.95, −3.12]	< 0.001	0	0.36
Peripheral oxygen saturation	491 (3)	−7.45 [−15.9, 0.96]	0.08	96	0.52
Partial pressure of oxygen: fraction of inspired oxygen	410 (4)	−122.6 [− 198.2, −47.0]	0.001	92	0.12
Acute Physiology and Chronic Health Evaluation II score	77 (2)	3.86 [1.90, 5.81]	< 0.001	0	Not available
Sequential Organ Failure Assessment score	216 (2)	3.48 [3.06, 3.90]	< 0.001	0	Not available
Laboratory findings					
White blood cell count, ×10^9^/L	1178 (7)	3.53 [2.53, 4.54]	< 0.001	48	0.61
Neutrophil count, ×10^9^/L	1178 (7)	3.86 [2.74, 4.99]	< 0.001	61	0.32
Lymphocyte count, ×10^9^/L	1178 (7)	−0.34 [−0.47, − 0.21]	< 0.001	78	0.18
Monocyte count, ×10^9^/L	919 (4)	−0.06 [− 0.11, − 0.01]	0.02	62	0.95
Platelet count, ×10^9^/L	1029 (5)	−34.0 [−57.1, −10.9]	0.004	59	0.44
Hemoglobin, g/L	829 (4)	0.54 [−2.49, 3.58]	0.73	8	0.32
Albumin, g/L	694 (5)	−4.05 [−6.51, −1.59]	0.001	87	0.51
Total bilirubin, μmol/L	639 (4)	4.01 [3.04, 4.99]	< 0.001	0	0.12
Alanine aminotransferase, U/L	1178 (7)	3.58 [−0.72, 7.87]	0.10	60	0.43
Aspartate aminotransferase, U/L	1153 (6)	14.8 [9.56, 20.1]	< 0.001	57	0.68
Creatinine, μmol/L	1178 (7)	18.0 [10.1, 25.9]	< 0.001	64	0.42
Blood urea nitrogen, mmol/L	419 (2)	3.94 [1.97, 5.90]	< 0.001	80	Not available
Urea, mmol/L	500 (2)	3.41 [1.81, 5.02]	< 0.001	71	Not available
Prothrombin time, seconds	1273 (5)	0.98 [0.74, 1.22]	< 0.001	0	0.68
Activated partial thromboplastin time, seconds	953 (4)	−0.47 [−3.31, 2.37]	0.75	91	0.71
D-dimer, mg/L	1353 (7)	5.26 [3.58, 6.93]	< 0.001	31	0.92
C-reactive protein, mg/L	1178 (7)	55.9 [27.3, 84.5]	< 0.001	92	0.39
Procalcitonin, ng/mL	1029 (6)	0.27 [0.14, 0.40]	< 0.001	76	0.27
Ferritin, μg/L	435 (2)	912.1 [705.2, 1119.0]	< 0.001	25	Not available
Lactate dehydrogenase, U/L	829 (4)	225.6 [153.8, 297.3]	< 0.001	81	0.45
Creatine kinase, U/L	1029 (6)	59.3 [26.2, 92.5]	< 0.001	75	0.66
γ-glutamyl transpeptidase, U/L	453 (2)	4.91 [−16.5, 26.4]	0.65	92	Not available
Erythrocyte sedimentation rate, mm/h	490 (3)	9.01 [3.85, 14.2]	< 0.001	0	0.58
Creatine kinase-MB, U/L	670 (4)	3.11 [0.84, 5.37]	0.007	74	0.17
N-terminal pro-brain natriuretic peptide, pg/mL	476 (2)	865.7 [690.2, 1041.2]	< 0.001	0	Not available
Hypersensitive cardiac troponin I, ng/mL	804 (3)	68.6 [29.3, 107.9]	< 0.001	87	0.32
Myoglobin, ng/mL	381 (2)	170.2 [91.4, 249.1]	< 0.001	0	Not available
Cystatin C, mg/L	186 (2)	0.28 [0.15, 0.42]	< 0.001	0	Not available
Interleukin-6, pg/mL	974 (5)	47.8 [10.6, 85.0]	0.01	96	0.09
CD3+ cell count, /μL	306 (2)	− 315.1 [− 362.2, − 268.0]	< 0.001	0	Not available
CD4+ cell count, /μL	645 (3)	− 168.5 [− 196.1, −140.8]	< 0.001	20	0.02
CD8+ cell count, /μL	645 (3)	−116.3 [− 139.2, −93.5]	< 0.001	47	0.62

Compared with survivors, the mean age of the deceased patients was significantly higher (WMD, 13.9; 95% CI, 8.95 to 18.9). Advanced age (age ≥ 60–70 years) was associated with a significant increase in the risk of mortality (RR, 2.31; 95% CI, 1.99 to 2.67). Male sex was also correlated with a higher death rate than female sex (RR, 1.30; 95% CI, 1.17 to 1.44).

Patients with any comorbidity had a 2.85-fold higher risk of mortality than patients without comorbidities (RR, 2.85; 95% CI, 1.47 to 5.51). The highest mortality risk was observed in patients with chronic kidney disease (RR, 8.37; 95% CI, 3.94 to 17.8), followed by cerebrovascular disease (RR, 7.66; 95% CI, 3.87 to 15.2), chronic respiratory disease (RR, 3.84; 95% CI, 1.81 to 8.16), cardiovascular disease (RR, 3.16; 95% CI, 2.19 to 4.56), diabetes mellitus (RR, 2.21; 95% CI, 1.37 to 3.56) and hypertension (RR, 2.11; 95% CI, 1.49 to 2.99). However, other comorbidities were not associated with survival after pooled analyses.

Symptoms of dyspnea (RR, 1.98; 95% CI, 1.70 to 2.30) were more common in the nonsurvivor group than in the survivor group. No associations between the remaining clinical symptoms and mortality were observed. Antiviral agents and immunoglobulin therapies were not associated with mortality. However, patients who received glucocorticoids (RR, 1.79; 95% CI, 1.25 to 2.55) or antibiotics (RR, 1.20; 95% CI, 1.02 to 1.40) were more likely to die than those who did not.

The values of the following laboratory parameters were significantly higher in the deceased patients than in survivors: white blood cells (WBCs), neutrophils (NEUs), total bilirubin (TBIL), aspartate aminotransferase (AST), creatinine (Cr), blood urea nitrogen (BUN), urea, prothrombin time (PT), D-dimer, C-reactive protein (CRP), procalcitonin (PCT), ferritin, lactate dehydrogenase (LDH), creatine kinase (CK), the erythrocyte sedimentation rate (ESR), creatine kinase-MB (CK-MB), N-terminal pro-brain natriuretic peptide (NT-proBNP), hypersensitive cardiac troponin I (hs-cTnI), myoglobin, cystatin C, and interleukin-6 (IL-6). The lymphocyte (LYM), monocyte (MON), platelet (PLT), albumin (ALB), CD3+, CD4+, and CD8+ cell counts were significantly lower in the nonsurvivors than in the survivors. Moreover, the following abnormal laboratory parameters were more prevalent in the nonsurvivor group than in the survivor group: WBCs ≥10 × 10^9^/L, LYMs < 0.8 × 10^9^/L, PLTs < 125 × 10^9^/L, alanine aminotransferase (ALT) > 40 U/L, AST > 40 U/L, Cr ≥133 μmol/L, PCT ≥0.5 ng/mL (or ≥ 0.1 ng/mL and ≥ 0.05 ng/mL) and D-dimer ≥1.0 μmol/L.

Patients suffering from any comorbidity had a significantly increased risk of mortality, with RRs ranging from 2.59 to 12.6. The highest mortality risk was associated with shock (RR, 12.6; 95% CI, 1.25 to 127.1), followed by superinfection (RR, 9.78; 95% CI, 2.05 to 46.7), acute kidney injury (AKI, RR, 9.64; 95% CI, 6.01 to 15.4), acute cardiac injury (RR, 8.22; 95% CI, 4.95 to 13.7), acute respiratory distress syndrome (ARDS, RR, 6.82; 95% CI, 2.56 to 18.2), arrhythmia (RR, 4.86; 95% CI, 1.24 to 19.0), heart failure (RR, 4.18; 95% CI, 2.37 to 7.36), acute liver injury (RR, 3.78; 95% CI, 1.18 to 12.1) and sepsis (RR, 2.59; 95% CI, 2.11 to 3.17).

Oxygen treatment was applied more often in nonsurvivors than in survivors (high flow nasal cannula, RR, 10.6; 95% CI, 5.97 to 18.8; noninvasive ventilation, RR, 5.12; 95% CI, 3.98 to 6.57; invasive ventilation, RR, 29.3; 95% CI, 21.5 to 39.9). Renal replacement therapy was also observed to be more prevalent in the deceased patients than in the surviving patients (RR, 53.5; 95% CI, 22.4 to 127.3). No correlation between the use of extracorporeal membrane oxygenation (ECMO) and mortality was observed.

A significant difference between nonsurvivors and survivors was also observed for the following variables: respiratory rate, heart rate, respiratory rate > 30 (or 24) breaths per min, partial pressure of oxygen (PaO_2_), partial pressure of carbon dioxide (PaCO_2_), ratio of partial pressure of oxygen to fraction of inspired oxygen (PaO_2_/FiO_2_), bilateral pneumonia, Acute Physiology and Chronic Health Evaluation II (APACHE II) score, and Sequential Organ Failure Assessment (SOFA) score.

Significant heterogeneity was observed in the analyses of 54 tested predictors. Sensitivity analyses did not change the conclusions about the most tested variables, whereas the results for the following 14 tested predictors were inconsistent: preexisting malignancy, symptoms of anorexia, antiviral therapy, immunoglobulin therapy, CRP ≥10 mg/L, shock, acute liver injury, MON and PLT count, activated partial thromboplastin time (APTT), γ-glutamyl transpeptidase, PaO_2_, SpO_2_, and IL-6 (Additional file 2: Table S108-S109). No significant publication bias was observed for any risk factors except for two predictors, current smoking status and antibiotic therapy (Table 2 and Fig. 2).

### Qualitative analysis of adjusted estimates

Adjusted data regarding mortality due to COVID-19 infection were available in 16 studies [4, 5, 7, 9, 10, 12, 17–19, 21, 26, 28, 29, 31, 37, 38] (Table 3). A multivariate Cox regression model was used in ten studies, and a multivariate logistic regression model was applied in six studies. Demographic characteristics highlighted as predictors for increased risk of mortality were advanced age [4, 9, 18, 26, 28, 29, 38], male sex [5, 17] and presence of a comorbidity [4, 5, 9, 12, 18, 21, 29, 31], such as cardio-cerebrovascular disease [4, 9, 29], chronic obstructive pulmonary disease (COPD) [12, 29], diabetes [12], hypertension [12], and malignancy [12]. In two studies, the symptom of dyspnea was correlated with a greater risk of death [4, 31].

**Table 3:** Summary of identified risk factors for increased risk of mortality in studies using regression models

Study	Setting (sample size)	Regression model	Significant risk factors (effect estimate, 95% CI)
Chen R et al. [4]	A total of 575 hospitals across mainland China (1590)	Multivariate Cox regression	Age ≥ 75 years [HR 7.86, 2.44–25.35], age between 65 and 74 years [HR 3.43, 1.24–9.50], CHD [HR 4.28, 1.14–16.13], cerebrovascular disease [HR 3.10, 1.07–8.94], dyspnea [HR 3.96, 1.42–11.0], PCT > 0.5 ng/mL [HR 8.72, 3.42–22.28], and AST > 40 U/L [HR 2.20, 1.10–6.73]
Chen T et al. (b) [5]	Zhongnan Hospital, China (203)	Stepwise multivariate logistic regression	Male sex [OR 13.8, 1.41–136.1], any comorbidity [OR 16.1, 1.9–133.8], shortness of breath [OR 12.9, 1.8–94.4], and Cr > 105 μmol/L [OR 4.82, 1.16–16.96]
Cheng Y et al. [7]	Tongji Hospital, China (701)	Multivariate Cox regression adjusted for age, sex, disease severity, WBC, and comorbidities	Elevated baseline BUN [HR 4.20, 2.74–6.45], elevated baseline Cr [HR 2.04, 1.32–3.15], Peak Cr > 133 μmol/L [HR 3.09, 1.95–4.87], proteinuria 1+ [HR 2.47, 1.15–5.33], proteinuria 2 + ~ 3+ [HR 6.80, 2.97–15.6], hematuria 1+ [HR 3.05, 1.43–6.49], hematuria 2 + ~ 3+ [HR 8.89, 4.41–17.9], AKI stage 2 [HR 3.53, 1.50–8.27], and AKI stage 3 [HR 4.72, 2.55–8.75]
Du RH et al. [9]	Wuhan Pulmonary Hospital, China (179)	Multivariate logistic regression	Age ≥ 65 years [OR 3.765, 1.146–17.394], CCD [OR 2.464, 0.755–8.044], CD3+ CD8+ T cells ≤75/μL [OR 3.982, 1.132–14.060], and hs-cTnI ≥0.05 ng/mL [OR 4.077, 1.166–14.253]
Gao L et al. [10]	Hubei General Hospital, China (54)	Multivariate Cox proportional hazards regression adjusted for sex and age	NT-proBNP [HR 1.323, 1.119–1.563]
Guan WJ et al. [12]	A total of 575 hospitals across mainland China (1590)	Multivariate Cox regression adjusted for age and smoking status	COPD [HR 2.68, 1.42–5.05], diabetes [HR 1.59, 1.03–2.45], hypertension [HR 1.58, 1.07–2.32], malignancy [HR 3.50, 1.60–7.64], one comorbidity [HR 1.79, 1.16–2.77] and ≥ 2 comorbidities [HR 2.59, 1.61–4.17]
Li X et al. [17]	Tongji Hospital, China (268)	Multivariate Cox regression	Male sex [HR 1.72, 1.05–2.82], age ≥ 65 years [HR 1.72, 1.09–2.73], WBC ≥10 × 10^9^/L [HR 2.04, 1.26–3.31], LDH > 445 U/L [HR 2.00, 1.21–3.30], cardiac injury [HR2.92, 1.80–4.76], hyperglycemia [HR 1.77, 1.11–2.84], and high-dose corticosteroids (vs none) [HR 3.5, 1.79–6.86]
Liang WH et al. [18]	A total of 575 hospitals across mainland China (1590)	Multivariate Cox proportional hazards regression	Age [HR 1.036, 1.021–1.052], any comorbidity [HR 2.132, 1.393–3.261], and time from symptom onset to hospitalization [HR 1.045, 1.013–1.078]
Liu Y et al. [19]	Zhongnan Hospital, China (245)	Multivariate logistic regression adjusted for age, sex, body mass index, comorbidities, smoking status, respiratory rate, ALT, Cr, PT, and D-dimer	Neutrophil-to-lymphocyte ratio [OR 1.08, 1.01–1.14]
Luo M et al. [21]	Wuhan Tongren Hospital, China (475)	Multivariate logistic regression	≥1 comorbidity [OR 29.4, 10.343–83.681] and severe disease [OR 74.364, 15.4–359.712]
Tang N et al. (a) [26]	Tongji Hospital, China (449)	Multivariate logistic regression	Age [OR 1.033, 1.013–1.055], PT [OR 1.107, 1.008–1.215], PLT [0.996, 0.993–0.998], and D-dimer [1.058, 1.028–1.090]
Wang et al. (b) [28]	Renmin Hospital of Wuhan University, China (202)	Multivariate Cox regression adjusted for age, sex, hypertension, cerebrovascular disease, CKD, COPD, and NT-proBNP	Age [HR 1.041, 1.011–1.071], respiratory rate [HR 1.194, 1.113–1.281], and myocardial injury [HR 5.382, 2.404–12.05]
Wang L et al. [29]	Renmin Hospital of Wuhan University, China (339)	Multivariate Cox regression	Age [HR 1.08, 1.06–1.11], cardiovascular disease [HR 2.87, 1.70–4.83], COPD [HR 3.72, 1.94–7.13], and ARDS [HR 50.7, 24.0–107]
Xie J et al. [31]	Union Hospital, China (140)	Multivariate Cox proportional hazards regression adjusted for age and sex	Any comorbidity [HR2.65, 1.07–6.55], SpO_2_ [HR 0.93, 0.91–0.95], SpO_2_ ≤ 90% [HR 47.4, 6.29–357.48], dyspnea [HR 2.60, 1.24–5.43], WBC ≥10 × 10^9^/L [HR 2.56, 1.17–5.63], NEU ≥6 × 10^9^/L [HR 4.29, 1.74–10.6], PLT < 150 × 10^9^/L [HR 2.23, 1.01–4.92], and CRP ≥27.8 mg/L [HR 17.0, 2.25–16.0]
Zhang L et al. [37]	Wuhan Asia General Hospital, China (343)	Multivariate Cox regression adjusted for gender, age, and underlying diseases	D-dimer ≥2 μg/mL [HR 22.4, 2.86–175.7]
Zhou F et al. [38]	Jinyintan Hospital and Wuhan Pulmonary Hospital, China (191)	Multivariate logistic regression	Age [OR 1.10, 1.03–1.17], D-dimer > 1 μg/L (vs ≤0.5 μg/L) [OR 18.4, 2.64–128.6], and SOFA score [OR 5.56, 2.61–12.2]

*Abbreviations*: *AKI* acute kidney injury, *ALT* alanine transaminase, *ARDS* acute respiratory distress syndrome, *AST* aspartate aminotransferase, *BUN* blood urea nitrogen, *CCD* cardio-cerebrovascular disease, *CHD* coronary heart disease, *COPD* chronic obstructive pulmonary disease, *Cr* creatinine, *CRP* C-reactive protein, *HR* hazard ratio, *hs-cTnI* hypersensitive cardiac troponin I, *LDH* lactose dehydrogenase, *NT-proBNP* N-terminal pro-brain natriuretic peptide, *OR* odds ratio, *PCT* procalcitonin, *PLT* platelet count, *PT* prothrombin time, *SOFA* Sequential Organ Failure Assessment, *SpO*_*2*_ oxygen saturation, *NEU* neutrophil count, *WBC* white blood cell count

Decreased PLTs [26, 31] and increased WBCs (≥10 × 10^9^/L) [17, 31], NEUs (≥6 × 10^9^/L) [31], neutrophil-to-lymphocyte ratio [19], PCT (> 0.5 ng/mL) [4], CRP (≥27.8 mg/L) [31], AST (> 40 U/L) [4], hs-cTnI (≥0.05 ng/mL) [9], Cr [5, 7], BUN [7], NT-proBNP [10] and D-dimer [26, 37, 38] were identified as risk factors for mortality. Moreover, CD3+ CD8+ T cells ≤75/μL [9] and the presence of proteinuria and hematuria [7] were also correlated with an increased mortality rate.

Patients suffering from AKI [7], ARDS [29], cardiac injury [17, 28], or hyperglycemia [17] during hospitalization had an increased risk for mortality. Other identified risk factors include shortness of breath [5], SpO_2_ ≤ 90% [31], days from symptom onset to hospitalization [18], severe disease [21], SOFA score [38], and high-dose corticosteroid therapy [17].

## Discussion

To the best of our knowledge, this is the most comprehensive systematic review and meta-analysis evaluating predictors of mortality in patients with COVID-19 conducted to date. Important risk factors associated with an increased fatality rate included older age, male sex, current smoking, baseline comorbidities (especially chronic kidney, respiratory, and cardio-cerebrovascular diseases), symptoms of dyspnea, complications during hospitalization, corticosteroid therapy and a severe condition. Additionally, a series of abnormal biomarkers of hematologic parameters (especially WBC, NEU, and LYM counts), hepatorenal function (especially Cr, BUN, and AST), inflammation (especially PCT, CRP, ferritin, and the ESR), coagulation (especially D-dimer and PT), and cardiovascular injury (especially hs-cTnI and NT-proBNP) were also associated with fatal outcomes.

Similar to the two previous emergences of coronavirus diseases, severe acute respiratory syndrome (SARS) and Middle East respiratory syndrome (MERS), the outbreak of COVID-19 has posed great challenges for public health. Although most COVID-19 cases are mild, patients with severe conditions may quickly progress to ARDS, multiple organ failure and even death. The present study identified predictors of mortality that clinicians and other healthcare providers can consider when discussing the expected prognosis of patients with COVID-19 and thus take appropriate measures.

Advanced age has been identified as an independent risk factor for mortality in SARS [44, 45] and MERS [46, 47]. Our meta-analysis confirmed that older age was also correlated with an increased mortality rate in patients with COVID-19. Several factors might contribute to this mortality risk, including age-related physiological changes, impaired immune function, and preexisting illnesses. In the present meta-analysis, articles that only enrolled children were excluded. Although available studies show that the mortality rate due to COVID-19 in children is relatively low [48, 49], healthcare providers and parents are concerned about the health of children. Future prognostic studies focusing on children with COVID-19 are warranted.

The present meta-analysis found that patients who are current smokers were 2.95 times more likely to die than nonsmokers. Upregulation of angiotensin-converting enzyme 2 (ACE2) expression in airways might explain the increased risk of death in current smokers with COVID-19 infection [50]. The pooled analysis indicated that male sex was associated with a 30% increased risk of mortality among patients with COVID-19. Although the factors accounting for the sex difference in the incidence of death remain unknown, we suggest that smoking might be one of the contributing factors. Of note, the included studies were primarily from China, where the proportion of adult men who smoke (> 50%) is much higher than that the proportion of adult women who smoke (< 3%) [51]. It is possible that the sex differences in the survival rate are due to the different prevalence of smoking in the two sexes. The sex difference in the risk of mortality among patients with COVID-19 may also be related to differential ACE2 expression in males and females related to the X chromosome [52, 53].

Accumulated evidence has shown that COVID-19 infection is more likely to occur in patients with preexisting conditions than in those without preexisting conditions [54]. Similar to SARS [45, 55] and MERS patients [56], preexisting conditions were also found to have an important effect on the prognosis in COVID-19 patients. Our pooled analysis showed that the risk of mortality in COVID-19 patients with any comorbidity was 2.85 times higher than that in those without preexisting conditions. Patients with chronic kidney disease, cerebrovascular disease, respiratory disease, cardiovascular disease, diabetes mellitus or hypertension had approximately 8-fold, 8-fold, 4-fold, 3-fold, 2-fold, and 2-fold higher risks of mortality, respectively, than individuals without these conditions. Considering that these comorbidities are predictors of poor outcomes, optimum control of these conditions may be beneficial for the management of COVID-19. A recent large-sample study investigated the correlation of blood glucose control and outcomes in COVID-19 patients with diabetes [57]. The results indicated that patients with well-controlled blood glucose who maintain glycemic variability within 3.9 to 10.0 mmol/L had significantly improved survival compared to patients with poorly controlled blood glucose [57]. Further studies are still urgently needed to achieve a comprehensive understanding of how specific comorbidities exacerbate COVID-19 disease severity so as to improve clinical outcomes of the disease through precision-targeted management.

The symptoms of COVID-19 infection are nonspecific. Patients with COVID-19 can present with fever, cough, muscle aches, fatigue, headache, gastrointestinal symptoms, and dyspnea [58]. Of these symptoms, dyspnea was significantly associated with an increased mortality rate in the pooled analysis, corroborating the findings of two studies [4, 31] that demonstrated that dyspnea was correlated with a higher mortality rate even after adjustment for age, sex, and other confounding factors. As dyspnea can be easily observed in clinical practice, it may be a valuable predictor to help identify individuals who are at high risk for fatal outcomes and may need additional attention. Additionally, blood gas analysis may be a useful tool to determine the severity of dyspnea. Decreased SpO_2_ may reflect severe dyspnea, indicating an increased mortality risk, as shown in the present meta-analysis.

Dramatically reduced LYM levels as well as CD3, CD4, and CD8 cell counts in the deceased patients suggests that SARS-CoV-2 may act on T lymphocytes, and viral replication contributes to the destruction of T lymphocytes, decreasing immune function. Not surprisingly, patients with poor immune function were more likely to suffer from acute infection than those with normal immune function, and patients suffering from acute infection were more likely to die than those without this complication. In the present meta-analysis, a positive correlation between the PCT level or WBC count and mortality was observed, indicating that an increased WBC count (≥10 × 10^9^/L) may be a useful predictor and that PCT-guided antibiotic therapy might be beneficial in COVID-19 infection.

Compared with that in survivors, serum concentrations of Cr were higher in patients in the deceased group, indicating worse kidney function, although the mean values remained within the normal range. A single-cell transcriptome analysis indicated that the cytopathic effects of SARS-CoV-2 on podocytes and proximal straight tubule cells may contribute to the development of AKI in patients with COVID-19 [59]. Increased baseline Cr or BUN, peak Cr > 133 μmol/L, and the presence of hematuria, hematuria or AKI were identified as independent predictors of in-hospital mortality in a large-sample prospective study evaluating 701 COVID-19 cases [7]. In the present meta-analysis, the development of AKI and increased SCr (> 133 μmol/L) were associated with an approximately 9.6-fold and 3.6-fold increased risk of mortality among patients with COVID-19, respectively. Considering the great impact of renal damage on prognosis, close monitoring of renal function-related parameters is required.

Regarding markers of liver injury, the pooled analysis demonstrated statistically higher levels of AST and TBIL in nonsurvivors than in survivors, and patients with increased AST (> 40 U/L) were approximately twice as likely to die as patients with normal AST values. Although increased ALT (> 40 U/L) was also correlated with an increased risk of death, no significant difference in the mean levels of ALT between nonsurvivors and survivors was observed in our meta-analysis. In a large-sample longitudinal study that evaluated 5771 patients with COVID-19 infection, elevation of AST was correlated with the highest mortality risk compared to other markers of liver injury such as ALT, TBIL and alkaline phosphatase. In addition, the elevation of AST occurred before the elevation of ALT [60]. These findings indicate that AST may be a better liver injury marker for predicting clinical outcomes than the other markers, and frequent monitoring of AST and early detection of liver injury are suggested. Decreased albumin (< 35 g/L) has been identified as an independent predictor of severe infection requiring intensive care unit (ICU) admission in MERS infection [61]. In our study, serum albumin was also significantly lower in the deceased patients than in the surviving patients, indicating that malnutrition might contribute to the adverse outcome of COVID-19 infection and that nutritional support may be beneficial in the management of this disease.

Cytokine storm, also known as hypercytokinemia, refers to the excessive and uncontrolled release of pro-inflammatory cytokines. Huang et al. [62] found markedly higher plasma levels of cytokines in COVID-19 patients requiring ICU admission than in those not treated in the ICU, indicating that cytokines are correlated with disease severity. In the present meta-analysis, numerous inflammatory biomarkers (ESR, CRP, PCT, ferritin, and IL-6) were higher in deceased patients than in survivors, providing further evidence for the presence of a cytokine storm that can contribute to the fatal outcome of COVID-19 patients. Mehta et al. [63] suggested that each COVID-19 patient with a severe condition should be screened for hyperinflammation considering laboratory trends (increasing ferritin, decreasing PLTs or ESR) and HScore [64].

Cardiovascular complications of COVID-19, such as cardiac injury, heart failure, and arrhythmia, were more prevalent in patients who died than in patients who survived. Among them, cardiac injury was correlated with the highest mortality risk and has been widely studied. The possible mechanisms of cardiac injury caused by COVID-19 may involve cardiac stress due to respiratory failure and hypoxemia, direct myocardial infection by the virus, and indirect damage from the systemic inflammatory response [65]. In the present meta-analysis, several indicators of cardiovascular injury, such as hs-cTnI, NT-proBNP, CK-MB and myoglobin, were significantly higher in patients in nonsurvivor group than in those in the survivor group. Increased hs-cTnI and NT-proBNP among COVID-19 patients have been identified as independent risk factors for mortality even after adjustment for age and other confounding factors [9, 10]. Therefore, we suggest that frequent measurement of hs-cTnI and NT-proBNP should be required in the management of COVID-19, especially for patients with preexisting cardio-cerebrovascular disease.

Coagulation dysfunction is common in patients with COVID-19. We identified significantly lower PLTs in patients with a fatal outcome, and thrombocytopenia (PLT < 125 × 10^9^/L) was correlated with a 4.65-fold increased risk of mortality. Increased D-dimer and prolonged PT were also observed more frequently in nonsurvivors than in survivors. These findings indicated that excessive activation of the coagulation cascade and PLTs existed in the progression of COVID-19 infection. The underlying mechanisms of activated coagulation remain unclear but may be due to inflammatory responses induced by SARS-CoV-2 [66]. Coagulation screening, especially the determination of D-dimer and PLT levels, has been suggested.

At present, effective pharmacological interventions for the treatment of COVID-19 are still limited. Antiviral agents have been widely applied in the management of COVID-19 infection. However, there was no significant difference between nonsurvivors and survivors regarding antiviral therapy efficacy. Regarding antiviral therapy, the latest COVID-19 Treatment Guidelines recommended the use of remdesivir in hospitalized COVID-19 cases requiring oxygen support [67]. Corticosteroid treatment for COVID-19 infection is a hot topic. A recently published meta-analysis investigated the impact of corticosteroids on the outcomes of patients with coronavirus infections, including SARS, MERS, and COVID-19 [68]. The results demonstrated that the use of corticosteroids significantly delayed the clearance of the virus and did not improve the survival rate, shorten the hospitalization time, or reduce the ICU admission and mechanical ventilation rate. In the present meta-analysis, we found that corticosteroid treatment was correlated with an elevated risk of mortality in COVID-19 patients, with a pooled RR of 1.79 (95% CI 1.25 to 2.55). Current evidence and our findings further support the recommendations by the Infectious Diseases Society of America (IDSA) denouncing the use of corticosteroids in patients with COVID-19 pneumonia [69]. However, a subset analysis of 84 patients with ARDS secondary to COVID-19 infection found an improved survival rate in patients who received methylprednisolone, indicating that corticosteroid therapy might be beneficial for COVID-19 patients who develop ARDS. One randomized controlled trial performed in the UK (RECOVERY trial) showed that the application of dexamethasone improved survival of severe COVID-19 patients who required respiratory support [70]. However, no survival benefit of dexamethasone was observed in mild COVID-19 cases [70]. Currently, the use of dexamethasone is recommended for severe COVID-19 patients requiring respiratory support, but not for non-hospitalized patients, mild to moderate cases, or hospitalized patients who did not receive oxygen support [67]. It is likely that the beneficial effect of corticosteroid treatment for COVID-19 infection is dependent on correct selection of the timing of administration, dose, and patient.

Patients who have indications for organ supportive care, such as the need for invasive mechanical ventilation and renal replacement therapy, tend to be sicker than other patients, and this could explain the increased death rate among those patients. Other indicators of severe conditions, such as APACHE II and SOFA scores, can also be used to predict the prognosis of COVID-19 infection. Probably due to small sample size, the effect of ECMO support on the mortality rate of COVID-19 patients was not statistically significant.

The present study has several strengths. First, the current study was performed based on recent guidelines for the systematic review and meta-analysis of prognostic factors. Second, we made our best effort to avoid potential patient overlap by checking the settings and patient recruitment periods. Third, many potential predictors of mortality in COVID-19 patients were tested.

The present study also had several limitations and should be interpreted cautiously. First, only unadjusted prognostic effect estimates were pooled because only a few of the original studies reported adjusted data, different types of effect measures were used (such as odds ratios and hazard ratios), and there was potential overlap of the patients in the included studies. It is possible that the unadjusted estimates of several factors may become nonsignificant after adjustment. Second, the present meta-analysis included substantial heterogeneity in some tested factors that could be explained by differences in patient populations and in the severity of the disease. Third, although a total of 27 studies were included in the meta-analysis, the number of studies available for analysis of each predictor was insufficient to allow meaningful subgroup analyses.

## Conclusions

The present meta-analysis provides evidence of correlations between important prognostic factors and survival in patients with COVID-19. Clinicians and other healthcare providers should consider these factors when discussing the expected prognosis of COVID-19 patients and take appropriate measures accordingly. Further studies are required to provide a better understanding of the pathophysiological mechanisms of the association between these predictors and COVID-19 infection.

## Supplementary Information

**Additional file 1.**

**Additional file 2.**

## Data Availability

All data generated or analyzed during this study are included in this published article and its supplementary information files.

## Abbreviations

ACE2: Angiotensin-converting enzyme 2
AKI: Acute kidney injury
ALB: Albumin
ALT: Alanine aminotransferase
APACHE II: Acute Physiology and Chronic Health Evaluation II
APTT: Activated partial thromboplastin time
ARDS: Acute respiratory distress syndrome
AST: Aspartate aminotransferase
BUN: Blood urea nitrogen
CIs: Confidence intervals
CK: Creatine kinase
CK-MB: Creatine kinase-MB
COPD: Chronic obstructive pulmonary disease
COVID-19: Coronavirus disease 2019
Cr: Creatinine
CRP: C-reactive protein
ECMO: Extracorporeal membrane oxygenation
ESR: Erythrocyte sedimentation rate
hs-cTnI: Hypersensitive cardiac troponin I
ICU: Intensive care unit
IDSA: Infectious Diseases Society of America
IL-6: Interleukin-6
LDH: Lactate dehydrogenase
LYM: Lymphocyte
MERS: Middle East respiratory syndrome
MON: Monocyte
NEUs: Neutrophils
NT-proBNP: N-terminal pro-brain natriuretic peptide
PaCO_2_: Partial pressure of carbon dioxide
PaO_2_: Partial pressure of oxygen
PaO_2_/FiO_2_: Ratio of partial pressure of oxygen to fraction of inspired oxygen
PCT: Procalcitonin
PLT: Platelet
PRISMA: Preferred Reporting Items for Systematic Reviews and Meta-Analyses
PT: Prothrombin time
QUIPS: Quality in Prognostic Factor Studies
RRs: Risk ratios
SOFA: Sequential Organ Failure Assessment
SARS: Severe acute respiratory syndrome
SARS-CoV-2: Severe acute respiratory syndrome coronavirus 2
TBIL: Total bilirubin
WBCs: White blood cells
WMDs: Weighted mean differences
