# Supplementary material for: Predictors of mortality in patients with coronavirus disease 2019: a systematic review and meta-analysis
Source: BMC Infect Dis. 2021 Jul 8;21:663. doi: 10.1186/s12879-021-06369-0 (PMC8264491; doi:10.1186/s12879-021-06369-0)
Supplement: Supplementary file 1 — Additional file 1. [file 12879_2021_6369_MOESM1_ESM.docx]

**Additional file 1**

Appendix 1. Details of electronic Chinese databases used in the meta-analysis

| Database | Chinese National Knowledge Infrastructure (CNKI) | Wanfang database | VIP database |
| --- | --- | --- | --- |
| URL | www.cnki.net | www.wanfangdata.com | www.cqvip.com |
| Developer | Tsinghua University | Wanfang Data Inc. | cqvip.com Inc. |
| Journals | 1000+ ^a^ | 1024 ^a^ | 1818 ^a^ |
| Articles | 3308164 | 8896299 (as of 4 Jan 2008) | 2900000+ |
| Proceedings | 145457 ^b^ | 124646 | Not available |
| Theses | 47030 ^c^ | 144318 | Not available |
| Start date | 1979 | 1997 | 1989 |
| Update | Daily-satellite; Monthly-disc | Weekly | Unclear |
| Citation | Free access online | Free access online | Free access online |
| Chinese abstract | Free access online | Free access online | Free access online |
| English abstract | Available in the full-text PDF file | Available in the full-text PDF file | Available in the full-text PDF file |
| Full text | Subscription only | Subscription only | Subscription only |
| Record selection | Search results displayed per page (Max 10/page) | Search results displayed per page (Max 20/page) | Search results displayed per page (Max 50/page) |
| Download format | Tagged text (unlimited/file) | Copy and paste (unlimited/file) | Tagged text  (50/file) |

Note: This table was adapted from the study of Xia J, et al. [Health Info Libr J. 2008; 25(1):55-61. PMID: 18251914)].

^a^ Medicine and hygiene subset.

^b^ China Proceedings of Conference database (CPCD) subset.

^c^ Doctorate/Masters dissertations database (CDMD) subset.
