# Supplementary material for: Predictors of mortality in patients with coronavirus disease 2019: a systematic review and meta-analysis
Source: BMC Infect Dis. 2021 Jul 8;21:663. doi: 10.1186/s12879-021-06369-0 (PMC8264491; doi:10.1186/s12879-021-06369-0)
Supplement: Supplementary file 2 — Additional file 2. [file 12879_2021_6369_MOESM2_ESM.pdf]

Supplemental file

Title: Predictors of mortality in patients with coronavirus disease 2019: a systematic review and meta-analysis

CONTENTS

Notes and Abbreviations 7

TABLES

Table S1. Quality Assessments of the Included Studies 8

Demographic Factors

Table S2. Data of Age between Nonsurvivors and Survivors 9

Table S3. Data of Association between Advanced Age and Mortality 9

Table S4. Data of Association between Male Sex and Mortality 9

Table S5. Data of Association between Current Smoking and Mortality 10

Comorbid Conditions

Table S6. Data of Association between Preexisting Any Comorbidity and Mortality 10

Table S7. Data of Association between Preexisting Chronic Kidney Disease and Mortality 10

Table S8. Data of Association between Cerebrovascular Disease and Mortality 10

Table S9. Data of Association between Preexisting Chronic Respiratory Disease and Mortality 10

Table S10. Data of Association between Preexisting Cardiovascular Disease and Mortality 11

Table S11. Data of Association between Preexisting Diabetes Mellitus and Mortality 11

Table S12. Data of Association between Preexisting Hypertension and Mortality 11

Table S13. Data of Association between Preexisting Malignancy and Mortality 11

Table S14. Data of Association between Preexisting Gastrointestinal Disease and Mortality 11

Table S15. Data of Association between Preexisting Chronic Liver Disease and Mortality 12

Table S16. Data of Association between Preexisting Autoimmune Disease and Mortality 12

Clinical Symptoms

Table S17. Data of Association between Symptoms of Fever and Mortality 12

Table S18. Data of Association between Symptoms of Cough and Mortality 12

Table S19. Data of Association between Symptoms of Dyspnea and Mortality 12

Table S20. Data of Association between Symptoms of Chest Tightness and Mortality 13

Table S21. Data of Association between Symptoms of Sputum Production and Mortality 13

Table S22. Data of Association between Symptoms of Fatigue and Mortality 13

Table S23. Data of Association between Symptoms of Anorexia and Mortality 13

Table S24. Data of Association between Symptoms of Diarrhea and Mortality 13

Table S25. Data of Association between Symptoms of Myalgia and Mortality 13

Table S26. Data of Association between Symptoms of Dizziness and Mortality 14

Table S27. Data of Association between Symptoms of Nausea and Mortality 14

Table S28. Data of Association between Symptoms of Headache and Mortality 14

Table S29. Data of Association between Symptoms of Vomiting and Mortality 14

Table S30. Data of Association between Symptoms of Pharyngalgia and Mortality 14

Table S31. Data of Association between Symptoms of Hemoptysis and Mortality 14

Complications

Table S32. Data of Association between Shock and Mortality 15
Table S33. Data of Association between Acute Infection and Mortality 15
Table S34. Data of Association between Acute Kidney Injury and Mortality 15
Table S35. Data of Association between Acute Cardiac Injury and Mortality 15
Table S36. Data of Association between Acute Respiratory Distress Syndrome and Mortality 15
Table S37. Data of Association between Arrhythmia and Mortality 16
Table S38. Data of Association between Heart Failure and Mortality 16
Table S39 Data of Association between Acute Liver Injury and Mortality 16
Table S40. Data of Association between Sepsis and Mortality 16

Supportive Therapy

Table S41. Data of Association between Renal Replacement Therapy and Mortality 16
Table S42. Data of Association between Invasive Mechanical Ventilation and Mortality 16
Table S43. Data of Association between Noninvasive Ventilation and Mortality 17
Table S44. Data of Association between High Flow Nasal Cannula and Mortality 17
Table S45. Data of Association between ECMO and Mortality 17

Therapeutic Predictors

Table S46. Data of Association between Corticosteroid Therapy and Mortality 17
Table S47. Data of Association between Antibiotic Therapy and Mortality 17
Table S48. Data of Association between Immunoglobulin Therapy and Mortality 18
Table S49. Data of Association between Antiviral Therapy and Mortality 18

Laboratory Findings

Table S50. Data of White Blood Cell Count between Nonsurvivors and Survivors 18
Table S51. Data of Neutrophil Count between Nonsurvivors and Survivors 18
Table S52. Data of Lymphocyte Count between Nonsurvivors and Survivors 19
Table S53. Data of Monocyte Count between Nonsurvivors and Survivors 19
Table S54. Data of Platelet Count between Nonsurvivors and Survivors 19
Table S55. Data of Hemoglobin between Nonsurvivors and Survivors 19
Table S56. Data of Albumin between Nonsurvivors and Survivors 20
Table S57. Data of Total Bilirubin between Nonsurvivors and Survivors 20
Table S58. Data of ALT between Nonsurvivors and Survivors 20
Table S59. Data of AST between Nonsurvivors and Survivors 20
Table S60. Data of Creatinine between Nonsurvivors and Survivors 21
Table S61. Data of Blood Urea Nitrogen between Nonsurvivors and Survivors 21
Table S62. Data of Urea between Nonsurvivors and Survivors 21
Table S63. Data of Prothrombin Time between Nonsurvivors and Survivors 21
Table S64. Data of APTT between Nonsurvivors and Survivors 21
Table S65. Data of C-reactive Protein between Nonsurvivors and Survivors 22
Table S66. Data of D-dimer between Nonsurvivors and Survivors 22
Table S67. Data of Procalcitonin between Nonsurvivors and Survivors 22
Table S68. Data of Ferritin between Nonsurvivors and Survivors 22
Table S69. Data of Lactate Dehydrogenase between Nonsurvivors and Survivors 23
Table S70. Data of Creatine Kinase between Nonsurvivors and Survivors 23
Table S71. Data of γ-glutamyl Transpeptidase between Nonsurvivors and Survivors 23
Table S72. Data of ESR between Nonsurvivors and Survivors 23

Table S73. Data of Creatine Kinase-MB between Nonsurvivors and Survivors 23
Table S74. Data of NT-proBNP between Nonsurvivors and Survivors 24
Table S75. Data of hs-cTnI between Nonsurvivors and Survivors 24
Table S76. Data of Myoglobin between Nonsurvivors and Survivors 24
Table S77. Data of Cystatin C between Nonsurvivors and Survivors 24
Table S78. Data of Interleukin-6 between Nonsurvivors and Survivors 24
Table S79. Data of CD3+ Cell Count between Nonsurvivors and Survivors 24
Table S80. Data of CD4+ Cell Count between Nonsurvivors and Survivors 25
Table S81. Data of CD8+ Cell Count between Nonsurvivors and Survivors 25
Table S82. Data of Association between Increased White Blood Cell Count (≥10 $\times 10^9$/L) and Mortality 25
Table S83. Data of Association between Decreased Lymphocyte Count (<0.8 $\times 10^9$/L) and Mortality 25
Table S84. Data of Association between Decreased Lymphocyte Count (<1.1 $\times 10^9$/L) and Mortality 25
Table S85. Data of Association between Decreased Platelet Count (<100 $\times 10^9$/L) and Mortality 25
Table S86. Data of Association between Decreased Platelet Count (<125 $\times 10^9$/L) and Mortality 26
Table S87. Data of Association between Increased ALT (>40 U/L) and Mortality 26
Table S88. Data of Association between Increased AST (>40 U/L) and Mortality 26
Table S89. Data of Association between Increased Creatinine (>133 μmol/L) and Mortality 26
Table S90. Data of Association between Increased Procalcitonin (≥0.5 ng/mL) and Mortality 26
Table S91. Data of Association between Increased Procalcitonin (≥0.1 ng/mL) and Mortality 26
Table S92. Data of Association between Increased Procalcitonin (≥0.05 ng/mL) and Mortality 26
Table S93. Data of Association between Increased C-reactive Protein (≥10 mg/L) and Mortality 26
Table S94. Data of Association between Increased D-dimer (>1.0 μg/mL) and Mortality 27
Table S95. Data of Association between Respiratory Rate ≥30 breaths per min and Mortality 27
Table S96. Data of Association between Respiratory Rate ≥24 breaths per min and Mortality 27

Other Predictors

Table S97. Data of Association between Bilateral Pneumonia and Mortality 27
Table S98. Data of Association between Highest Temperature ≥39 °C and Mortality 27
Table S99. Data of Respiratory Rate between Nonsurvivors and Survivors 27
Table S100. Data of Heart Rate between Nonsurvivors and Survivors 28
Table S101. Data of PaO_2 between Nonsurvivors and Survivors 28
Table S102. Data of $PaCO_2$ between Nonsurvivors and Survivors 28
Table S103. Data of SpO_2 between Nonsurvivors and Survivors 28
Table S104. Data of PaO_2/FiO_2 between Nonsurvivors and Survivors 28
Table S105. Data of APACHE II Score between Nonsurvivors and Survivors 29
Table S106. Data of SOFA Score between Nonsurvivors and Survivors 29
Table S107 Data of Time from Illness Onset to Hospital Admission between Nonsurvivors and Survivors 29

Sensitivity Analyses

Table S108. Sensitivity Analyses of Dichotomous Prognostic Factors 30
Table S109. Sensitivity Analyses of Continuous Prognostic Factors 31

FIGURES

Demographic Factors

Figure S1. Forest plot of mean difference in age between nonsurvivors and survivors 32
Figure S2. Forest plot of association between advanced age and mortality 32
Figure S3. Forest plot of association between male sex and mortality 32

Figure S4. Forest plot of association between current smoking and mortality........33
Comorbid Conditions
Figure S5. Forest plot of association between preexisting any comorbidity and mortality........33
Figure S6. Forest plot of association between preexisting chronic kidney disease and mortality........33
Figure S7. Forest plot of association between cerebrovascular disease and mortality........33
Figure S8. Forest plot of association between preexisting chronic respiratory disease and mortality........34
Figure S9. Forest plot of association between preexisting cardiovascular disease and mortality........34
Figure S10. Forest plot of association between preexisting diabetes mellitus and mortality........34
Figure S11. Forest plot of association between preexisting hypertension and mortality........34
Figure S12. Forest plot of association between preexisting malignancy and mortality........35
Figure S13. Forest plot of association between preexisting gastrointestinal disease and mortality........35
Figure S14. Forest plot of association between preexisting chronic liver disease and mortality........35
Figure S15. Forest plot of association between preexisting autoimmune disease and mortality........35
Clinical Symptoms
Figure S16. Forest plot of association between symptoms of fever and mortality........36
Figure S17. Forest plot of association between symptoms of cough and mortality........36
Figure S18. Forest plot of association between symptoms of dyspnea and mortality........36
Figure S19. Forest plot of association between symptoms of chest tightness and mortality........36
Figure S20. Forest plot of association between symptoms of sputum production and mortality........37
Figure S21. Forest plot of association between symptoms of fatigue and mortality........37
Figure S22. Forest plot of association between symptoms of anorexia and mortality........37
Figure S23. Forest plot of association between symptoms of diarrhea and mortality........37
Figure S24. Forest plot of association between symptoms of myalgia and mortality........38
Figure S25. Forest plot of association between symptoms of dizziness and mortality........38
Figure S26. Forest plot of association between symptoms of nausea and mortality........38
Figure S27. Forest plot of association between symptoms of headache and mortality........38
Figure S28. Forest plot of association between symptoms of vomiting and mortality........39
Figure S29. Forest plot of association between symptoms of pharyngalgia and mortality........39
Figure S30. Forest plot of association between symptoms of hemoptysis and mortality........39
Complications
Figure S31. Forest plot of association between shock and mortality........39
Figure S32. Forest plot of association between acute infection and mortality........39
Figure S33. Forest plot of association between acute kidney injury and mortality........40
Figure S34. Forest plot of association between acute cardiac injury and mortality........40
Figure S35. Forest plot of association between acute respiratory distress syndrome and mortality........40
Figure S36. Forest plot of association between arrhythmia and mortality........40
Figure S37. Forest plot of association between heart failure and mortality........41
Figure S38. Forest plot of association between acute liver injury and mortality........41
Figure S39. Forest plot of association between sepsis and mortality........41
Supportive Therapy
Figure S40. Forest plot of association between renal replacement therapy and mortality........41
Figure S41. Forest plot of association between invasive mechanical ventilation and mortality........42
Figure S42. Forest plot of association between noninvasive ventilation and mortality........42
Figure S43. Forest plot of association between high flow nasal cannula and mortality........42
Figure S44. Forest plot of association between ECMO and mortality........42

Therapeutic Predictors

Figure S45. Forest plot of association between corticosteroid therapy and mortality 43
Figure S46. Forest plot of association between antibiotic therapy and mortality 43
Figure S47. Forest plot of association between immunoglobulin therapy and mortality 43
Figure S48. Forest plot of association between antiviral therapy and mortality 43

Laboratory Findings

Figure S49. Forest plot of mean difference in white blood cell count between nonsurvivors and survivors 44
Figure S50. Forest plot of mean difference in neutrophil count between nonsurvivors and survivors 44
Figure S51. Forest plot of mean difference in lymphocyte count between nonsurvivors and survivors 44
Figure S52. Forest plot of mean difference in monocyte count between nonsurvivors and survivors 44
Figure S53. Forest plot of mean difference in platelet count between nonsurvivors and survivors 45
Figure S54. Forest plot of mean difference in hemoglobin between nonsurvivors and survivors 45
Figure S55. Forest plot of mean difference in albumin between nonsurvivors and survivors 45
Figure S56. Forest plot of mean difference in total bilirubin between nonsurvivors and survivors 45
Figure S57. Forest plot of mean difference in ALT between nonsurvivors and survivors 45
Figure S58. Forest plot of mean difference in AST between nonsurvivors and survivors 46
Figure S59. Forest plot of mean difference in creatinine between nonsurvivors and survivors 46
Figure S60. Forest plot of mean difference in blood urea nitrogen between nonsurvivors and survivors 46
Figure S61. Forest plot of mean difference in urea between nonsurvivors and survivors 46
Figure S62. Forest plot of mean difference in prothrombin time between nonsurvivors and survivors 46
Figure S63. Forest plot of mean difference in APTT between nonsurvivors and survivors 47
Figure S64. Forest plot of mean difference in C-reactive protein between nonsurvivors and survivors 47
Figure S65. Forest plot of mean difference in D-dimer between nonsurvivors and survivors 47
Figure S66. Forest plot of mean difference in procalcitonin between nonsurvivors and survivors 47
Figure S67. Forest plot of mean difference in ferritin between nonsurvivors and survivors 47
Figure S68. Forest plot of mean difference in lactate dehydrogenase between nonsurvivors and survivors 48
Figure S69. Forest plot of mean difference in creatine kinase between nonsurvivors and survivors 48
Figure S70. Forest plot of mean difference in γ-glutamyl transpeptidase between nonsurvivors and survivors 48
Figure S71. Forest plot of mean difference in ESR between nonsurvivors and survivors 48
Figure S72. Forest plot of mean difference in creatine kinase-MB between nonsurvivors and survivors 48
Figure S73. Forest plot of mean difference in NT-proBNP between nonsurvivors and survivors 49
Figure S74. Forest plot of mean difference in hs-cTnI I between nonsurvivors and survivors 49
Figure S75. Forest plot of mean difference in myoglobin between nonsurvivors and survivors 49
Figure S76. Forest plot of mean difference in cystatin C between nonsurvivors and survivors 49
Figure S77. Forest plot of mean difference in interleukin-6 between nonsurvivors and survivors 49
Figure S78. Forest plot of mean difference in CD3+ cell count between nonsurvivors and survivors 49
Figure S79. Forest plot of mean difference in CD4+ cell count between nonsurvivors and survivors 50
Figure S80. Forest plot of mean difference in CD8+ cell count between nonsurvivors and survivors 50
Figure S81. Forest plot of association between increased white blood cell count ($\geq 10 \times 10^9$/L) and mortality 50
Figure S82. Forest plot of association between decreased lymphocyte count ($<0.8 \times 10^9$/L) and mortality 50
Figure S83. Forest plot of association between decreased lymphocyte count ($<1.1 \times 10^9$/L) and mortality 50
Figure S84. Forest plot of association between decreased platelet count ($<100 \times 10^9$/L) and mortality 51
Figure S85. Forest plot of association between decreased platelet count ($<125 \times 10^9$/L) and mortality 51
Figure S86. Forest plot of association between increased ALT (>40 U/L) and mortality 51
Figure S87. Forest plot of association between increased AST (>40 U/L) and mortality 51

Figure S88. Forest plot of association between increased creatinine (>133 μmol/L) and mortality 51
Figure S89. Forest plot of association between increased procalcitonin (≥0.5 ng/mL) and mortality 52
Figure S90. Forest plot of association between increased procalcitonin (≥0.1 ng/mL) and mortality 52
Figure S91. Forest plot of association between increased procalcitonin (≥0.05 ng/mL) and mortality 52
Figure S92. Forest plot of association between increased C-reactive protein (≥10 mg/L) and mortality........ 52
Figure S93. Forest plot of association between increased D-dimer (>1.0 μg/mL) and mortality........ 52

Other Predictors

Figure S94. Forest plot of association between respiratory rate ≥30 breaths per min and mortality........ 53
Figure S95. Forest plot of association between respiratory rate ≥24 breaths per min and mortality........ 53
Figure S96. Forest plot of association between bilateral pneumonia and mortality........ 53
Figure S97. Forest plot of association between highest temperature ≥39 ℃ and mortality 53
Figure S98. Forest plot of mean difference in respiratory rate between nonsurvivors and survivors........ 53
Figure S99. Forest plot of mean difference in heart rate between nonsurvivors and survivors........ 54
Figure S100. Forest plot of mean difference in PaO_2 between nonsurvivors and survivors 54
Figure S101. Forest plot of mean difference in $PaCO_2$ between nonsurvivors and survivors........ 54
Figure S102. Forest plot of mean difference in SpO_2 between nonsurvivors and survivors 54
Figure S103. Forest plot of mean difference in PaO2/FiO2 between nonsurvivors and survivors........ 54
Figure S104. Forest plot of mean difference in APACHE II score between nonsurvivors and survivors........ 55
Figure S105. Forest plot of mean difference in SOFA score between nonsurvivors and survivors 55
Figure S106. Forest plot of mean difference in time from illness onset to hospital admission between nonsurvivors and survivors 55

Notes and Abbreviations

Notes:

The study by Guan WJ et al. included 1590 patients from Renmin Hospital of Wuhan University, Jinyintan Hospital, Union Hospital, Central Hospital of Wuhan, Wuhan Pulmonary Hospital and other 570 hospitals in China. The study by Guan WJ et al. and the studies from the above-mentioned hospitals (e.g. the studies by Du RH et al. and Zhou F et al.) contained duplicate patients. To avoid potential patient overlap, we extracted the data only from the study by Guan WJ et al. (the larger study) for analyses if multidata were available.

Abbreviations:

ALT, alanine aminotransferase; APACHE II, Acute Physiology and Chronic Health Evaluation II; APTT, activated partial thromboplastin time; AST, aspartate aminotransferase; ESR, erythrocyte sedimentation rate; ECMO, extracorporeal membrane oxygenation; hs-cTnI, hypersensitive cardiac troponin I; $PaCO_2$, partial pressure of carbon dioxide; PaO_2, partial pressure of oxygen; PaO_2/FiO_2, ratio of partial pressure of oxygen to fraction of inspired oxygen; NT-proBNP, N-terminal pro-brain natriuretic peptide; SOFA, Sequential Organ Failure Assessment; SpO_2, peripheral oxygen saturation.

a: The number of persons exposed to risk factors in nonsurvivor group.
b: The number of persons unexposed to risk factors in nonsurvivor group.
c: The number of persons exposed to risk factors in survivor group.
d: The number of persons unexposed to risk factors in survivor group.

Table S1. Quality Assessments of the Included Studies

Study	Study participation	Study attrition	Prognostic factor measurement	Outcome measurement	Study confounding	Statistical analysis and reporting
Cao J et al.	Low risk	Low risk	Moderate risk	Low risk	High risk	Moderate risk
Chen R et al.	Moderate risk	Low risk	Moderate risk	Low risk	Low risk	Low risk
Chen T et al. (a)	Low risk	Low risk	Moderate risk	Low risk	Low risk	Low risk
Chen T et al. (b)	Low risk	Low risk	Moderate risk	Low risk	High risk	Moderate risk
Cheng Y et al.	Low risk	Low risk	Moderate risk	Low risk	Low risk	Low risk
Deng Y et al.	Low risk	Low risk	Moderate risk	Low risk	High risk	Moderate risk
Du RH et al.	Low risk	Low risk	Moderate risk	Low risk	Low risk	Low risk
Gao L et al.	High risk	High risk	Moderate risk	Low risk	Low risk	Low risk
Grasselli L et al.	Low risk	Low risk	Low risk	Low risk	High risk	Moderate risk
Guan WJ et al.	Low risk	Low risk	Moderate risk	Low risk	Low risk	Low risk
Guo T et al.	Low risk	Low risk	Low risk	Low risk	High risk	Moderate risk
He XW et al.	Low risk	Low risk	Low risk	Low risk	High risk	Moderate risk
Hu H et al.	Low risk	Low risk	Moderate risk	Low risk	High risk	Moderate risk
Li J et al.	High risk	High risk	Low risk	Low risk	High risk	Moderate risk
Li X et al.	Low risk	Low risk	Moderate risk	Low risk	Low risk	Low risk
Liang WH et al.	Low risk	Low risk	Moderate risk	Low risk	Low risk	Low risk
Liu Y et al. (a)	Low risk	Low risk	Low risk	Low risk	Low risk	Low risk
Liu Y et al. (b)	Low risk	Low risk	Low risk	Low risk	High risk	Moderate risk
Luo M et al.	Moderate risk	Low risk	Moderate risk	Low risk	Low risk	Low risk
Miyashita H et al.	Moderate risk	Low risk	Moderate risk	Low risk	High risk	Moderate risk
Peng YD et al.	Low risk	Low risk	Moderate risk	Low risk	High risk	Moderate risk
Richardson S et al.	Low risk	Low risk	Low risk	Low risk	High risk	Moderate risk
Shi S et al.	Low risk	Low risk	Low risk	Low risk	High risk	Moderate risk
Tang N et al. (a)	Low risk	Low risk	Low risk	Low risk	Low risk	Low risk
Tang N et al. (b)	Low risk	Low risk	Low risk	Low risk	High risk	Moderate risk
Wang L et al. (a)	Low risk	Low risk	Low risk	Low risk	Low risk	Low risk
Wang L et al. (b)	Low risk	Low risk	Moderate risk	Low risk	Low risk	Low risk
Wu C et al.	Low risk	Low risk	Moderate risk	Low risk	High risk	Moderate risk
Xie J et al.	Low risk	Low risk	Moderate risk	Low risk	Low risk	Low risk
Xu B et al.	Low risk	Low risk	Moderate risk	Low risk	High risk	Moderate risk
Yang X et al. (a)	Moderate risk	Low risk	Moderate risk	Low risk	High risk	Moderate risk
Yang X et al. (b)	Low risk	Low risk	Moderate risk	Low risk	High risk	Moderate risk
Yao Q et al.	Low risk	Low risk	Moderate risk	Low risk	High risk	Moderate risk
Zhang J et al.	Low risk	Low risk	Moderate risk	Low risk	High risk	Moderate risk
Zhang L et al.	Low risk	Low risk	Moderate risk	Low risk	Low risk	Low risk
Zhou F et al.	Low risk	Low risk	Moderate risk	Low risk	Low risk	Low risk

Table S2. Data of Age between Nonsurvivors and Survivors

Study	Nonsurvivors			Survivors			Setting
	Mean	SD	Total	Mean	SD	Total	
Cao J et al.	72	14.55	17	55.33	14.33	85	Zhongnan Hospital
Du RH et al.	70.2	7.7	21	56	13.5	158	Wuhan Pulmonary Hospital
Hu H et al.	75.05	12.94	19	57.71	15.34	86	Renmin Hospital of Wuhan University (2020 Feb 7-Mar 7)
Tang N et al. (a)	68.7	11.4	134	63.7	12.2	315	Tongji Hospital
Wu C et al.	67.6	12.03	44	47.33	10.51	117	Wuhan Jinyintan Hospital
Xu B et al.	72.75	7.23	28	55	17.26	117	Hubei Provincial Hospital of traditional Chinese and Western medicine
Yao Q et al.	63.17	18.87	12	56.67	10.8	13	Huanggang Central Hospital
Zhang J et al.	68.7	13.36	25	56.7	18.57	638	Renmin Hospital of Wuhan University (2020 Jan 11-Feb 6)

Table S3. Data of Association between Advanced Age and Mortality

Study	a	b	c	d	Setting	Definition
Chen T et al. (a)	94	19	59	102	Tongji Hospital	≥60 years
Chen T et al. (b)	19	7	36	141	Zhongnan Hospital	≥65 years
Du RH et al.	17	4	48	110	Wuhan Pulmonary Hospital	≥65 years
Grasselli L et al.	283	119	503	676	ICUs in 72 hospitals	≥64 years
Luo M et al.	32	37	48	358	Wuhan Tongren Hospital	≥70 years
Miyashita H et al.	371	184	1588	3545	Mount Sinai Health System in New York City	>65 years
Richardson S et al.	419	134	676	1405	A total of 12 hospitals in the USA	>65 years
Yang X et al. (b)	20	12	7	13	Wuhan Jin Yintan hospital	≥60 years
Yao Q et al.	6	6	11	85	Huanggang Central Hospital	>65 years
Zhang J et al.	19	6	296	342	Renmin Hospital of Wuhan University	>60 years

Table S4. Data of Association between Male Sex and Mortality

Study	a	b	c	d	Setting
Du RH et al.	10	11	87	71	Wuhan Pulmonary Hospital
Hu H et al.	14	5	48	38	Renmin Hospital of Wuhan University (2020 Feb 7-Mar 7)
Li J et al.	40	24	257	337	Central Hospital of Wuhan
Liu Y et al. (a)	25	8	89	123	Zhongnan Hospital
Luo M et al.	46	23	185	221	Wuhan Tongren Hospital
Richardson S et al.	337	216	1162	919	A total of 12 hospitals in the USA
Tang N et al. (a)	90	44	178	137	Tongji Hospital
Xu B et al.	17	11	59	58	Hubei Provincial Hospital of Traditional Chinese and Western Medicine
Yang X et al. (a)	156	82	620	618	Wuhan Jinyintan Hospital
Yao Q et al.	7	5	36	60	Huanggang Central Hospital
Zhang J et al.	15	10	306	332	Renmin Hospital of Wuhan University (2020 Jan 11-Feb 6)

Table S5. Data of Association between Current Smoking and Mortality

Study	a	b	c	d	Setting
Chen T et al. (a)	7	106	5	156	Tongji Hospital
Liu Y et al. (a)	3	30	7	205	Zhongnan Hospital
Yao Q et al.	3	9	1	95	Huanggang Central Hospital
Zhou F et al.	5	49	6	131	Jinyintan Hospital and Wuhan Pulmonary Hospital

Table S6. Data of Association between Preexisting Any Comorbidity and Mortality

Study	a	b	c	d	Setting
Cao J et al.	13	4	34	51	Zhongnan Hospital
Guan WJ et al.	35	15	364	1176	A total of 575 hospitals in China
Luo M et al.	45	24	15	391	Wuhan Tongren Hospital
Tang N et al. (a)	91	43	181	134	Tongji Hospital
Xu B et al.	21	7	47	70	Hubei Provincial Hospital of Traditional Chinese and Western Medicine

Table S7. Data of Association between Preexisting Chronic Kidney Disease and Mortality

Study	a	b	c	d	Setting
Cao J et al.	3	14	1	84	Zhongnan Hospital
Chen T et al. (a)	4	109	1	160	Tongji Hospital
Guan WJ et al.	5	45	16	1524	A total of 575 hospitals in China
Xu B et al.	2	26	2	115	Hubei Provincial Hospital of Traditional Chinese and Western Medicine

Table S8. Data of Association between Cerebrovascular Disease and Mortality

Study	a	b	c	d	Setting
Cao J et al.	3	14	3	82	Zhongnan Hospital
Chen T et al. (a)	4	109	0	161	Tongji Hospital
Guan WJ et al.	6	44	24	1516	A total of 575 hospitals in China (2019 Dec 11-2020 Jan 31)
Hu H et al.	3	16	1	85	Renmin Hospital of Wuhan University (2020 Feb 7-Mar 7)

Table S9. Data of Association between Preexisting Chronic Respiratory Disease and Mortality

Study	a	b	c	d	Setting
Chen T et al. (a)	11	102	7	154	Tongji Hospital
Guan WJ et al.	6	44	18	1522	A total of 575 hospitals in China (2019 Dec 11-2020 Jan 31)
Hu H et al.	5	14	7	79	Renmin Hospital of Wuhan University (2020 Feb 7-Mar 7)
Liu Y et al. (a)	3	30	5	207	Zhongnan Hospital
Xu B et al.	0	28	2	115	Hubei Provincial Hospital of Traditional Chinese and Western Medicine

Table S10. Data of Association between Preexisting Cardiovascular Disease and Mortality

Study	a	b	c	d	Setting
Cao J et al.	3	14	2	83	Zhongnan Hospital
Chen T et al. (a)	16	97	7	154	Tongji Hospital
Guan WJ et al.	8	42	51	1489	A total of 575 hospitals in China (2019 Dec 11-2020 Jan 31)
Guo T et al.	29	14	37	107	Seventh Hospital of Wuhan City
Hu H et al.	1	18	5	81	Renmin Hospital of Wuhan University (2020 Feb 7-Mar 7)

Table S11. Data of Association between Preexisting Diabetes Mellitus and Mortality

Study	a	b	c	d	Setting
Chen T et al. (a)	24	89	23	138	Tongji Hospital
Guan WJ et al.	13	37	117	1423	A total of 575 Hospitals in China (2019 Dec 11-2020 Jan 31)
Hu H et al.	0	19	4	82	Renmin Hospital of Wuhan University (2020 Feb 7-Mar 7)
Liu Y et al. (a)	7	26	16	196	Zhongnan Hospital
Luo M et al.	4	65	13	393	Wuhan Tongren Hospital

Table S12. Data of Association between Preexisting Hypertension and Mortality

Study	a	b	c	d	Setting
Chen T et al. (a)	54	59	39	122	Tongji Hospital
Grasselli L et al.	195	114	84	128	ICUs in 72 hospitals
Guan WJ et al.	28	22	241	1299	A total of 575 Hospitals in China (2019 Dec 11-2020 Jan 31)
Hu H et al.	6	13	22	64	Renmin Hospital of Wuhan University (2020 Feb 7-Mar 7)
Liu Y et al. (a)	15	18	37	175	Zhongnan Hospital
Xu B et al.	10	18	21	96	Hubei Provincial Hospital of Traditional Chinese and Western Medicine

Table S13. Data of Association between Preexisting Malignancy and Mortality

Study	a	b	c	d	Setting
Chen T et al. (a)	5	108	2	159	Tongji Hospital
Guan WJ et al.	3	47	15	1525	A total of 575 Hospitals in China (2019 Dec 11-2020 Jan 31)
Hu H et al.	1	18	5	81	Renmin Hospital of Wuhan University (2020 Feb 7-Mar 7)
Liu Y et al. (a)	2	31	7	205	Zhongnan Hospital
Miyashita H et al.	37	518	297	4836	Mount Sinai Health System in New York City

Table S14. Data of Association between Preexisting Gastrointestinal Disease and Mortality

Study	a	b	c	d	Setting
Chen T et al. (a)	1	112	2	159	Tongji Hospital
Du RH et al.	4	17	17	141	Wuhan Pulmonary Hospital
Zhang J et al.	1	24	30	608	Renmin Hospital of Wuhan University

Table S15. Data of Association between Preexisting Chronic Liver Disease and Mortality

Study	a	b	c	d	Setting	Definition
Chen T et al. (a)	5	108	6	155	Tongji Hospital	Hepatitis B virus surface antigen positivity
Guan WJ et al.	1	49	27	1513	A total of 575 hospitals in China	Hepatitis B infection
Liu Y et al. (a)	2	31	5	207	Zhongnan Hospital	Chronic liver Disease

Table S16. Data of Association between Preexisting Autoimmune Disease and Mortality

Study	a	b	c	d	Setting
Chen T et al. (a)	1	112	1	160	Tongji Hospital
Wang L et al. (b)	1	64	4	270	Renmin Hospital of Wuhan University

Table S17. Data of Association between Symptoms of Fever and Mortality

Study	a	b	c	d	Setting
Cao J et al.	12	5	61	24	Zhongnan Hospital
Chen T et al. (a)	104	9	145	16	Tongji Hospital
Du RH et al.	21	0	156	2	Wuhan Pulmonary Hospital
Wu C et al.	39	5	149	8	Wuhan Jinyintan Hospital
Xu B et al.	23	5	96	21	Hubei Provincial Hospital of Traditional Chinese and Western Medicine
Yao Q et al.	8	4	72	24	Huanggang Central Hospital
Zhang J et al.	19	6	508	130	Renmin Hospital of Wuhan University

Table S18. Data of Association between Symptoms of Cough and Mortality

Study	a	b	c	d	Setting
Cao J et al.	8	9	42	43	Zhongnan Hospital
Chen T et al. (a)	79	34	106	55	Tongji Hospital
Du RH et al.	14	7	132	26	Wuhan Pulmonary Hospital
Wu C et al.	33	11	130	27	Wuhan Jinyintan Hospital
Xu B et al.	23	5	89	28	Hubei Provincial Hospital of Traditional Chinese and Western Medicine
Yao Q et al.	10	2	74	22	Huanggang Central Hospital
Zhang J et al.	16	9	394	244	Renmin Hospital of Wuhan University

Table S19. Data of Association between Symptoms of Dyspnea and Mortality

Study	a	b	c	d	Setting
Chen T et al. (a)	70	43	50	111	Tongji Hospital
Du RH et al.	18	3	71	87	Wuhan Pulmonary Hospital
Wu C et al.	29	15	51	106	Wuhan Jinyintan Hospital
Xie J et al.	26	10	43	61	Union Hospital
Yao Q et al.	6	6	9	87	Huanggang Central Hospital
Zhang J et al.	11	14	150	488	Renmin Hospital of Wuhan University

Table S20. Data of Association between Symptoms of Chest Tightness and Mortality

Study	a	b	c	d	Setting
Chen T et al. (a)	55	58	48	113	Tongji Hospital
Zhang J et al.	6	19	148	490	Renmin Hospital of Wuhan University

Table S21. Data of Association between Symptoms of Sputum Production and Mortality

Study	a	b	c	d	Setting
Chen T et al. (a)	35	78	48	113	Tongji Hospital
Du RH et al.	12	9	43	115	Wuhan Pulmonary Hospital
Wu C et al.	19	25	64	93	Wuhan Jinyintan Hospital
Yao Q et al.	5	7	29	67	Huanggang Central Hospital
Zhang J et al.	9	16	157	481	Renmin Hospital of Wuhan University

Table S22. Data of Association between Symptoms of Fatigue and Mortality

Study	a	b	c	d	Setting
Cao J et al.	9	8	47	38	Zhongnan Hospital
Chen T et al. (a)	64	49	73	88	Tongji Hospital
Zhang J et al.	9	16	199	439	Renmin Hospital of Wuhan University
Zhou F et al.	15	39	29	108	Jinyintan Hospital and Wuhan Pulmonary Hospital

Table S23. Data of Association between Symptoms of Anorexia and Mortality

Study	a	b	c	d	Setting
Chen T et al. (a)	31	82	35	126	Tongji Hospital
Wang L et al. (b)	15	50	79	195	Renmin Hospital of Wuhan University
Xu B et al.	27	1	91	26	Hubei Provincial Hospital of Traditional Chinese and Western Medicine

Table S24. Data of Association between Symptoms of Diarrhea and Mortality

Study	a	b	c	d	Setting
Cao J et al.	3	14	8	77	Zhongnan Hospital
Chen T et al. (a)	27	86	50	111	Tongji Hospital
Xu B et al.	6	22	28	89	Hubei Provincial Hospital of Traditional Chinese and Western Medicine
Yao Q et al.	1	11	7	89	Huanggang Central Hospital
Zhang J et al.	0	25	61	577	Renmin Hospital of Wuhan University
Zhou F et al.	2	52	7	130	Jinyintan Hospital and Wuhan Pulmonary Hospital

Table S25. Data of Association between Symptoms of Myalgia and Mortality

Study	a	b	c	d	Setting
Cao J et al.	5	12	30	55	Zhongnan Hospital
Chen T et al. (a)	21	92	39	122	Tongji Hospital
Xu B et al.	3	25	28	89	Hubei Provincial Hospital of Traditional Chinese and Western Medicine
Zhang J et al.	0	25	63	575	Renmin Hospital of Wuhan University
Zhou F et al.	8	46	21	116	Jinyintan Hospital and Wuhan Pulmonary Hospital

Table S26. Data of Association between Symptoms of Dizziness and Mortality

Study	a	b	c	d	Setting
Chen T et al. (a)	10	103	11	150	Tongji Hospital
Xu B et al.	5	23	10	107	Hubei Provincial Hospital of Traditional Chinese and Western Medicine
Zhang J et al.	1	24	22	616	Renmin Hospital of Wuhan University

Table S27. Data of Association between Symptoms of Nausea and Mortality

Study	a	b	c	d	Setting
Chen T et al. (a)	8	105	16	145	Tongji Hospital
Xu B et al.	7	21	11	106	Hubei Provincial Hospital of Traditional Chinese and Western Medicine
Zhang J et al.	1	24	30	608	Renmin Hospital of Wuhan University

Table S28. Data of Association between Symptoms of Headache and Mortality

Study	a	b	c	d	Setting
Chen T et al. (a)	11	102	20	141	Tongji Hospital
Du RH et al.	5	16	12	146	Wuhan Pulmonary Hospital
Xu B et al.	1	27	5	112	Hubei Provincial Hospital of Traditional Chinese and Western Medicine
Yao Q et al.	0	12	1	95	Huanggang Central Hospital
Zhang J et al.	0	25	20	618	Renmin Hospital of Wuhan University

Table S29. Data of Association between Symptoms of Vomiting and Mortality

Study	a	b	c	d	Setting
Chen T et al. (a)	6	107	10	151	Tongji Hospital
Yang X et al. (b)	1	31	1	19	Wuhan Jin Yintan hospital
Zhang J et al.	1	24	16	622	Renmin Hospital of Wuhan University

Table S30. Data of Association between Symptoms of Pharyngalgia and Mortality

Study	a	b	c	d	Setting
Chen T et al. (a)	4	109	8	153	Tongji Hospital
Wang L et al. (b)	3	62	10	264	Renmin Hospital of Wuhan University
Xu B et al.	2	26	6	111	Hubei Provincial Hospital of Traditional Chinese and Western Medicine

Table S31. Data of Association between Symptoms of Hemoptysis and Mortality

Study	a	b	c	d	Setting
Chen T et al. (a)	4	109	3	158	Tongji Hospital
Du RH et al.	0	21	10	148	Wuhan Pulmonary Hospital

Table S32. Data of Association between Shock and Mortality

Study	a	b	c	d	Setting
Cao J et al.	7	10	3	82	Zhongnan Hospital
Chen T et al. (a)	46	67	0	161	Tongji Hospital
Wang L et al. (b)	3	62	5	269	Renmin Hospital of Wuhan University

Table S33. Data of Association between Acute Infection and Mortality

Study	a	b	c	d	Setting	Note
Cao J et al.	14	3	3	82	Zhongnan Hospital	Acute infection
Wang L et al. (b)	49	16	94	180	Renmin Hospital of Wuhan University	Bacterial Infection
Xu B et al.	11	17	9	108	Hubei Provincial Hospital of Traditional Chinese and Western Medicine	Bacterial infection
Zhou F et al.	27	27	1	136	Jinyintan Hospital and Wuhan Pulmonary Hospital	Secondary infection

Table S34. Data of Association between Acute Kidney Injury and Mortality

Study	a	b	c	d	Setting
Cao J et al.	15	2	5	80	Zhongnan Hospital
Chen T et al. (a)	28	85	1	160	Tongji Hospital
Richardson S et al.	347	134	176	1693	A total of 12 hospitals in the USA
Wang L et al. (b)	17	48	11	263	Renmin Hospital of Wuhan University
Yao Q et al.	7	5	9	87	Huanggang Central Hospital
Zhou F et al.	27	27	1	136	Jinyintan Hospital and Wuhan Pulmonary Hospital

Table S35. Data of Association between Acute Cardiac Injury and Mortality

Study	a	b	c	d	Setting
Cao J et al.	12	5	3	82	Zhongnan Hospital
Chen T et al. (a)	72	22	18	91	Tongji Hospital
Guo T et al.	31	12	21	123	Seventh Hospital of Wuhan City
Shi S et al.	42	15	40	319	Renmin Hospital of Wuhan University
Yao Q et al.	6	6	2	94	Huanggang Central Hospital
Zhou F et al.	32	22	1	136	Jinyintan Hospital and Wuhan Pulmonary Hospital

Table S36. Data of Association between Acute Respiratory Distress Syndrome and Mortality

Study	a	b	c	d	Setting
Cao J et al.	15	2	5	80	Zhongnan Hospital
Chen T et al. (a)	113	0	83	78	Tongji Hospital
Wang L et al. (b)	56	9	15	259	Renmin Hospital of Wuhan University
Yao Q et al.	12	0	33	63	Huanggang Central Hospital
Zhou F et al.	50	4	9	128	Jinyintan Hospital and Wuhan Pulmonary Hospital

Table S37. Data of Association between Arrhythmia and Mortality

Study	a	b	c	d	Setting
Cao J et al.	12	5	6	79	Zhongnan Hospital
Wang L et al. (b)	13	52	22	252	Renmin Hospital of Wuhan University

Table S38. Data of Association between Heart Failure and Mortality

Study	a	b	c	d	Setting
Chen et al (a)	41	42	3	91	Tongji Hospital
Peng YD et al.	13	4	27	68	Union Hospital
Wang L et al. (b)	25	40	33	241	Renmin Hospital of Wuhan University
Zhou F et al.	28	26	16	121	Jinyintan Hospital and Wuhan Pulmonary Hospital

Table S39 Data of Association between Acute Liver Injury and Mortality

Study	a	b	c	d	Setting
Cao J et al.	13	4	21	64	Zhongnan Hospital
Chen T et al. (a)	10	103	3	158	Tongji Hospital
Richardson S et al.	53	498	3	2043	A total of 12 hospitals in the USA
Wang L et al. (b)	22	43	74	200	Renmin Hospital of Wuhan University
Yang X et al. (b)	9	23	6	14	Wuhan Jin Yintan hospital

Table S40. Data of Association between Sepsis and Mortality

Study	a	b	c	d	Setting
Chen T et al. (a)	113	0	66	95	Tongji Hospital
Yao Q et al.	11	1	24	72	Huanggang Central Hospital
Zhou F et al.	54	0	58	79	Jinyintan Hospital and Wuhan Pulmonary Hospital

Table S41. Data of Association between Renal Replacement Therapy and Mortality

Study	a	b	c	d	Setting
Cao J et al.	5	12	1	84	Zhongnan Hospital
Chen T et al. (a)	3	110	0	161	Tongji Hospital
Richardson S et al.	78	475	3	2078	A total of 12 hospitals in the USA
Yao Q et al.	1	11	0	96	Huanggang Central Hospital
Zhou F et al.	10	44	0	137	Jinyintan Hospital and Wuhan Pulmonary Hospital

Table S42. Data of Association between Invasive Mechanical Ventilation and Mortality

Study	a	b	c	d	Setting
Cao J et al.	12	5	2	83	Zhongnan Hospital
Chen T et al. (a)	17	96	0	161	Tongji Hospital
Richardson S et al.	282	271	38	2043	A total of 12 hospitals in the USA
Wu C et al.	5	39	0	157	Wuhan Jinyintan Hospital
Xu B et al.	9	19	0	117	Hubei Provincial Hospital of Traditional Chinese and Western Medicine
Yao Q et al.	10	2	0	96	Huanggang Central Hospital

Table S43. Data of Association between Noninvasive Ventilation and Mortality

Study	a	b	c	d	Setting
Cao J et al.	3	14	2	83	Zhongnan Hospital
Chen T et al. (a)	76	37	26	135	Tongji Hospital
Wu C et al.	38	6	23	134	Wuhan Jinyintan Hospital
Xu B et al.	13	15	9	108	Hubei Provincial Hospital of Traditional Chinese and Western Medicine
Yao Q et al.	2	10	2	94	Huanggang Central Hospital

Table S44. Data of Association between High Flow Nasal Cannula and Mortality

Study	a	b	c	d	Setting
Chen T et al. (a)	77	36	8	153	Tongji Hospital
Xu B et al.	2	26	1	116	Hubei Provincial Hospital of Traditional Chinese and Western Medicine
Yao et al	0	12	4	92	Huanggang Central Hospital
Zhou F et al.	33	21	8	129	Jinyintan Hospital and Wuhan Pulmonary Hospital

Table S45. Data of Association between ECMO and Mortality

Study	a	b	c	d	Setting
Cao J et al.	1	16	2	83	Zhongnan Hospital
Chen T et al. (a)	1	112	0	161	Tongji Hospital
Wu C et al.	1	43	0	157	Wuhan Jinyintan Hospital

Table S46. Data of Association between Corticosteroid Therapy and Mortality

Study	a	b	c	d	Setting
Cao J et al.	11	6	40	45	Zhongnan Hospital
Chen T et al. (a)	99	14	118	43	Tongji Hospital
Wu C et al.	23	21	39	118	Wuhan Jinyintan Hospital
Xu B et al.	24	4	63	54	Hubei Provincial Hospital of Traditional Chinese and Western Medicine
Yao Q et al.	10	2	20	76	Huanggang Central Hospital

Table S47. Data of Association between Antibiotic Therapy and Mortality

Study	a	b	c	d	Setting
Cao J et al.	17	0	84	1	Zhongnan Hospital
Chen T et al. (a)	105	8	144	17	Tongji Hospital
Wu C et al.	43	1	153	4	Wuhan Jinyintan Hospital
Xu B et al.	28	0	92	25	Hubei Provincial Hospital of Traditional Chinese and Western Medicine
Yao et al	12	0	36	60	Huanggang Central Hospital

Table S48. Data of Association between Immunoglobulin Therapy and Mortality

Study	a	b	c	d	Setting
Cao J et al.	0	17	5	80	Zhongnan Hospital
Chen T et al. (a)	44	69	59	102	Tongji Hospital
Xu B et al.	15	13	54	63	Hubei Provincial Hospital of Traditional Chinese and Western Medicine
Yao et al	3	9	9	87	Huanggang Central Hospital
Zhou F et al.	36	18	10	127	Jinyintan Hospital and Wuhan Pulmonary Hospital

Table S49. Data of Association between Antiviral Therapy and Mortality

Study	a	b	c	d	Setting
Cao J et al.	17	0	83	2	Zhongnan Hospital
Chen T et al. (a)	89	24	147	14	Tongji Hospital
Wu C et al.	25	19	145	12	Wuhan Jinyintan Hospital
Yao Q et al.	12	0	96	0	Huanggang Central Hospital

Table S50. Data of White Blood Cell Count between Nonsurvivors and Survivors

Study	Nonsurvivors			Survivors			Setting
	Mean	SD	Total	Mean	SD	Total	
Chen T et al. (a)	10	5.56	113	5	1.94	161	Tongji Hospital
Chen T et al. (b)	11.7	17.62	19	7.2	9.26	36	Zhongnan Hospital
Du RH et al.	8.93	6.6	21	5.4	2.62	158	Wuhan Pulmonary Hospital
Wang L et al. (b)	9.04	6.05	65	5.82	2.5	274	Renmin Hospital of Wuhan University
Wu C et al.	8.65	4.25	44	5.19	2.86	117	Wuhan Jinyintan Hospital
Xu B et al.	7.16	4.18	28	5.34	2.44	117	Hubei Provincial Hospital of Traditional Chinese and Western Medicine
Yao Q et al.	9.67	4.47	12	5.91	5.12	13	Huanggang Central Hospital

Table S51. Data of Neutrophil Count between Nonsurvivors and Survivors

Study	Nonsurvivors			Survivors			Setting
	Mean	SD	Total	Mean	SD	Total	
Chen T et al. (a)	9.03	5.48	113	3.37	1.57	161	Tongji Hospital
Chen T et al. (b)	9.5	15.22	19	6.1	9.26	36	Zhongnan Hospital
Du RH et al.	7.4	6.76	21	4.2	2.62	158	Wuhan Pulmonary Hospital
Wang L et al. (b)	7.91	5.6	65	4.2	2.49	274	Renmin Hospital of Wuhan University
Wu C et al.	7.73	4.18	44	3.55	2.65	117	Wuhan Jinyintan Hospital
Xu B et al.	5.93	3.96	28	3.69	2.21	117	Hubei Provincial Hospital of Traditional Chinese and Western Medicine
Yao Q et al.	6.53	5.26	12	3.46	2.56	13	Huanggang Central Hospital

Table S52. Data of Lymphocyte Count between Nonsurvivors and Survivors

Study	Nonsurvivors			Survivors			Setting
	Mean	SD	Total	Mean	SD	Total	
Chen T et al. (a)	0.57	0.23	113	1.03	0.52	161	Tongji Hospital
Chen T et al. (b)	2.33	4.81	19	0.93	1.54	36	Zhongnan Hospital
Du RH et al.	0.67	0.24	21	0.83	0.37	158	Wuhan Pulmonary Hospital
Wang L et al. (b)	0.6	0.34	65	1.01	0.51	274	Renmin Hospital of Wuhan University
Wu C et al.	0.6	0.2	44	1.08	0.55	117	Wuhan Jinyintan Hospital
Xu B et al.	0.61	0.48	28	0.98	0.54	117	Hubei Provincial Hospital of Traditional Chinese and Western Medicine
Yao Q et al.	0.99	0.80	12	0.79	0.26	13	Huanggang Central Hospital

Table S53. Data of Monocyte Count between Nonsurvivors and Survivors

Study	Nonsurvivors			Survivors			Setting
	Mean	SD	Total	Mean	SD	Total	
Chen T et al. (a)	0.4	0.3	113	0.4	0.15	161	Tongji Hospital
Wang L et al. (b)	0.34	0.2	65	0.45	0.24	274	Renmin Hospital of Wuhan University
Wu C et al.	0.3	0.19	44	0.34	0.14	117	Wuhan Jinyintan Hospital
Xu B et al.	0.32	0.18	28	0.41	0.19	117	Hubei Provincial Hospital of Traditional Chinese and Western Medicine

Table S54. Data of Platelet Count between Nonsurvivors and Survivors

Study	Nonsurvivors			Survivors			Setting
	Mean	SD	Total	Mean	SD	Total	
Chen T et al. (b)	134.67	144.17	19	205.33	302.65	36	Zhongnan Hospital
Tang N et al. (a)	178	92	134	231	99	315	Tongji Hospital
Wang L et al. (b)	164.67	87.95	65	212.67	81.23	274	Renmin Hospital of Wuhan University
Wu C et al.	167.83	92.35	44	185.83	74.69	117	Wuhan Jinyintan Hospital
Yao Q et al.	165.33	52.83	12	149.67	68.11	13	Huanggang Central Hospital

Table S55. Data of Hemoglobin between Nonsurvivors and Survivors

Study	Nonsurvivors			Survivors			Setting
	Mean	SD	Total	Mean	SD	Total	
Chen T et al. (a)	129	23.28	113	128	14.96	161	Renmin Hospital
Wang L et al. (b)	121.67	23.5	65	120	14.16	274	Renmin Hospital of Wuhan University
Yao Q et al.	126.33	9.22	12	120.67	12.46	13	Huanggang Central Hospital
Zhou F et al.	126.33	17.52	54	129.33	14.98	137	Jinyintan Hospital and Wuhan Pulmonary Hospital

Table S56. Data of Albumin between Nonsurvivors and Survivors

Study	Nonsurvivors			Survivors			Setting
	Mean	SD	Total	Mean	SD	Total	
Chen T et al. (a)	30.33	3.83	113	36.5	4.34	161	Tongji Hospital
Chen T et al. (b)	32.67	12.01	19	34	13.13	36	Zhongnan Hospital
Du RH et al.	33.33	3.5	21	33.9	5.61	158	Wuhan Pulmonary Hospital
Wu C et al.	28.95	4.1	44	33.65	4.02	117	Wuhan Jinyintan Hospital
Yao Q et al.	31.07	5.03	12	37.17	3.99	13	Huanggang Central Hospital

Table S57. Data of Total Bilirubin between Nonsurvivors and Survivors

Study	Nonsurvivors			Survivors			Setting
	Mean	SD	Total	Mean	SD	Total	
Chen T et al. (a)	12.9	5.48	113	8.47	4.04	161	Tongji Hospital
Du RH et al.	11.4	6.36	21	9.17	4.34	158	Wuhan Pulmonary Hospital
Wu C et al.	14.88	7.24	44	10.92	3.79	117	Wuhan Jinyintan Hospital
Yao Q et al.	12.93	11.82	12	12.03	5.48	13	Huanggang Central Hospital

Table S58. Data of ALT between Nonsurvivors and Survivors

Study	Nonsurvivors			Survivors			Setting
	Mean	SD	Total	Mean	SD	Total	
Chen T et al. (a)	31	21.78	113	22.27	12.87	161	Tongji Hospital
Chen T et al. (b)	49.33	62.48	19	109	209.23	36	Zhongnan Hospital
Du RH et al.	28	13.52	21	25.5	19.83	158	Wuhan Pulmonary Hospital
Wang L et al. (b)	30.67	22.74	65	29.33	19.38	274	Renmin Hospital of Wuhan University
Wu C et al.	37.33	24.52	44	28.83	17.64	117	Wuhan Jinyintan Hospital
Xu B et al.	29.5	26.56	28	24.67	21.77	117	Hubei Provincial Hospital of Traditional Chinese and Western Medicine
Yao Q et al.	20.67	4.61	12	22.77	8.06	13	Huanggang Central Hospital

Table S59. Data of AST between Nonsurvivors and Survivors

Study	Nonsurvivors			Survivors			Setting
	Mean	SD	Total	Mean	SD	Total	
Chen T et al. (a)	47.67	27.03	113	26.1	9.95	161	Tongji Hospital
Chen T et al. (b)	88	120.15	19	94.67	145.92	36	Zhongnan Hospital
Du RH et al.	42.83	27.43	21	29.5	17.21	158	Wuhan Pulmonary Hospital
Wang L et al. (b)	47	28.81	65	31.33	15.65	274	Renmin Hospital of Wuhan University
Wu C et al.	39.67	16.86	44	30.83	10.88	117	Wuhan Jinyintan Hospital
Xu B et al.	40	24.61	28	25.67	12.76	117	Hubei Provincial Hospital of Traditional Chinese and Western Medicine

Table S60. Data of Creatinine between Nonsurvivors and Survivors

Study	Nonsurvivors			Survivors			Setting
	Mean	SD	Total	Mean	SD	Total	
Chen T et al. (a)	89.33	36.05	113	68	22.44	161	Tongji Hospital
Chen T et al. (b)	328	579.9	19	270.33	500.31	36	Zhongnan Hospital
Du RH et al.	90	38.96	21	66.67	18.7	158	Wuhan Pulmonary Hospital
Wang L et al. (b)	83	44.73	65	60	16.4	274	Renmin Hospital of Wuhan University
Wu C et al.	72.38	22.42	44	69.07	17.71	117	Wuhan Jinyintan Hospital
Xu B et al.	89.82	24.84	28	70.5	21.32	117	Hubei Provincial Hospital of Traditional Chinese and Western Medicine
Yao Q et al.	91.93	30.11	12	66.20	25.42	13	Huanggang Central Hospital

Table S61. Data of Blood Urea Nitrogen between Nonsurvivors and Survivors

Study	Nonsurvivors			Survivors			Setting
	Mean	SD	Total	Mean	SD	Total	
Chen T et al. (a)	8.90	5.18	113	4.03	1.57	161	Tongji Hospital
Xu B et al.	7.67	3.77	28	4.81	1.87	117	Hubei Provincial Hospital of Traditional Chinese and Western Medicine

Table S62. Data of Urea between Nonsurvivors and Survivors

Study	Nonsurvivors			Survivors			Setting
	Mean	SD	Total	Mean	SD	Total	
Wang L et al. (b)	9.7	6.22	65	5.33	2.31	274	Renmin Hospital of Wuhan University
Wu C et al.	7.08	2.8	44	4.37	1.5	117	Wuhan Jinyintan Hospital

Table S63. Data of Prothrombin Time between Nonsurvivors and Survivors

Study	Nonsurvivors			Survivors			Setting
	Mean	SD	Total	Mean	SD	Total	
Du RH et al.	14.17	3.18	21	13.77	2.09	158	Wuhan Pulmonary Hospital
Tang N et al. (a)	16.5	8.4	134	14.6	2.1	315	Tongji Hospital
Wang L et al. (b)	12.97	1.67	65	12.07	0.75	274	Renmin Hospital of Wuhan University
Wu C et al.	11.72	1.03	44	10.73	1.05	117	Wuhan Jinyintan Hospital
Xu B et al.	13.65	1.68	28	12.57	1.13	117	Hubei Provincial Hospital of Traditional Chinese and Western Medicine

Table S64. Data of APTT between Nonsurvivors and Survivors

Study	Nonsurvivors			Survivors			Setting
	Mean	SD	Total	Mean	SD	Total	
Chen T et al. (a)	41.03	8.49	113	40.63	5.31	161	Tongji Hospital
Du RH et al.	36.7	8.51	21	35.1	6.14	158	Wuhan Pulmonary Hospital
Wang L et al. (b)	29.43	3.34	65	28.37	4.17	274	Renmin Hospital of Wuhan University
Wu C et al.	24.9	4.67	44	29.38	5.48	117	Wuhan Jinyintan Hospital

Table S65. Data of C-reactive Protein between Nonsurvivors and Survivors

Study	Nonsurvivors			Survivors			Setting
	Mean	SD	Total	Mean	SD	Total	
Chen T et al. (a)	116.83	74.57	113	30.23	35.23	161	Tongji Hospital
Chen T et al. (b)	146.33	217.86	19	70.33	115.04	36	Zhongnan Hospital
Du RH et al.	76.6	53.74	21	48.77	53.64	158	Wuhan Pulmonary Hospital
Wang L et al. (b)	116.1	97.42	65	46.63	51.2	274	Renmin Hospital of Wuhan University
Wu C et al.	98.47	88.48	44	29.28	38.39	117	Wuhan Jinyintan Hospital
Xu B et al.	88.65	54.54	28	24.11	35.53	117	Hubei Provincial Hospital of Traditional Chinese and Western Medicine
Yao Q et al.	38.33	12.33	12	25.57	15.61	13	Huanggang Central Hospital

Table S66. Data of D-dimer between Nonsurvivors and Survivors

Study	Nonsurvivors			Survivors			Setting
	Mean	SD	Total	Mean	SD	Total	
Chen T et al. (b)	5.6	12.04	19	6.73	14.45	36	Zhongnan Hospital
Du RH et al.	4	8.03	21	0.67	0.67	158	Wuhan Pulmonary Hospital
Tang N et al. (a)	9.04	14.67	134	2.14	2.52	315	Tongji Hospital
Wang L et al. (b)	7.57	11.9	65	1.22	1.14	274	Renmin Hospital of Wuhan University
Wu C et al.	5.35	7.52	44	0.59	0.45	117	Wuhan Jinyintan Hospital
Xu B et al.	7.03	14.49	28	0.62	0.46	117	Hubei Provincial Hospital of Traditional Chinese and Western Medicine
Yao Q et al.	33.41	66.12	12	1.94	1.40	13	Huanggang Central Hospital

Table S67. Data of Procalcitonin between Nonsurvivors and Survivors

Study	Nonsurvivors			Survivors			Setting
	Mean	SD	Total	Mean	SD	Total	
Chen T et al. (a)	0.37	0.38	113	0.05	0.04	161	Tongji Hospital
Chen T et al. (b)	3.08	6.41	19	0.36	0.77	36	Zhongnan Hospital
Wang L et al. (b)	0.49	0.77	65	0.08	0.07	274	Renmin Hospital of Wuhan University
Xu B et al.	0.25	0.32	28	0.04	0.04	117	Hubei Provincial Hospital of Traditional Chinese and Western Medicine
Yao Q et al.	2.75	4.75	12	0.25	0.25	13	Huanggang Central Hospital
Zhou F et al.	0.23	0.30	54	0.1	0.001	137	Jinyintan Hospital and Wuhan Pulmonary Hospital

Table S68. Data of Ferritin between Nonsurvivors and Survivors

Study	Nonsurvivors			Survivors			Setting
	Mean	SD	Total	Mean	SD	Total	
Chen T et al. (a)	1.5233	0.99184	113	0.53927	0.45362	161	Tongji Hospital
Wu C et al.	1.21854	1.10402	44	0.46135	0.35948	117	Wuhan Jinyintan Hospital

Table S69. Data of Lactate Dehydrogenase between Nonsurvivors and Survivors

Study	Nonsurvivors			Survivors			Setting
	Mean	SD	Total	Mean	SD	Total	
Chen T et al. (a)	570.43	213.87	113	266.27	76.45	161	Tongji Hospital
Chen T et al. (b)	468.67	498.20	19	383.00	320.41	36	Zhongnan Hospital
Wang L et al. (b)	480.67	214.56	65	287	100.61	274	Renmin Hospital of Wuhan University
Wu C et al.	467.83	166.68	44	262.83	82.19	117	Wuhan Jinyintan Hospital

Table S70. Data of Creatine Kinase between Nonsurvivors and Survivors

Study	Nonsurvivors			Survivors			Setting
	Mean	SD	Total	Mean	SD	Total	
Chen T et al. (a)	219.33	210.26	113	91.7	66.95	161	Tongji Hospital
Chen T et al. (b)	406.33	751.31	19	125.67	189.16	36	Zhongnan Hospital
Wang L et al. (b)	118.67	130.4	65	60	16.4	274	Renmin Hospital of Wuhan University
Xu B et al.	142.33	65.62	28	86.17	67.18	117	Hubei Provincial Hospital of Traditional Chinese and Western Medicine
Yao Q et al.	97.70	52.16	12	106.17	85.96	13	Huanggang Central Hospital
Zhou F et al.	69.83	100.16	54	27.53	29.67	137	Jinyintan Hospital and Wuhan Pulmonary Hospital

Table S71. Data of γ-glutamyl Transpeptidase between Nonsurvivors and Survivors

Study	Nonsurvivors			Survivors			Setting
	Mean	SD	Total	Mean	SD	Total	
Chen T et al. (a)	46.33	32.29	113	30.77	19.67	161	Tongji Hospital
Du RH et al.	27.17	20.27	21	33.5	28.06	158	Wuhan Pulmonary Hospital

Table S72. Data of ESR between Nonsurvivors and Survivors

Study	Nonsurvivors			Survivors			Setting
	Mean	SD	Total	Mean	SD	Total	
Chen T et al. (a)	40.6	31.76	113	29.6	21.84	161	Tongji Hospital
Chen T et al. (b)	50	69.68	19	49.67	64.08	36	Zhongnan Hospital
Wu C et al.	57.17	25.29	44	50.67	18.24	117	Wuhan Jinyintan Hospital

Table S73. Data of Creatine Kinase-MB between Nonsurvivors and Survivors

Study	Nonsurvivors			Survivors			Setting
	Mean	SD	Total	Mean	SD	Total	
Wang L et al. (b)	2.85	2.27	65	1.29	0.82	274	Renmin Hospital of Wuhan University
Wu C et al.	16.67	5.36	44	15.33	5.25	117	Wuhan Jinyintan Hospital
Xu B et al.	20.83	10.55	28	13.67	6.00	117	Hubei Provincial Hospital of Traditional Chinese and Western Medicine
Yao Q et al.	22.57	11.32	12	13.83	4.57	13	Huanggang Central Hospital

Table S74. Data of NT-proBNP between Nonsurvivors and Survivors

Study	Nonsurvivors			Survivors			Setting
	Mean	SD	Total	Mean	SD	Total	
Chen T et al. (a)	1.00243	1.07212	113	0.09233	0.12343	161	Tongji Hospital
Wang L et al. (a)	0.86433	1.09733	33	0.15733	0.18918	169	Renmin Hospital of Wuhan University

Table S75. Data of hs-cTnI between Nonsurvivors and Survivors

Study	Nonsurvivors			Survivors			Setting
	Mean	SD	Total	Mean	SD	Total	
Chen T et al. (a)	71.10	107.46	113	4.07	3.82	161	Tongji Hospital
Wang L et al. (b)	144	237.3	65	10.33	8.94	274	Renmin Hospital of Wuhan University
Zhou F et al.	36.97	59.03	54	3.2	3.3	137	Jinyintan Hospital and Wuhan Pulmonary Hospital

Table S76. Data of Myoglobin between Nonsurvivors and Survivors

Study	Nonsurvivors			Survivors			Setting
	Mean	SD	Total	Mean	SD	Total	
Du RH et al.	184.43	233.98	21	36.03	33.52	158	Wuhan Pulmonary Hospital
Wang L et al. (a)	243.5	374.69	33	37.7	20.79	169	Renmin Hospital of Wuhan University

Table S77. Data of Cystatin C between Nonsurvivors and Survivors

Study	Nonsurvivors			Survivors			Setting
	Mean	SD	Total	Mean	SD	Total	
Wu C et al.	1.11	0.44	44	0.83	0.16	117	Wuhan Jinyintan Hospital
Yao Q et al.	2.55	2.98	12	1.53	0.51	13	Huanggang Central Hospital

Table S78. Data of Interleukin-6 between Nonsurvivors and Survivors

Study	Nonsurvivors			Survivors			Setting
	Mean	SD	Total	Mean	SD	Total	
Chen T et al. (a)	84.8	83.5	113	14.4	16.61	161	Tongji Hospital
Chen T et al. (b)	212.67	330.80	19	116	216.95	36	Zhongnan Hospital
Wang L et al. (b)	104	110.99	65	11.4	10.36	274	Renmin Hospital of Wuhan University
Wu C et al.	10.74	5.7	44	6.49	1.85	117	Wuhan Jinyintan Hospital
Xu B et al.	36.1	38.5	28	15.3	17.08	117	Hubei Provincial Hospital of Traditional Chinese and Western Medicine

Table S79. Data of CD3+ Cell Count between Nonsurvivors and Survivors

Study	Nonsurvivors			Survivors			Setting
	Mean	SD	Total	Mean	SD	Total	
Wu C et al.	311.33	173.20	44	648.67	284.48	117	Wuhan Jinyintan Hospital
Xu B et al.	163.33	106.63	28	462.33	262.71	117	Hubei Provincial Hospital of Traditional Chinese and Western Medicine

Table S80. Data of CD4+ Cell Count between Nonsurvivors and Survivors

Study	Nonsurvivors			Survivors			Setting
	Mean	SD	Total	Mean	SD	Total	
Wang L et al. (b)	193.33	132.68	65	360.67	222.83	274	Renmin Hospital of Wuhan University
Wu C et al.	202.33	141.01	44	408.67	216.93	117	Wuhan Jinyintan Hospital
Xu B et al.	97.42	68.94	28	249.67	131.36	117	Hubei Provincial Hospital of Traditional Chinese and Western Medicine

Table S81. Data of CD8+ Cell Count between Nonsurvivors and Survivors

Study	Nonsurvivors			Survivors			Setting
	Mean	SD	Total	Mean	SD	Total	
Wang L et al. (b)	91.67	89.46	65	199.67	149.8	274	Renmin Hospital of Wuhan University
Wu C et al.	102.17	58.63	44	241	123.1	117	Wuhan Jinyintan Hospital
Xu B et al.	70.33	56.24	28	171.33	122.35	117	Hubei Provincial Hospital of Traditional Chinese and Western Medicine

Table S82. Data of Association between Increased White Blood Cell Count (≥10 ×10^9/L) and Mortality

Study	a	b	c	d	Setting
Chen T et al. (a)	56	57	6	155	Tongji Hospital
Chen T et al. (b)	4	15	6	30	Zhongnan Hospital
Yao Q et al.	9	3	3	93	Dabieshan Medical Center
Zhou F et al.	25	29	15	122	Jinyintan Hospital and Wuhan Pulmonary Hospital

Table S83. Data of Association between Decreased Lymphocyte Count (<0.8 × 10^9/L) and Mortality

Study	a	b	c	d	Setting
Chen T et al. (a)	87	26	48	113	Tongji Hospital
Yao Q et al.	5	7	18	78	Dabieshan Medical Center
Zhou F et al.	41	13	36	101	Jinyintan Hospital and Wuhan Pulmonary Hospital

Table S84. Data of Association between Decreased Lymphocyte Count (<1.1 × 10^9/L) and Mortality

Study	a	b	c	d	Setting
Cao J et al.	11	6	54	31	Zhongnan Hospital
Du RH et al.	19	2	114	44	Wuhan Pulmonary Hospital

Table S85. Data of Association between Decreased Platelet Count (<100 × 10^9/L) and Mortality

Study	a	b	c	d	Setting
Chen T et al. (b)	5	14	4	32	Zhongnan Hospital
Yao Q et al.	1	11	9	87	Dabieshan Medical Center
Zhou F et al.	11	43	2	135	Jinyintan Hospital and Wuhan Pulmonary Hospital

Table S86. Data of Association between Decreased Platelet Count (<125 × 10^9/L) and Mortality

Study	a	b	c	d	Setting
Liu Y et al. (b)	21	28	47	287	Central Hospital of Wuhan
Yang X et al. (a)	173	65	133	1105	Jinyintan Hospital

Table S87. Data of Association between Increased ALT (>40 U/L) and Mortality

Study	a	b	c	d	Setting
Cao J et al.	7	10	18	66	Zhongnan Hospital
Chen T et al. (a)	30	83	30	131	Tongji Hospital
Zhou F et al.	26	28	33	102	Jinyintan Hospital and Wuhan Pulmonary Hospital

Table S88. Data of Association between Increased AST (>40 U/L) and Mortality

Study	a	b	c	d	Setting
Chen T et al. (a)	59	54	25	136	Tongji Hospital
Chen T et al. (b)	14	5	18	18	Zhongnan Hospital
Du RH et al.	10	11	47	111	Wuhan Pulmonary Hospital

Table S89. Data of Association between Increased Creatinine (>133 μmol/L) and Mortality

Study	a	b	c	d	Setting
Yao Q et al.	1	11	3	93	Dabieshan Medical Center
Zhou F et al.	5	49	3	129	Jinyintan Hospital and Wuhan Pulmonary Hospital

Table S90. Data of Association between Increased Procalcitonin (≥0.5 ng/mL) and Mortality

Study	a	b	c	d	Setting
Chen T et al. (a)	35	61	3	137	Tongji Hospital
Zhou F et al.	13	38	1	112	Jinyintan Hospital and Wuhan Pulmonary Hospital

Table S91. Data of Association between Increased Procalcitonin (≥0.1 ng/mL) and Mortality

Study	a	b	c	d	Setting
Cao J et al.	13	4	22	43	Zhongnan Hospital
Zhou F et al.	32	19	18	95	Jinyintan Hospital and Wuhan Pulmonary Hospital

Table S92. Data of Association between Increased Procalcitonin (≥0.05 ng/mL) and Mortality

Study	a	b	c	d	Setting
Chen T et al. (a)	95	1	70	70	Tongji Hospital
Yao Q et al.	9	3	52	44	Dabieshan Medical Center

Table S93. Data of Association between Increased C-reactive Protein (≥10 mg/L) and Mortality

Study	a	b	c	d	Setting
Cao J et al.	16	1	36	49	Zhongnan Hospital
Du RH et al.	20	1	138	20	Wuhan Pulmonary Hospital

Table S94. Data of Association between Increased D-dimer (>1.0 μg/mL) and Mortality

Study	a	b	c	d	Setting
Yao Q et al.	9	3	31	65	Huanggang Central Hospital
Zhou F et al.	44	10	28	90	Jinyintan Hospital and Wuhan Pulmonary Hospital

Table S95. Data of Association between Respiratory Rate ≥30 breaths per min and Mortality

Study	a	b	c	d	Setting
Chen T et al. (a)	30	83	5	156	Tongji Hospital
Liu Y et al. (a)	4	29	4	207	Zhongnan Hospital

Table S96. Data of Association between Respiratory Rate ≥24 breaths per min and Mortality

Study	a	b	c	d	Setting
Chen T et al. (a)	66	47	22	139	Tongji Hospital
Zhou F et al.	34	20	22	115	Jinyintan Hospital and Wuhan Pulmonary Hospital

Table S97. Data of Association between Bilateral Pneumonia and Mortality

Study	a	b	c	d	Setting
Cao J et al.	14	3	58	27	Zhongnan Hospital
Chen T et al. (a)	113	0	152	9	Tongji Hospital
Yao Q et al.	12	0	86	10	Huanggang Central Hospital
Zhang J et al.	20	5	530	108	Renmin Hospital of Wuhan University
Zhou F et al.	45	9	98	39	Jinyintan Hospital and Wuhan Pulmonary Hospital

Table S98. Data of Association between Highest Temperature ≥39 ℃ and Mortality

Study	a	b	c	d	Setting
Wu C et al.	14	30	63	94	Wuhan Jinyintan Hospital
Yao Q et al.	3	9	7	89	Huanggang Central Hospital

Table S99. Data of Respiratory Rate between Nonsurvivors and Survivors

Study	Nonsurvivors			Survivors			Setting
	Mean	SD	Total	Mean	SD	Total	
Chen T et al. (a)	24.67	7.51	113	20.33	0.75	161	Tongji Hospital
Du RH et al.	24.83	11.53	21	20.33	0.75	158	Wuhan Pulmonary Hospital
Hu H et al.	23.21	5.38	19	20.27	3.27	86	Renmin Hospital of Wuhan University (2020 Feb 7- 2020 Mar 7)
Wang L et al. (b)	23	9.1	65	19.33	1.49	274	Renmin Hospital of Wuhan University (2020 Jan 1- 2020 Feb 6)
Yao Q et al.	20.33	2.52	12	20.33	2.49	13	Huanggang Central Hospital

Table S100. Data of Heart Rate between Nonsurvivors and Survivors

Study	Nonsurvivors			Survivors			Setting
	Mean	SD	Total	Mean	SD	Total	
Chen T et al. (a)	98	21.78	113	91.33	18.70	161	Tongji Hospital
Du RH et al.	93.83	25.04	21	87.6	15.94	158	Wuhan Pulmonary Hospital
Hu H et al.	87.47	13.56	19	84.23	17.48	86	Renmin Hospital of Wuhan University (2020 Feb 7- Mar 7)
Wang L et al. (b)	87.67	17.44	65	82.67	10.43	274	Renmin Hospital of Wuhan University (2020 Jan 1- Feb 6)
Yang X et al. (b)	89	15	32	89	20	20	Wuhan Jin Yintan hospital
Yao Q et al.	81	12.58	12	84.33	5.81	13	Huanggang Central Hospital

Table S101. Data of PaO_2 between Nonsurvivors and Survivors

Study	Nonsurvivors			Survivors			Setting
	Mean	SD	Total	Mean	SD	Total	
Chen T et al. (a)	61.07	25.66	35	124.97	56.42	32	Tongji Hospital
Du RH et al.	58.67	17.49	21	75.17	24.69	158	Wuhan Pulmonary Hospital
Peng YD et al.	78.67	28.29	17	90	12.04	95	Union Hospital

Table S102. Data of $PaCO_2$ between Nonsurvivors and Survivors

Study	Nonsurvivors			Survivors			Setting
	Mean	SD	Total	Mean	SD	Total	
Chen T et al. (a)	31.93	5.49	35	37.93	4.89	32	Tongji Hospital
Du RH et al.	34.67	9.54	21	37.33	5.24	158	Wuhan Pulmonary Hospital
Peng YD et al.	37	8.89	17	41.67	4.52	95	Union Hospital

Table S103. Data of SpO_2 between Nonsurvivors and Survivors

Study	Nonsurvivors			Survivors			Setting
	Mean	SD	Total	Mean	SD	Total	
Chen T et al. (a)	84.67	14.27	113	96.77	2.02	161	Tongji Hospital
Hu H et al.	86.53	12.46	19	96.17	3.1	86	Renmin Hospital of Wuhan University
Peng YD et al.	95.33	3.23	17	96.33	2.26	95	Union Hospital

Table S104. Data of PaO_2/FiO_2 between Nonsurvivors and Survivors

Study	Nonsurvivors			Survivors			Setting
	Mean	SD	Total	Mean	SD	Total	
Chen T et al. (a)	117.13	71.50	35	329.67	151.33	32	Tongji Hospital
Du RH et al.	185.5	64.8	21	261.5	108.2	158	Wuhan Pulmonary Hospital
Peng YD et al.	215.67	253.02	17	429.33	25.59	95	Union Hospital
Yang X et al. (b)	62.8	17.15	32	99.03	47.95	20	Wuhan Jin Yintan hospital

Table S105. Data of APACHE II Score between Nonsurvivors and Survivors

Study	Nonsurvivors			Survivors			Setting
	Mean	SD	Total	Mean	SD	Total	
Yang X et al. (b)	18	3.1	32	14.33	3.99	20	Wuhan Jin Yintan hospital
Yao Q et al.	11.67	10.9	12	6	3.32	13	Huanggang Central Hospital

Table S106. Data of SOFA Score between Nonsurvivors and Survivors

Study	Nonsurvivors			Survivors			Setting
	Mean	SD	Total	Mean	SD	Total	
Yao Q et al.	4.33	5.03	12	2	1.66	13	Huanggang Central Hospital
Zhou F et al.	4.83	1.52	54	1.33	0.75	137	Jinyintan Hospital and Wuhan Pulmonary Hospital

Table S107 Data of Time from Illness Onset to Hospital Admission between Nonsurvivors and Survivors

Study	Nonsurvivors			Survivors			Setting
	Mean	SD	Total	Mean	SD	Total	
Cao J et al.	5.67	4.04	17	5.33	3.02	85	Zhongnan Hospital
Chen T et al. (a)	10	4.51	113	9	4.49	161	Tongji Hospital
Hu H et al.	8.37	5.78	19	10.24	7.51	86	Renmin Hospital of Wuhan University (2020 Feb 7- 2020 Mar 7)
Wang L et al. (b)	10.33	5.31	65	10	4.47	274	Renmin Hospital of Wuhan University (2020 Jan 1- 2020 Feb 6)
Yao Q et al.	7.33	2.52	12	6	3.32	13	Huanggang Central Hospital
Zhou F et al.	11.33	5.33	54	10.67	3.75	137	Jinyintan Hospital and Wuhan Pulmonary Hospital

Table S108. Sensitivity Analyses of Dichotomous Prognostic Factors.

Prognostic factors	Pre-sensitivity analysis		Post-sensitivity analysis, RR (95% Cl)		
	I^2 (%)	RR (95% Cl)	Omitted largest study	Omitted smallest study	Fixed-effects model
Advanced age	86	2.31 [1.99, 2.67]	2.38 [1.96, 2.90]	2.33 [2.00,2.71]	2.12 [2.03,2.21]
Male sex	65	1.30 [1.17, 1.44]	1.34 [1.22, 1.47]	1.30 [1.16, 1.45]	1.21 [1.15, 1.27]
Any comorbidity	97	2.85 [1.47, 5.51]	2.85 [1.17, 6.96]	3.16 [1.42, 7.05]	1.99 [1.80, 2.20]
Hypertension	82	2.11 [1.49, 2.99]	1.80 [1.48, 2.19]	2.25 [1.55, 3.28]	1.90 [1.66, 2.17]
Chronic respiratory disease	50	3.84 [1.81, 8.16]	2.70 [1.48, 4.93]	3.87 [1.38, 10.9]	3.29 [1.99, 5.44]
Malignancy	54	2.03 [0.92, 4.44]	**<u>3.08 [1.39, 6.82]</u>**	2.31 [0.92, 5.76]	1.30 [0.96, 1.76]
Anorexia	64	1.12 [0.81, 1.55]	**<u>1.24 [1.11, 1.39]</u>**	1.02 [0.65, 1.59]	1.11 [0.91, 1.34]
Nausea	61	1.25 [0.46, 3.40]	1.37 [0.37, 5.07]	0.73 [0.35, 1.55]	1.15 [0.66, 1.98]
Antiviral therapy	92	0.87 [0.71, 1.08]	0.86 [0.59, 1.26]	0.83 [0.64, 1.08]	**<u>0.83 [0.76, 0.90]</u>**
Antibiotic therapy	94	1.20 [1.02, 1.40]	1.28 [1.00, 1.63]	1.28 [1.04, 1.57]	1.10 [1.06, 1.15]
Immunoglobulin therapy	90	2.00 [0.82, 4.88]	2.32 [0.57, 9.41]	2.25 [0.88, 5.76]	**<u>1.69 [1.37, 2.08]</u>**
Corticosteroid therapy	88	1.79 [1.25, 2.55]	2.02 [1.36, 3.02]	1.91 [1.25, 2.93]	1.45 [1.31, 1.60]
White blood cell count ≥10×10^9/L	84	6.41 [2.18, 18.8]	4.97 [1.26, 19.6]	10.2 [3.52, 29.3]	6.74 [4.54, 10.0]
Platelet count <100×10^9/L	65	3.34 [0.75, 14.8]	1.82 [0.66, 5.06]	3.82 [0.26, 56.7]	4.09 [1.95, 8.59]
Platelet count <125×10^9/L	92	4.65 [2.13, 10.2]	Not available	Not available	5.95 [5.06, 7.00]
AST >40 U/L	80	2.01 [1.13, 3.58]	1.52 [1.10, 2.11]	2.36 [1.12, 4.97]	2.39 [1.84, 3.11]
Procalcitonin ≥0.1 ng/mL	69	2.96 [1.67, 5.25]	Not available	Not available	3.18 [2.28, 4.44]
Procalcitonin ≥0.05 ng/mL	66	1.72 [1.23, 2.43]	Not available	Not available	1.88 [1.61, 2.19]
C-reactive protein ≥10 mg/L	97	1.58 [0.68, 3.63]	Not available	Not available	**<u>1.42 [1.27, 1.59]</u>**
D-dimer >1.0 μg/mL	51	2.89 [1.94, 4.29]	Not available	Not available	3.12 [2.35, 4.14]
Sepsis	54	2.59 [2.11, 3.17]	2.84 [1.83, 4.40]	2.38 [2.08, 2.73]	2.46 [2.16, 2.81]
Acute respiratory distress syndrome	97	6.82 [2.56, 18.2]	5.36 [2.25, 12.8]	5.71 [2.01, 16.2]	3.83 [3.36, 4.37]
Acute cardiac injury	72	8.22 [4.95, 13.7]	10.6 [4.87, 22.8]	7.07 [4.30, 11.6]	6.98 [5.56, 8.76]
Acute infection	93	9.78 [2.05, 46.7]	17.1 [3.42, 86.0]	7.00 [1.39, 35.3]	3.87 [3.14, 4.77]
Acute kidney injury	55	9.64 [6.01, 15.4]	13.0 [5.48, 30.8]	8.89 [5.26, 15.0]	8.47 [7.31, 9.80]
Heart failure	76	4.18 [2.37, 7.36]	5.06 [1.97, 13.0]	5.13 [2.46, 10.7]	4.47 [3.36, 5.95]
Arrhythmia	85	4.86 [1.24, 19.0]	Not available	Not available	3.93 [2.44, 6.34]
Shock	82	12.6 [1.25, 127.1]	33.7 [1.04, 1093]	**<u>16.6 [0.08, 3391]</u>**	21.4 [8.32, 54.8]
Acute liver injury	93	3.78 [1.18, 12.1]	**<u>1.91 [0.97, 3.73]</u>**	5.44 [1.33, 22.2]	3.43 [2.71, 4.34]
Highest temperature ≥39 °C	80	1.48 [0.36, 6.12]	Not available	Not available	0.93 [0.61, 1.44]

Table S109. Sensitivity Analyses of Continuous Prognostic Factors.

Prognostic factors	Pre-sensitivity analysis		Post-sensitivity analysis, MD (95% Cl)		
	I^2 (%)	MD (95% Cl)	Omitted largest study	Omitted smallest study	Fixed-effects model
Age	89	13.9 [8.95, 18.9]	14.2[8.54, 19.9]	14.6 [9.32, 19.8]	11.8 [10.3, 13.3]
Respiratory rate, breaths per min	70	2.94 [1.10, 4.79]	2.76 [0.36, 5.17]	3.96 [2.92, 5.00]	3.09 [2.17, 4.01]
PaO_2, mm Hg	89	-28.7 [-52.7, -4.65]	**-37.0 [-88.4, 14.6]**	-15.1 [-22.2, -7.91]	-20.0 [-26.8, -13.2]
SpO_2	96	-7.45 [-15.9, 0.96]	-4.88 [-13.3, 3.54]	-6.50 [-17.4, 4.38]	**-4.29 [-5.62, -2.95]**
PaO_2/FiO_2	92	-122.6 [-198.2, -47.0]	-148.8 [-291.0, -6.49]	-161.0 [-270.2, -51.8]	-66.5 [-83.6, -49.3]
Neutrophil count, $\times 10^9$/L	61	3.86 [2.74, 4.99]	3.85 [2.45, 5.25]	3.92 [2.70, 5.14]	4.20 [3.59, 4.81]
Lymphocyte count, $\times 10^9$/L	78	-0.34 [-0.47, -0.21]	-0.31 [-0.48, -0.14]	-0.37 [-0.49, -0.25]	-0.38 [-0.43, -0.33]
Monocyte count, $\times 10^9$/L	62	-0.06 [-0.11, -0.01]	**-0.04 [-0.09, 0.01]**	**-0.05 [-0.12, 0.01]**	-0.06 [-0.09, -0.03]
Platelet count, $\times 10^9$/L	59	-34.0 [-57.1, -10.9]	**-25.0 [-55.6, 5.53]**	-43.8 [-60.2, -27.5]	-40.7 [-53.4, -28.0]
Albumin, g/L	87	-4.05 [-6.51, -1.59]	-3.31 [-6.24, -0.38]	-3.60 [-6.43, -0.77]	-4.81 [-5.52, -4.10]
ALT, U/L	60	3.58 [-0.72, 7.87]	4.03 [-1.17, 9.23]	5.11 [1.12, 9.10]	3.75 [1.29, 6.22]
AST, U/L	73	14.8 [9.56, 20.1]	14.5 [7.67, 21.4]	14.9 [9.49, 20.4]	15.2 [12.1, 18.2]
Creatinine, μmol/L	64	18.0 [10.1, 25.9]	17.1 [7.91, 26.2]	17.3 [8.79, 25.8]	16.0 [11.9, 20.0]
Blood urea nitrogen, mmol/L	80	3.94 [1.97, 5.90]	Not available	Not available	4.23 [3.41, 5.04]
Urea, mmol/L	71	3.41 [1.81, 5.02]	Not available	Not available	3.11 [2.36, 3.87]
APTT, seconds	91	-0.47 [-3.31, 2.37]	-1.01 [-4.89, 2.86]	**0.94 [0.13, 1.76]**	-0.07 [-0.81, 0.66]
C-reactive protein, mg/L	92	55.9 [27.3, 84.5]	53.5 [21.0, 86.0]	64.9 [46.1, 83.7]	45.7 [38.6, 52.8]
Procalcitonin, ng/mL	76	0.27 [0.14, 0.40]	0.23 [0.09, 0.38]	0.26 [0.14, 0.38]	0.25 [0.20, 0.29]
Lactate dehydrogenase, U/L	81	225.6 [153.8, 297.3]	236.6 [142.2, 331.0]	235.9 [162.3, 309.4]	246.4 [218.9, 274.0]
Creatine kinase, U/L	75	59.3 [26.2, 92.5]	59.9 [16.2, 103.6]	70.5 [38.3, 102.6]	58.1 [43.4, 72.7]
γ-glutamyl transpeptidase, U/L	92	4.91 [-16.5, 26.4]	Not available	Not available	**8.52 [3.02, 14.0]**
Creatine kinase-MB, U/L	74	3.11 [0.84, 5.37]	5.12 [0.10, 10.1]	2.41 [0.39, 4.43]	1.68 [1.15, 2.21]
hs-cTnI, ng/mL	87	68.6 [29.3, 107.9]	49.8 [17.3, 82.4]	94.6 [30.3, 159.0]	50.5 [38.4, 62.5]
Interleukin-6, pg/mL	96	47.8 [10.6, 85.0]	**34.1 [-2.65, 70.8]**	45.6 [7.58, 83.6]	5.61 [3.91, 7.30]

Study or Subgroup	Non-survivors Mean	SD	Total	Survivors Mean	SD	Total	Weight	Mean Difference IV, Random, 95% CI
Cao J et al.	72	14.55	17	55.33	14.33	85	11.2%	16.67 [9.11, 24.23]
Du RH et al.	70.2	7.7	21	56	13.5	158	13.9%	14.20 [10.29, 18.11]
Hu H et al.	75.05	12.94	19	57.71	15.34	86	11.9%	17.34 [10.68, 24.00]
Tang N et al. (a)	68.7	11.4	134	63.7	12.2	315	14.7%	5.00 [2.65, 7.35]
Wu C et al.	67.6	12.03	44	47.33	10.51	117	13.8%	20.27 [16.24, 24.30]
Xu B et al.	72.75	7.23	28	55	17.26	117	13.7%	17.75 [13.63, 21.87]
Yao Q et al.	63.17	18.87	12	56.67	10.8	13	8.0%	6.50 [-5.68, 18.68]
Zhang J et al.	68.7	13.36	25	56.7	18.57	638	12.8%	12.00 [6.57, 17.43]
**Total (95% CI)**			**300**			**1529**	**100.0%**	**13.93 [8.95, 18.92]**

Heterogeneity: Tau² = 42.73; Chi² = 63.46, df = 7 (P < 0.00001); I² = 89%
Test for overall effect: Z = 5.48 (P < 0.00001)

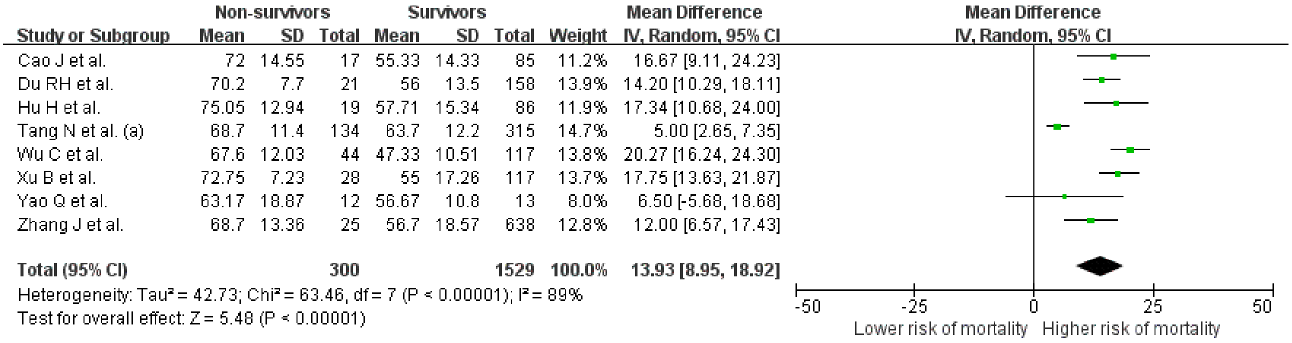

Figure S1. Forest plot of mean difference in age between nonsurvivors and survivors

Study or Subgroup	Non-survivors Events	Total	Survivors Events	Total	Weight	Risk Ratio M-H, Random, 95% CI
Chen T et al. (a)	94	113	59	161	11.7%	2.27 [1.82, 2.83]
Chen T et al. (b)	19	26	36	177	7.8%	3.59 [2.47, 5.22]
Du RH et al.	17	21	48	158	9.2%	2.66 [1.95, 3.65]
Grasselli L et al.	283	402	503	1179	14.9%	1.65 [1.51, 1.81]
Luo M et al.	32	69	48	406	8.0%	3.92 [2.72, 5.66]
Miyashita H et al.	371	555	1588	5133	15.3%	2.16 [2.01, 2.32]
Richardson S et al.	419	553	676	2081	15.2%	2.33 [2.16, 2.52]
Yang X et al. (b)	20	32	7	20	3.8%	1.79 [0.93, 3.44]
Yao Q et al.	6	12	11	96	2.8%	4.36 [1.97, 9.65]
Zhang J et al.	19	25	296	638	11.3%	1.64 [1.29, 2.07]
**Total (95% CI)**		**1808**		**10049**	**100.0%**	**2.31 [1.99, 2.67]**
Total events	1280		3272			

Heterogeneity: Tau² = 0.04; Chi² = 63.67, df = 9 (P < 0.00001); I² = 86%
Test for overall effect: Z = 11.13 (P < 0.00001)

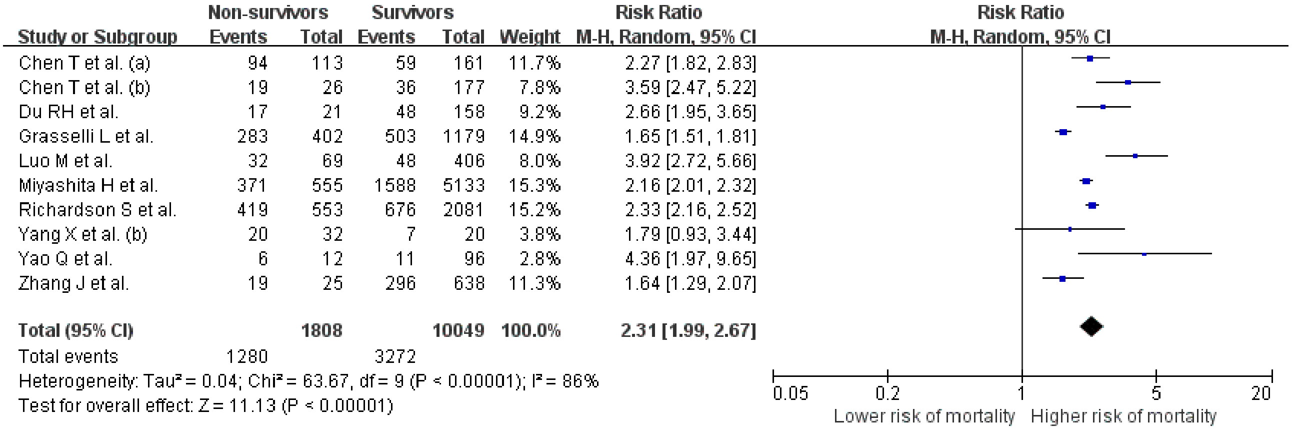

Figure S2. Forest plot of association between advanced age and mortality

Study or Subgroup	Non-survivors Events	Total	Survivors Events	Total	Weight	Risk Ratio M-H, Random, 95% CI
Du RH et al.	10	21	87	158	3.9%	0.86 [0.54, 1.38]
Hu H et al.	14	19	48	86	6.5%	1.32 [0.95, 1.83]
Li J et al.	40	64	257	594	10.3%	1.44 [1.17, 1.78]
Liu Y et al. (a)	25	33	89	212	8.8%	1.80 [1.41, 2.32]
Luo M et al.	46	69	185	406	10.9%	1.46 [1.20, 1.78]
Richardson S et al.	337	553	1162	2081	16.2%	1.09 [1.01, 1.18]
Tang N et al. (a)	90	134	178	315	12.9%	1.19 [1.02, 1.39]
Xu B et al.	17	28	59	117	6.0%	1.20 [0.85, 1.71]
Yang X et al. (a)	156	238	620	1238	15.0%	1.31 [1.18, 1.46]
Yao Q et al.	7	12	36	96	3.1%	1.56 [0.90, 2.68]
Zhang J et al.	15	25	306	638	6.4%	1.25 [0.90, 1.74]
**Total (95% CI)**		**1196**		**5941**	**100.0%**	**1.30 [1.17, 1.44]**
Total events	757		3027			

Heterogeneity: Tau² = 0.02; Chi² = 28.28, df = 10 (P = 0.002); I² = 65%
Test for overall effect: Z = 4.87 (P < 0.00001)

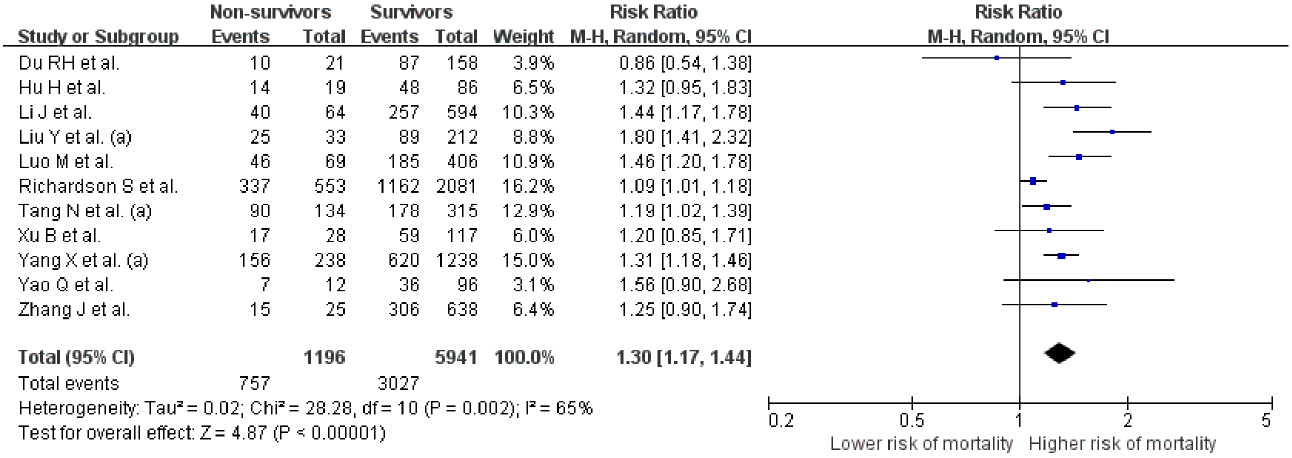

Figure S3. Forest plot of association between male sex and mortality

Study or Subgroup	Non-survivors Events	Non-survivors Total	Survivors Events	Survivors Total	Weight	Risk Ratio M-H, Random, 95% CI
Chen T et al. (a)	7	113	5	161	31.6%	1.99 [0.65, 6.13]
Liu Y et al. (a)	3	33	7	212	26.0%	2.75 [0.75, 10.12]
Yao Q et al.	3	12	1	96	11.6%	24.00 [2.71, 212.77]
Zhou F et al.	5	54	6	137	30.8%	2.11 [0.67, 6.64]
**Total (95% CI)**		**212**		**606**	**100.0%**	**2.95 [1.32, 6.58]**
Total events	18		19			

Heterogeneity: Tau^2 = 0.20; Chi^2 = 4.31, df = 3 (P = 0.23); I^2 = 30%
Test for overall effect: Z = 2.64 (P = 0.008)

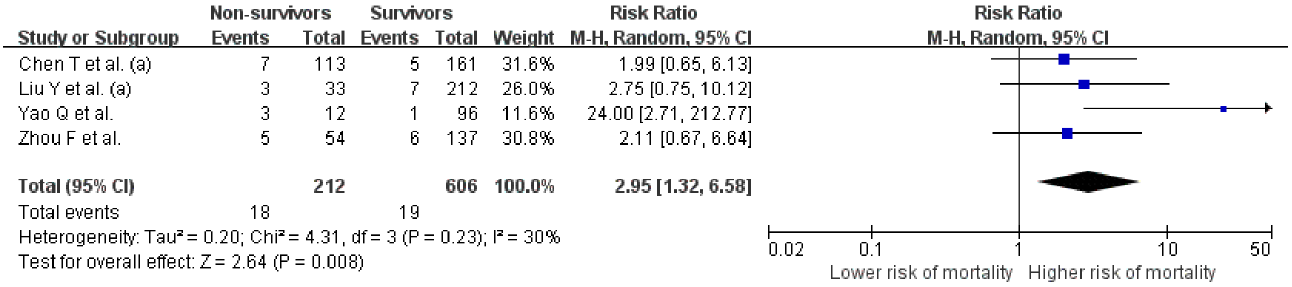

Figure S4. Forest plot of association between current smoking and mortality

Study or Subgroup	Non-survivors Events	Non-survivors Total	Survivors Events	Survivors Total	Weight	Risk Ratio M-H, Random, 95% CI
Cao J et al.	13	17	34	85	19.8%	1.91 [1.32, 2.77]
Guan WJ et al.	35	50	364	1540	20.7%	2.96 [2.42, 3.63]
Luo M et al.	45	69	15	406	18.6%	17.65 [10.44, 29.86]
Tang N et al. (a)	91	134	181	315	20.8%	1.18 [1.02, 1.37]
Xu B et al.	21	28	47	117	20.1%	1.87 [1.37, 2.54]
**Total (95% CI)**		**298**		**2463**	**100.0%**	**2.85 [1.47, 5.51]**
Total events	205		641			

Heterogeneity: Tau^2 = 0.54; Chi^2 = 127.47, df = 4 (P < 0.00001); I^2 = 97%
Test for overall effect: Z = 3.11 (P = 0.002)

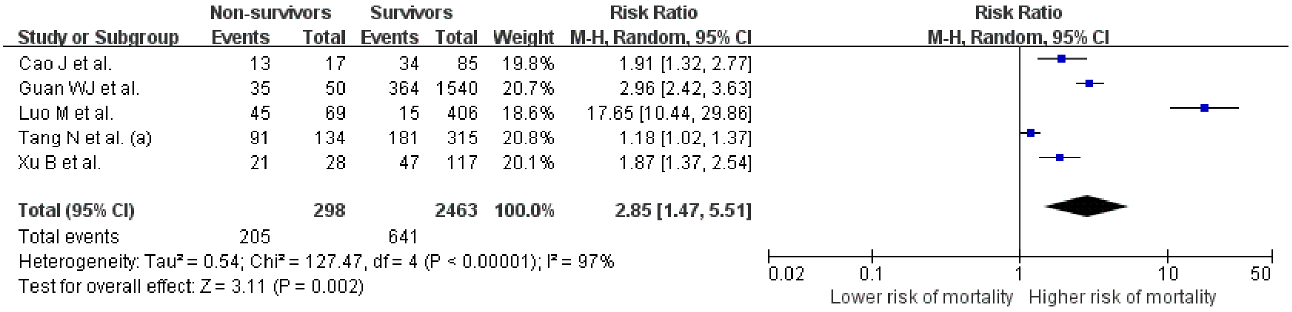

Figure S5. Forest plot of association between preexisting any comorbidity and mortality

Study or Subgroup	Non-survivors Events	Non-survivors Total	Survivors Events	Survivors Total	Weight	Risk Ratio M-H, Random, 95% CI
Cao J et al.	3	17	1	85	11.7%	15.00 [1.66, 135.71]
Chen T et al. (a)	4	113	1	161	11.9%	5.70 [0.65, 50.32]
Guan WJ et al.	5	50	16	1540	61.0%	9.63 [3.67, 25.24]
Xu B et al.	2	28	2	117	15.4%	4.18 [0.62, 28.39]
**Total (95% CI)**		**208**		**1903**	**100.0%**	**8.37 [3.94, 17.77]**
Total events	14		20			

Heterogeneity: Tau^2 = 0.00; Chi^2 = 1.02, df = 3 (P = 0.80); I^2 = 0%
Test for overall effect: Z = 5.53 (P < 0.00001)

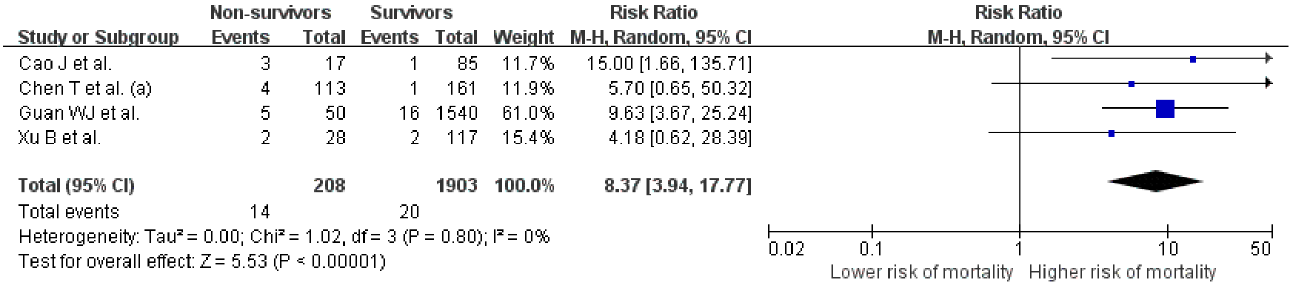

Figure S6. Forest plot of association between preexisting chronic kidney disease and mortality

Study or Subgroup	Non-survivors Events	Non-survivors Total	Survivors Events	Survivors Total	Weight	Risk Ratio M-H, Random, 95% CI
Cao J et al.	3	17	3	85	20.3%	5.00 [1.10, 22.71]
Chen T et al. (a)	4	113	0	161	5.5%	12.79 [0.70, 235.22]
Guan WJ et al.	6	50	24	1540	64.6%	7.70 [3.29, 18.00]
Hu H et al.	3	19	1	86	9.6%	13.58 [1.49, 123.53]
**Total (95% CI)**		**199**		**1872**	**100.0%**	**7.66 [3.87, 15.15]**
Total events	16		28			

Heterogeneity: Tau^2 = 0.00; Chi^2 = 0.72, df = 3 (P = 0.87); I^2 = 0%
Test for overall effect: Z = 5.85 (P < 0.00001)

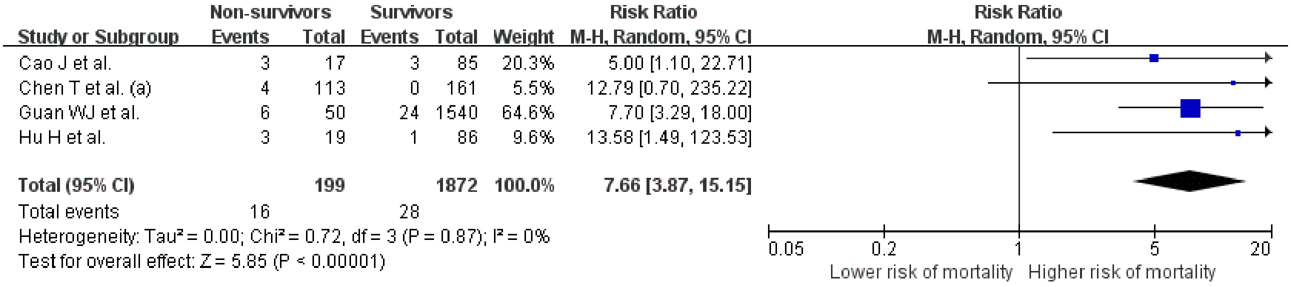

Figure S7. Forest plot of association between cerebrovascular disease and mortality

Study or Subgroup	Non-survivors Events	Non-survivors Total	Survivors Events	Survivors Total	Weight	Risk Ratio M-H, Random, 95% CI
Chen T et al. (a)	11	113	7	161	26.2%	2.24 [0.90, 5.60]
Guan WJ et al.	6	50	18	1540	27.1%	10.27 [4.26, 24.75]
Hu H et al.	5	19	7	86	23.7%	3.23 [1.15, 9.10]
Liu Y et al. (a)	3	33	5	212	17.5%	3.85 [0.97, 15.38]
Xu B et al.	0	28	2	117	5.5%	0.81 [0.04, 16.49]
**Total (95% CI)**		**243**		**2116**	**100.0%**	**3.84 [1.81, 8.16]**
Total events	25		39			

Heterogeneity: Tau² = 0.35; Chi² = 7.98, df = 4 (P = 0.09); I² = 50%
Test for overall effect: Z = 3.50 (P = 0.0005)

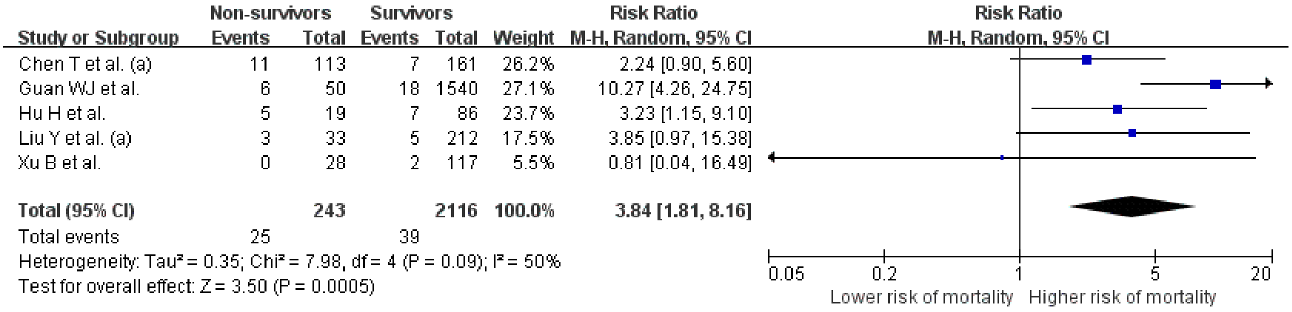

Figure S8. Forest plot of association between preexisting chronic respiratory disease and mortality

Study or Subgroup	Non-survivors Events	Non-survivors Total	Survivors Events	Survivors Total	Weight	Risk Ratio M-H, Random, 95% CI
Cao J et al.	3	17	2	85	4.4%	7.50 [1.35, 41.54]
Chen T et al. (a)	16	113	7	161	15.8%	3.26 [1.39, 7.66]
Guan WJ et al.	8	50	51	1540	22.4%	4.83 [2.42, 9.63]
Guo T et al.	29	43	37	144	54.4%	2.62 [1.86, 3.71]
Hu H et al.	1	19	5	86	3.0%	0.91 [0.11, 7.31]
**Total (95% CI)**		**242**		**2016**	**100.0%**	**3.16 [2.19, 4.56]**
Total events	57		102			

Heterogeneity: Tau² = 0.03; Chi² = 4.80, df = 4 (P = 0.31); I² = 17%
Test for overall effect: Z = 6.12 (P < 0.00001)

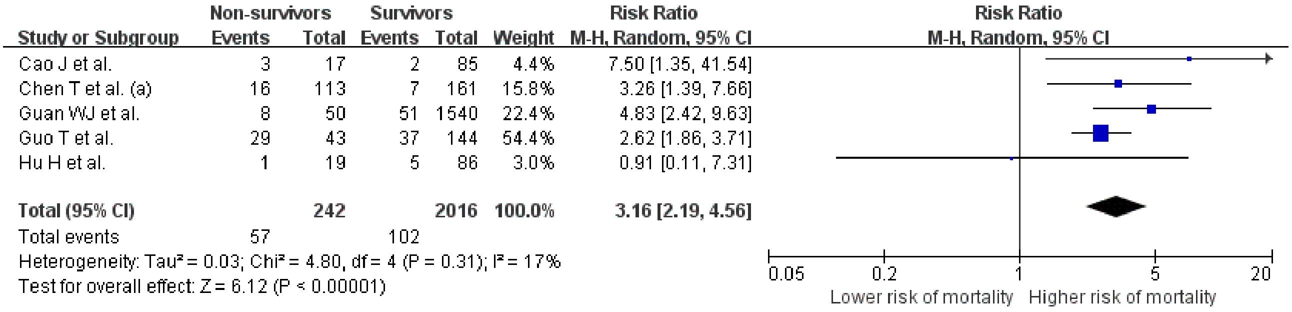

Figure S9. Forest plot of association between preexisting cardiovascular disease and mortality

Study or Subgroup	Non-survivors Events	Non-survivors Total	Survivors Events	Survivors Total	Weight	Risk Ratio M-H, Random, 95% CI
Chen T et al. (a)	24	113	23	161	31.2%	1.49 [0.88, 2.50]
Guan WJ et al.	13	50	117	1540	32.1%	3.42 [2.08, 5.64]
Hu H et al.	0	19	4	86	2.6%	0.48 [0.03, 8.62]
Liu Y et al. (a)	7	33	16	212	20.4%	2.81 [1.25, 6.31]
Luo M et al.	4	69	13	406	13.8%	1.81 [0.61, 5.39]
**Total (95% CI)**		**284**		**2405**	**100.0%**	**2.21 [1.37, 3.56]**
Total events	48		173			

Heterogeneity: Tau² = 0.12; Chi² = 7.22, df = 4 (P = 0.12); I² = 45%
Test for overall effect: Z = 3.25 (P = 0.001)

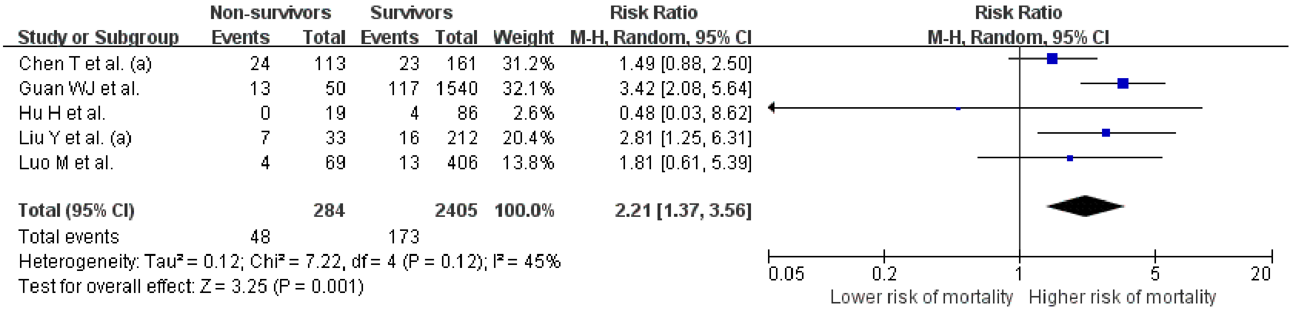

Figure S10. Forest plot of association between preexisting diabetes mellitus and mortality

Study or Subgroup	Non-survivors Events	Non-survivors Total	Survivors Events	Survivors Total	Weight	Risk Ratio M-H, Random, 95% CI
Chen T et al. (a)	54	113	39	161	18.8%	1.97 [1.41, 2.76]
Grasselli L et al.	195	309	84	212	21.3%	1.59 [1.32, 1.92]
Guan WJ et al.	28	50	241	1540	19.9%	3.58 [2.73, 4.70]
Hu H et al.	6	19	22	86	11.0%	1.23 [0.58, 2.62]
Liu Y et al. (a)	15	33	37	212	16.0%	2.60 [1.62, 4.19]
Xu B et al.	10	28	21	117	13.1%	1.99 [1.06, 3.74]
**Total (95% CI)**		**552**		**2328**	**100.0%**	**2.11 [1.49, 2.99]**
Total events	308		444			

Heterogeneity: Tau² = 0.14; Chi² = 27.35, df = 5 (P < 0.0001); I² = 82%
Test for overall effect: Z = 4.18 (P < 0.0001)

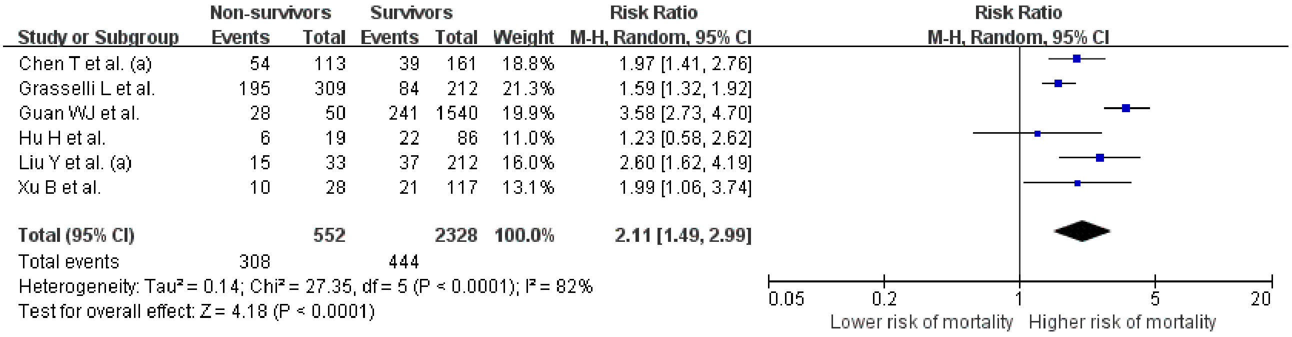

Figure S11. Forest plot of association between preexisting hypertension and mortality

	Non-survivors		Survivors			Risk Ratio
Study or Subgroup	Events	Total	Events	Total	Weight	M-H, Random, 95% CI
Chen T et al. (a)	5	113	2	161	14.9%	3.56 [0.70, 18.04]
Guan WJ et al.	3	50	15	1540	20.7%	6.16 [1.84, 20.60]
Hu H et al.	1	19	5	86	10.5%	0.91 [0.11, 7.31]
Liu Y et al. (a)	2	33	7	212	16.0%	1.84 [0.40, 8.46]
Miyashita H et al.	37	555	297	5133	37.9%	1.15 [0.83, 1.60]
**Total (95% CI)**		**770**		**7132**	**100.0%**	**2.03 [0.92, 4.44]**
Total events	48		326			

Heterogeneity: Tau² = 0.39; Chi² = 8.69, df = 4 (P = 0.07); I² = 54%
Test for overall effect: Z = 1.76 (P = 0.08)

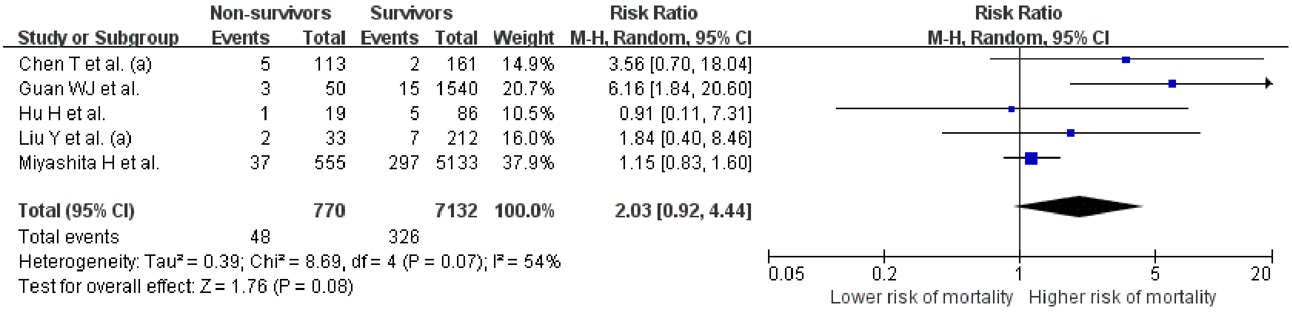

Figure S12. Forest plot of association between preexisting malignancy and mortality

	Non-survivors		Survivors			Risk Ratio
Study or Subgroup	Events	Total	Events	Total	Weight	M-H, Random, 95% CI
Chen T et al. (a)	1	113	2	161	12.0%	0.71 [0.07, 7.76]
Du RH et al.	4	21	17	158	70.0%	1.77 [0.66, 4.76]
Zhang J et al.	1	25	30	638	18.0%	0.85 [0.12, 5.99]
**Total (95% CI)**		**159**		**957**	**100.0%**	**1.39 [0.61, 3.18]**
Total events	6		49			

Heterogeneity: Tau² = 0.00; Chi² = 0.81, df = 2 (P = 0.67); I² = 0%
Test for overall effect: Z = 0.78 (P = 0.43)

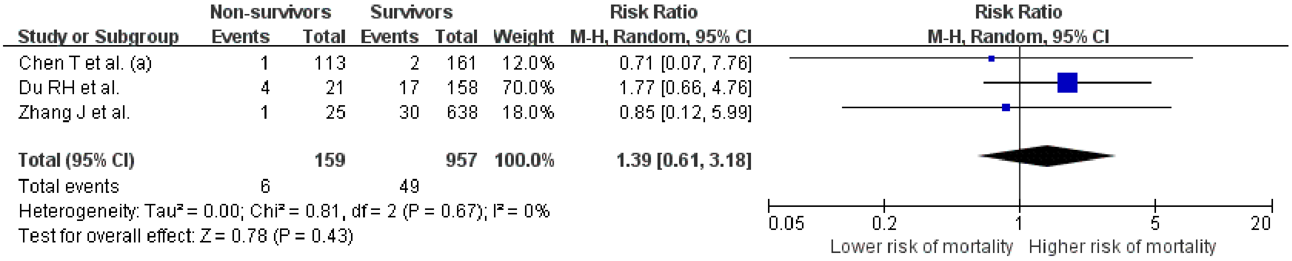

Figure S13. Forest plot of association between preexisting gastrointestinal disease and mortality

	Non-survivors		Survivors			Risk Ratio
Study or Subgroup	Events	Total	Events	Total	Weight	M-H, Random, 95% CI
Chen T et al. (a)	5	113	6	161	53.3%	1.19 [0.37, 3.80]
Guan WJ et al.	1	50	27	1540	18.5%	1.14 [0.16, 8.23]
Liu Y et al. (a)	2	33	5	212	28.2%	2.57 [0.52, 12.71]
**Total (95% CI)**		**196**		**1913**	**100.0%**	**1.47 [0.63, 3.42]**
Total events	8		38			

Heterogeneity: Tau² = 0.00; Chi² = 0.67, df = 2 (P = 0.72); I² = 0%
Test for overall effect: Z = 0.88 (P = 0.38)

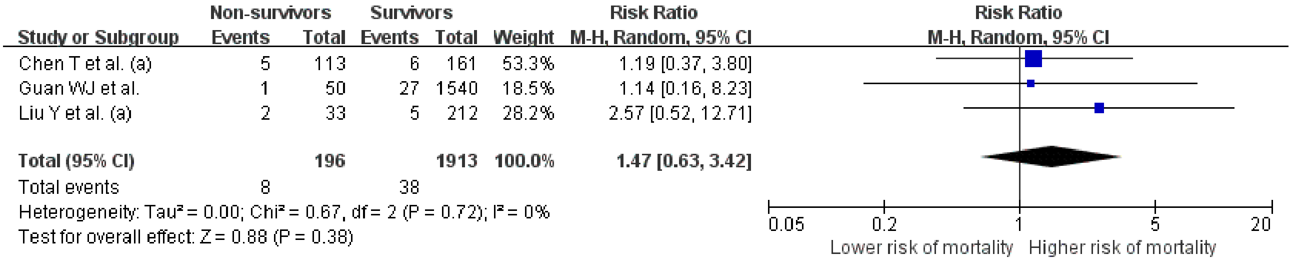

Figure S14. Forest plot of association between preexisting chronic liver disease and mortality

	Non-survivors		Survivors			Risk Ratio
Study or Subgroup	Events	Total	Events	Total	Weight	M-H, Random, 95% CI
Chen T et al. (a)	1	113	1	161	38.3%	1.42 [0.09, 22.54]
Wang L et al. (b)	1	65	4	274	61.7%	1.05 [0.12, 9.27]
**Total (95% CI)**		**178**		**435**	**100.0%**	**1.18 [0.21, 6.53]**
Total events	2		5			

Heterogeneity: Tau² = 0.00; Chi² = 0.03, df = 1 (P = 0.87); I² = 0%
Test for overall effect: Z = 0.19 (P = 0.85)

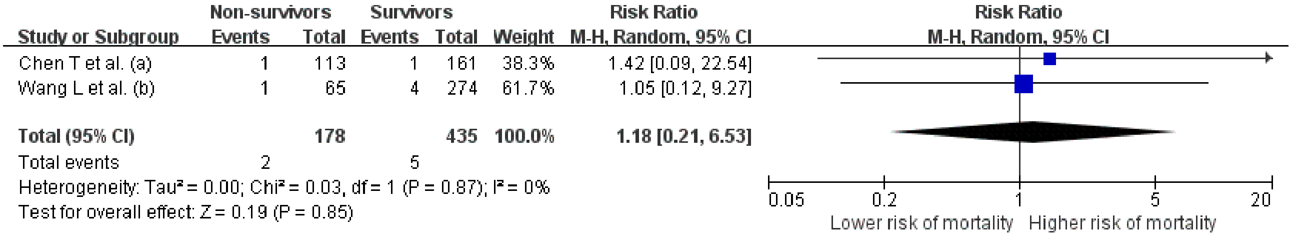

Figure S15. Forest plot of association between preexisting autoimmune disease and mortality

Study or Subgroup	Non-survivors Events	Non-survivors Total	Survivors Events	Survivors Total	Weight	Risk Ratio M-H, Random, 95% CI
Cao J et al.	12	17	61	85	1.6%	0.98 [0.70, 1.37]
Chen T et al. (a)	104	113	145	161	32.9%	1.02 [0.95, 1.10]
Du RH et al.	21	21	156	158	41.2%	0.99 [0.93, 1.06]
Wu C et al.	39	44	149	157	14.6%	0.93 [0.84, 1.04]
Xu B et al.	23	28	96	117	4.9%	1.00 [0.83, 1.21]
Yao Q et al.	8	12	72	96	1.1%	0.89 [0.59, 1.35]
Zhang J et al.	19	25	508	638	3.7%	0.95 [0.76, 1.19]
**Total (95% CI)**		**260**		**1412**	**100.0%**	**0.99 [0.95, 1.03]**
Total events	226		1187			

Heterogeneity: $Tau^2 = 0.00$; $Chi^2 = 2.26$, df = 6 (P = 0.89); $I^2 = 0\%$
Test for overall effect: Z = 0.41 (P = 0.68)

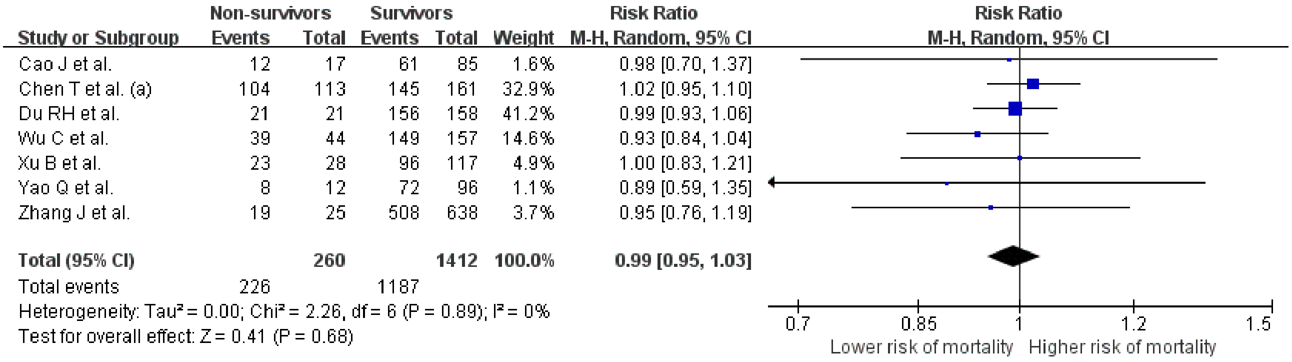

Figure S16. Forest plot of association between symptoms of fever and mortality

Study or Subgroup	Non-survivors Events	Non-survivors Total	Survivors Events	Survivors Total	Weight	Risk Ratio M-H, Random, 95% CI
Cao J et al.	8	17	42	85	2.6%	0.95 [0.55, 1.65]
Chen T et al. (a)	79	113	106	161	28.7%	1.06 [0.90, 1.25]
Du RH et al.	14	21	132	158	8.0%	0.80 [0.59, 1.09]
Wu C et al.	33	44	130	157	22.6%	0.91 [0.75, 1.09]
Xu B et al.	23	28	89	117	19.3%	1.08 [0.88, 1.32]
Yao Q et al.	10	12	74	96	10.2%	1.08 [0.82, 1.42]
Zhang J et al.	16	25	394	638	8.6%	1.04 [0.77, 1.40]
**Total (95% CI)**		**260**		**1412**	**100.0%**	**1.00 [0.92, 1.09]**
Total events	183		967			

Heterogeneity: $Tau^2 = 0.00$; $Chi^2 = 4.63$, df = 6 (P = 0.59); $I^2 = 0\%$
Test for overall effect: Z = 0.03 (P = 0.98)

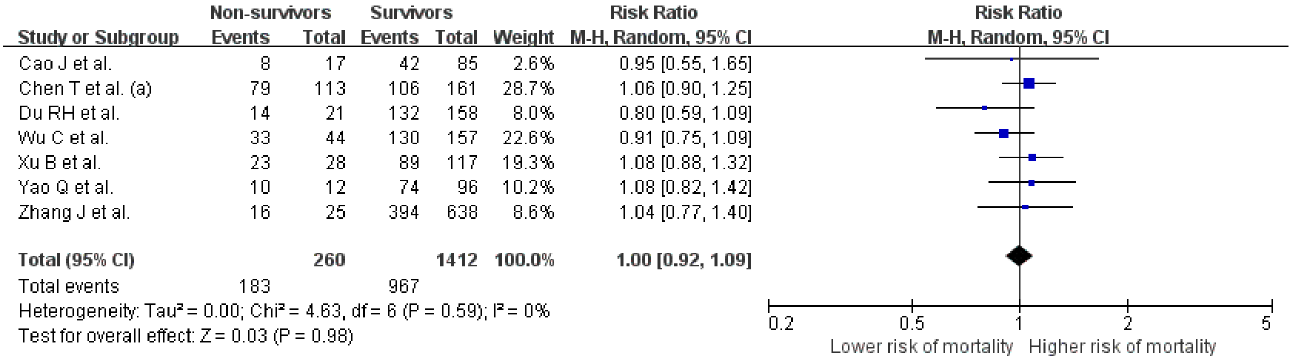

Figure S17. Forest plot of association between symptoms of cough and mortality

Study or Subgroup	Non-survivors Events	Non-survivors Total	Survivors Events	Survivors Total	Weight	Risk Ratio M-H, Random, 95% CI
Chen T et al. (a)	70	113	50	161	22.9%	1.99 [1.52, 2.62]
Du RH et al.	18	21	71	158	26.5%	1.91 [1.49, 2.44]
Wu C et al.	29	44	51	157	18.8%	2.03 [1.49, 2.77]
Xie J et al.	26	36	43	104	19.2%	1.75 [1.29, 2.37]
Yao Q et al.	6	12	9	96	3.1%	5.33 [2.30, 12.36]
Zhang J et al.	11	25	150	638	9.5%	1.87 [1.18, 2.98]
**Total (95% CI)**		**251**		**1314**	**100.0%**	**1.98 [1.70, 2.30]**
Total events	160		374			

Heterogeneity: $Tau^2 = 0.01$; $Chi^2 = 6.17$, df = 5 (P = 0.29); $I^2 = 19\%$
Test for overall effect: Z = 8.82 ($P < 0.00001$)

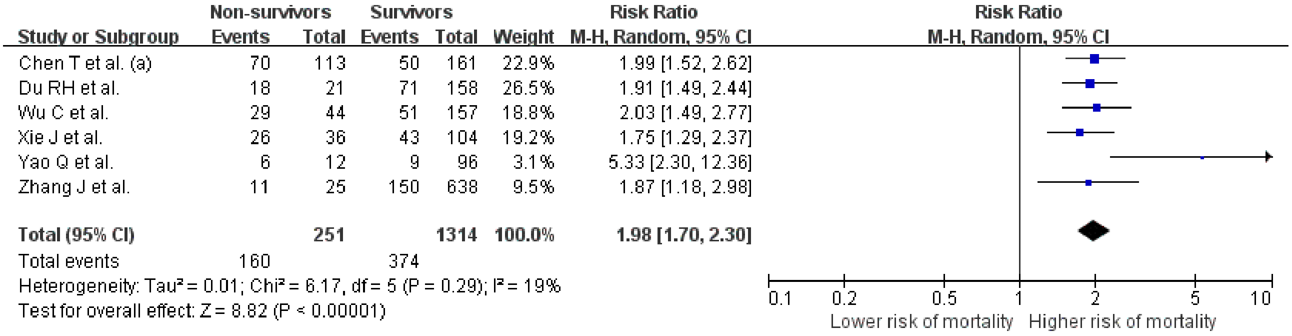

Figure S18. Forest plot of association between symptoms of dyspnea and mortality

Study or Subgroup	Non-survivors Events	Non-survivors Total	Survivors Events	Survivors Total	Weight	Risk Ratio M-H, Random, 95% CI
Chen T et al. (a)	55	113	48	161	75.7%	1.63 [1.21, 2.21]
Zhang J et al.	6	25	148	638	24.3%	1.03 [0.51, 2.11]
**Total (95% CI)**		**138**		**799**	**100.0%**	**1.46 [0.99, 2.15]**
Total events	61		196			

Heterogeneity: $Tau^2 = 0.03$; $Chi^2 = 1.34$, df = 1 (P = 0.25); $I^2 = 26\%$
Test for overall effect: Z = 1.93 (P = 0.05)

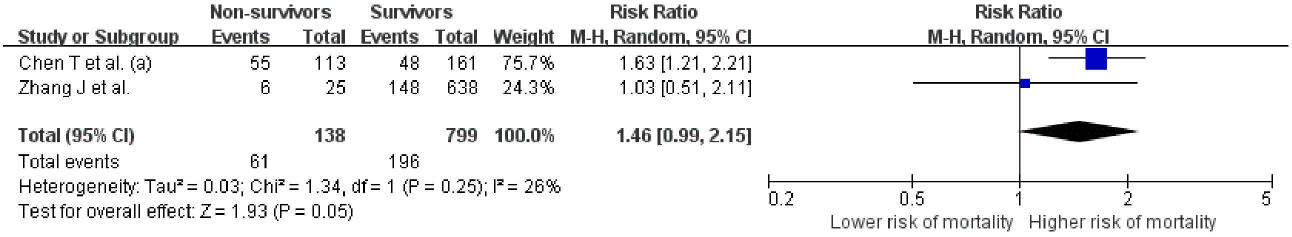

Figure S19. Forest plot of association between symptoms of chest tightness and mortality

Study or Subgroup	Non-survivors Events	Non-survivors Total	Survivors Events	Survivors Total	Weight	Risk Ratio M-H, Random, 95% CI
Chen T et al. (a)	35	113	48	161	26.0%	1.04 [0.72, 1.49]
Du RH et al.	12	21	43	158	21.2%	2.10 [1.34, 3.29]
Wu C et al.	19	44	64	157	24.5%	1.06 [0.72, 1.56]
Yao Q et al.	5	12	29	96	11.2%	1.38 [0.66, 2.88]
Zhang J et al.	9	25	157	638	17.1%	1.46 [0.85, 2.51]
**Total (95% CI)**		**215**		**1210**	**100.0%**	**1.33 [1.00, 1.76]**
Total events	80		341			

Heterogeneity: Tau² = 0.05; Chi² = 7.31, df = 4 (P = 0.12); I² = 45%
Test for overall effect: Z = 1.95 (P = 0.05)

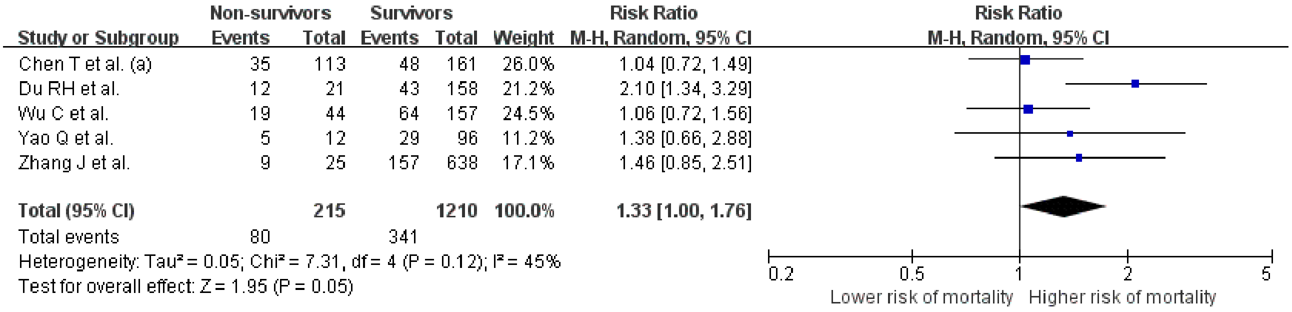

Figure S20. Forest plot of association between symptoms of sputum production and mortality

Study or Subgroup	Non-survivors Events	Non-survivors Total	Survivors Events	Survivors Total	Weight	Risk Ratio M-H, Random, 95% CI
Cao J et al.	9	17	47	85	14.3%	0.96 [0.59, 1.56]
Chen T et al. (a)	64	113	73	161	62.1%	1.25 [0.99, 1.58]
Zhang J et al.	9	25	199	638	11.9%	1.15 [0.68, 1.97]
Zhou F et al.	15	54	29	137	11.7%	1.31 [0.77, 2.25]
**Total (95% CI)**		**209**		**1021**	**100.0%**	**1.20 [1.00, 1.44]**
Total events	97		348			

Heterogeneity: Tau² = 0.00; Chi² = 1.07, df = 3 (P = 0.79); I² = 0%
Test for overall effect: Z = 1.92 (P = 0.05)

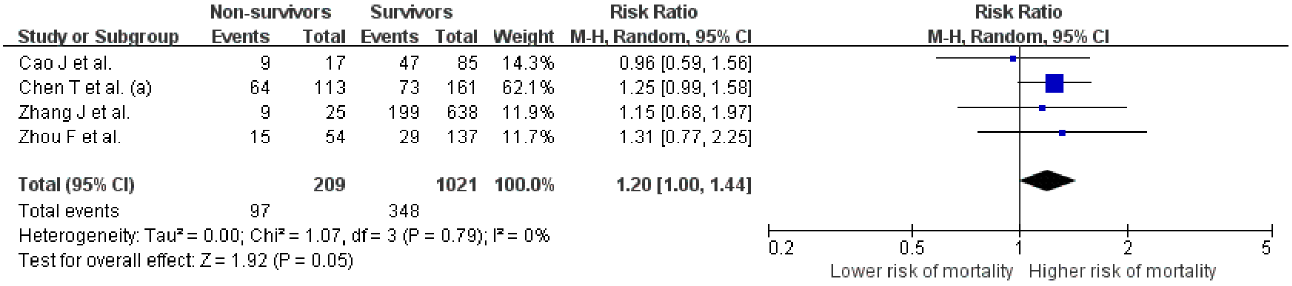

Figure S21. Forest plot of association between symptoms of fatigue and mortality

Study or Subgroup	Non-survivors Events	Non-survivors Total	Survivors Events	Survivors Total	Weight	Risk Ratio M-H, Random, 95% CI
Chen T et al. (a)	31	113	35	161	27.6%	1.26 [0.83, 1.92]
Wang L et al. (b)	15	65	79	274	24.0%	0.80 [0.49, 1.30]
Xu B et al.	27	28	91	117	48.4%	1.24 [1.10, 1.40]
**Total (95% CI)**		**206**		**552**	**100.0%**	**1.12 [0.81, 1.55]**
Total events	73		205			

Heterogeneity: Tau² = 0.05; Chi² = 5.61, df = 2 (P = 0.06); I² = 64%
Test for overall effect: Z = 0.70 (P = 0.48)

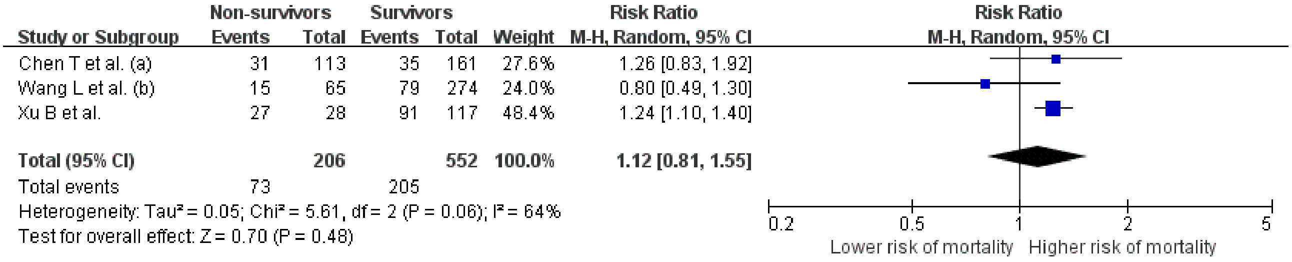

Figure S22. Forest plot of association between symptoms of anorexia and mortality

Study or Subgroup	Non-survivors Events	Non-survivors Total	Survivors Events	Survivors Total	Weight	Risk Ratio M-H, Random, 95% CI
Cao J et al.	3	17	8	85	7.2%	1.88 [0.55, 6.35]
Chen T et al. (a)	27	113	50	161	66.5%	0.77 [0.51, 1.15]
Xu B et al.	6	28	28	117	17.7%	0.90 [0.41, 1.95]
Yao Q et al.	1	12	7	96	2.7%	1.14 [0.15, 8.51]
Zhang J et al.	0	25	61	638	1.4%	0.20 [0.01, 3.14]
Zhou F et al.	2	54	7	137	4.5%	0.72 [0.16, 3.38]
**Total (95% CI)**		**249**		**1234**	**100.0%**	**0.83 [0.60, 1.16]**
Total events	39		161			

Heterogeneity: Tau² = 0.00; Chi² = 3.10, df = 5 (P = 0.68); I² = 0%
Test for overall effect: Z = 1.09 (P = 0.27)

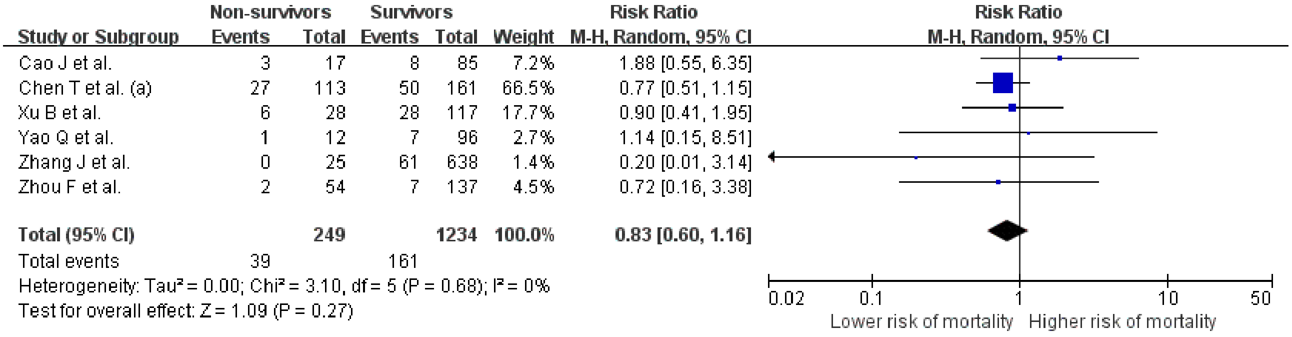

Figure S23. Forest plot of association between symptoms of diarrhea and mortality

Study or Subgroup	Non-survivors Events	Non-survivors Total	Survivors Events	Survivors Total	Weight	Risk Ratio M-H, Random, 95% CI
Cao J et al.	5	17	30	85	18.2%	0.83 [0.38, 1.84]
Chen T et al. (a)	21	113	39	161	50.9%	0.77 [0.48, 1.23]
Xu B et al.	3	28	28	117	9.1%	0.45 [0.15, 1.37]
Zhang J et al.	0	25	63	638	1.5%	0.19 [0.01, 3.04]
Zhou F et al.	8	54	21	137	20.2%	0.97 [0.46, 2.05]
**Total (95% CI)**		**237**		**1138**	**100.0%**	**0.76 [0.54, 1.07]**
Total events	37		181			
Heterogeneity: Tau² = 0.00; Chi² = 2.36, df = 4 (P = 0.67); I² = 0%						
Test for overall effect: Z = 1.59 (P = 0.11)						

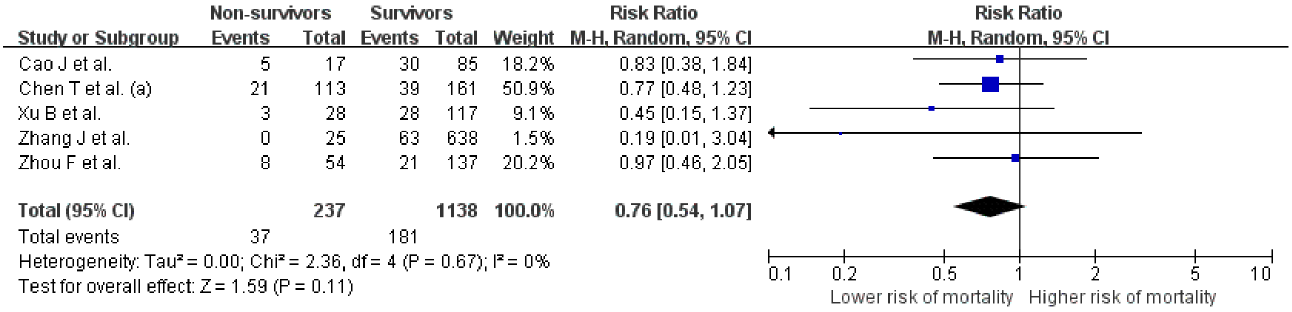

Figure S24. Forest plot of association between symptoms of myalgia and mortality

Study or Subgroup	Non-survivors Events	Non-survivors Total	Survivors Events	Survivors Total	Weight	Risk Ratio M-H, Random, 95% CI
Chen T et al. (a)	10	113	11	161	53.7%	1.30 [0.57, 2.95]
Xu B et al.	5	28	10	117	36.9%	2.09 [0.78, 5.63]
Zhang J et al.	1	25	22	638	9.4%	1.16 [0.16, 8.27]
**Total (95% CI)**		**166**		**916**	**100.0%**	**1.53 [0.84, 2.79]**
Total events	16		43			
Heterogeneity: Tau² = 0.00; Chi² = 0.62, df = 2 (P = 0.73); I² = 0%						
Test for overall effect: Z = 1.38 (P = 0.17)						

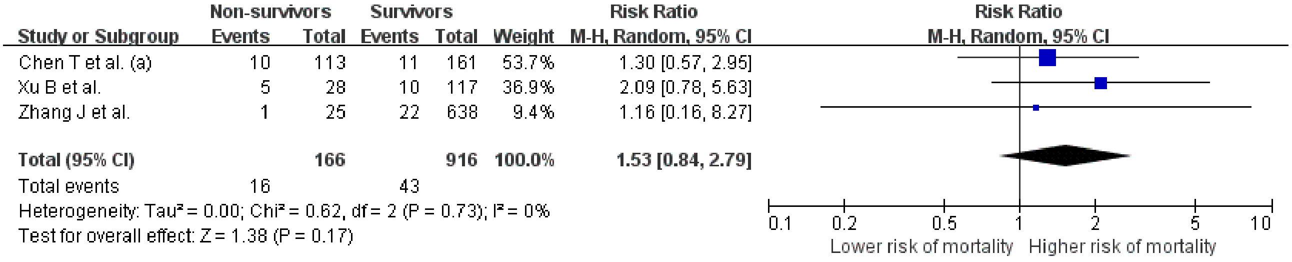

Figure S25. Forest plot of association between symptoms of dizziness and mortality

Study or Subgroup	Non-survivors Events	Non-survivors Total	Survivors Events	Survivors Total	Weight	Risk Ratio M-H, Random, 95% CI
Chen T et al. (a)	8	113	16	161	41.6%	0.71 [0.32, 1.61]
Xu B et al.	7	28	11	117	40.5%	2.66 [1.13, 6.24]
Zhang J et al.	1	25	30	638	18.0%	0.85 [0.12, 5.99]
**Total (95% CI)**		**166**		**916**	**100.0%**	**1.25 [0.46, 3.40]**
Total events	16		57			
Heterogeneity: Tau² = 0.45; Chi² = 5.13, df = 2 (P = 0.08); I² = 61%						
Test for overall effect: Z = 0.44 (P = 0.66)						

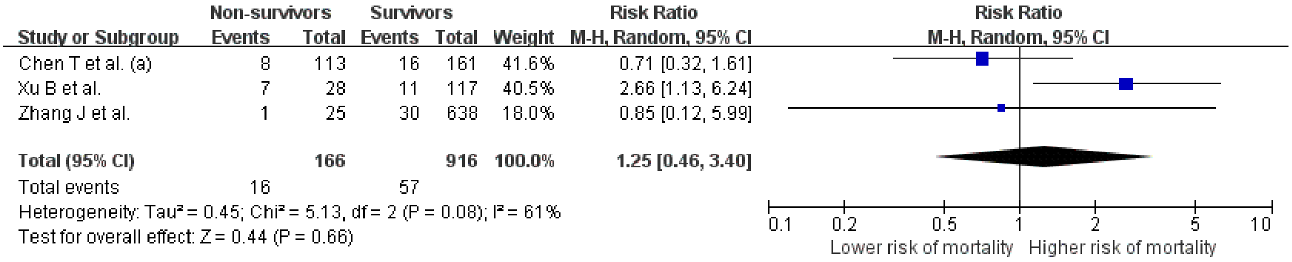

Figure S26. Forest plot of association between symptoms of nausea and mortality

Study or Subgroup	Non-survivors Events	Non-survivors Total	Survivors Events	Survivors Total	Weight	Risk Ratio M-H, Random, 95% CI
Chen T et al. (a)	11	113	20	161	41.8%	0.78 [0.39, 1.57]
Du RH et al.	5	21	12	158	33.2%	3.13 [1.23, 8.01]
Xu B et al.	1	28	5	117	11.7%	0.84 [0.10, 6.87]
Yao Q et al.	0	12	1	96	5.9%	2.49 [0.11, 57.91]
Zhang J et al.	0	25	20	638	7.4%	0.60 [0.04, 9.64]
**Total (95% CI)**		**199**		**1170**	**100.0%**	**1.31 [0.59, 2.93]**
Total events	17		58			
Heterogeneity: Tau² = 0.28; Chi² = 6.24, df = 4 (P = 0.18); I² = 36%						
Test for overall effect: Z = 0.67 (P = 0.51)						

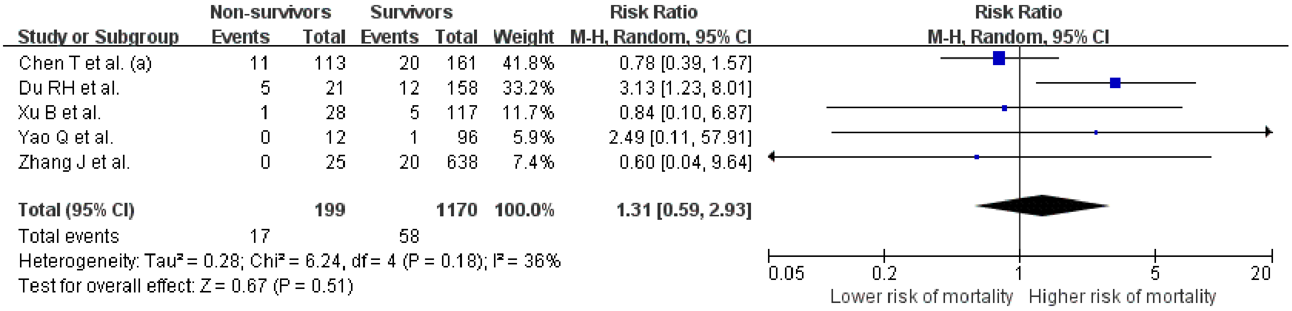

Figure S27. Forest plot of association between symptoms of headache and mortality

Study or Subgroup	Non-survivors Events	Non-survivors Total	Survivors Events	Survivors Total	Weight	Risk Ratio M-H, Random, 95% CI
Chen T et al. (a)	6	113	10	161	72.6%	0.85 [0.32, 2.28]
Yang X et al. (b)	1	32	1	20	9.5%	0.63 [0.04, 9.44]
Zhang J et al.	1	25	16	638	17.9%	1.59 [0.22, 11.56]
**Total (95% CI)**		**170**		**819**	**100.0%**	**0.93 [0.40, 2.14]**
Total events	8		27			

Heterogeneity: Tau² = 0.00; Chi² = 0.40, df = 2 (P = 0.82); I² = 0%
Test for overall effect: Z = 0.18 (P = 0.86)

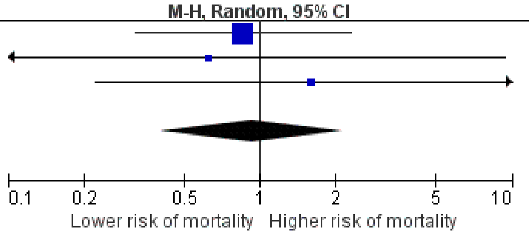

Figure S28. Forest plot of association between symptoms of vomiting and mortality

Study or Subgroup	Non-survivors Events	Non-survivors Total	Survivors Events	Survivors Total	Weight	Risk Ratio M-H, Random, 95% CI
Chen T et al. (a)	4	113	8	161	40.9%	0.71 [0.22, 2.31]
Wang L et al. (b)	3	65	10	274	35.5%	1.26 [0.36, 4.47]
Xu B et al.	2	28	6	117	23.6%	1.39 [0.30, 6.54]
**Total (95% CI)**		**206**		**552**	**100.0%**	**1.02 [0.48, 2.17]**
Total events	9		24			

Heterogeneity: Tau² = 0.00; Chi² = 0.63, df = 2 (P = 0.73); I² = 0%
Test for overall effect: Z = 0.06 (P = 0.95)

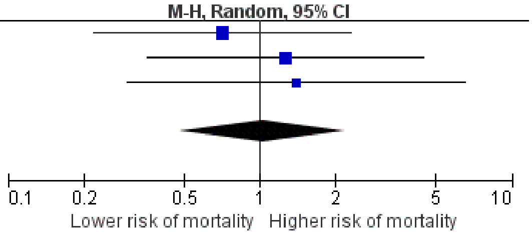

Figure S29. Forest plot of association between symptoms of pharyngalgia and mortality

Study or Subgroup	Non-survivors Events	Non-survivors Total	Survivors Events	Survivors Total	Weight	Risk Ratio M-H, Random, 95% CI
Chen T et al. (a)	4	113	3	161	73.9%	1.90 [0.43, 8.32]
Du RH et al.	0	21	10	158	26.1%	0.34 [0.02, 5.67]
**Total (95% CI)**		**134**		**319**	**100.0%**	**1.22 [0.27, 5.51]**
Total events	4		13			

Heterogeneity: Tau² = 0.23; Chi² = 1.18, df = 1 (P = 0.28); I² = 15%
Test for overall effect: Z = 0.26 (P = 0.80)

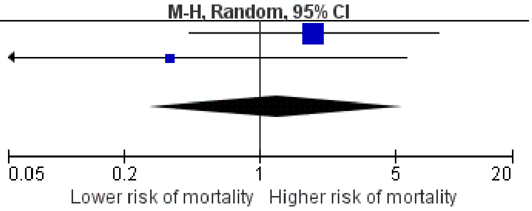

Figure S30. Forest plot of association between symptoms of hemoptysis and mortality

Study or Subgroup	Non-survivors Events	Non-survivors Total	Survivors Events	Survivors Total	Weight	Risk Ratio M-H, Random, 95% CI
Cao J et al.	7	17	3	85	37.5%	11.67 [3.35, 40.65]
Chen T et al. (a)	46	113	0	161	26.2%	132.16 [8.23, 2122.49]
Wang L et al. (b)	3	65	5	274	36.4%	2.53 [0.62, 10.31]
**Total (95% CI)**		**195**		**520**	**100.0%**	**12.62 [1.25, 127.06]**
Total events	56		8			

Heterogeneity: Tau² = 3.30; Chi² = 11.40, df = 2 (P = 0.003); I² = 82%
Test for overall effect: Z = 2.15 (P = 0.03)

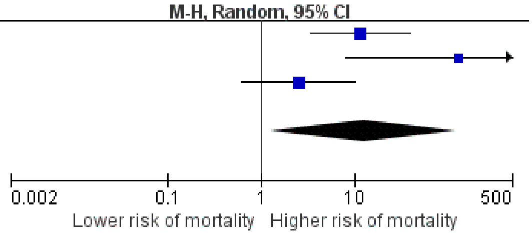

Figure S31. Forest plot of association between shock and mortality

Study or Subgroup	Non-survivors Events	Non-survivors Total	Survivors Events	Survivors Total	Weight	Risk Ratio M-H, Random, 95% CI
Cao J et al.	14	17	3	85	24.9%	23.33 [7.51, 72.45]
Wang L et al. (b)	49	65	94	274	28.6%	2.20 [1.77, 2.72]
Xu B et al.	11	28	9	117	26.8%	5.11 [2.34, 11.12]
Zhou F et al.	27	54	1	137	19.7%	68.50 [9.54, 491.65]
**Total (95% CI)**		**164**		**613**	**100.0%**	**9.78 [2.05, 46.66]**
Total events	101		107			

Heterogeneity: Tau² = 2.21; Chi² = 44.99, df = 3 (P < 0.00001); I² = 93%
Test for overall effect: Z = 2.86 (P = 0.004)

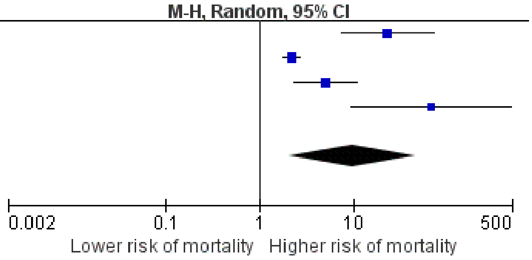

Figure S32. Forest plot of association between acute infection and mortality

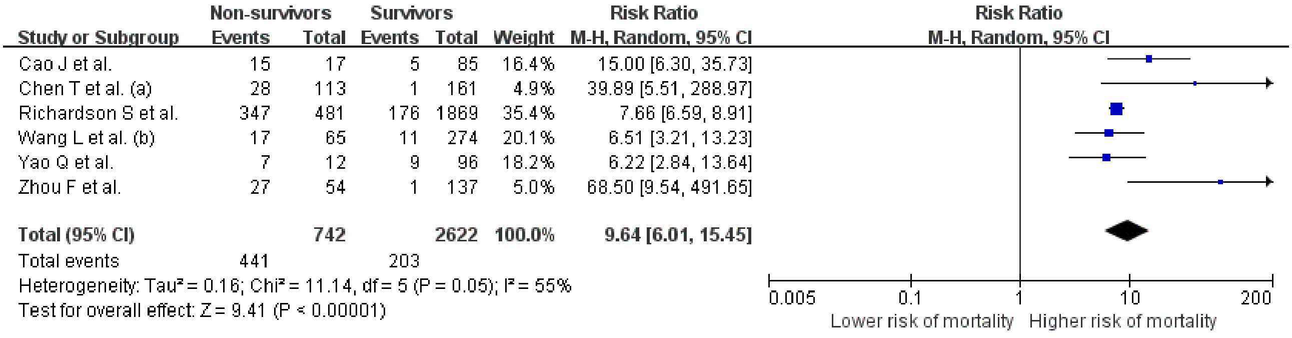

Figure S33. Forest plot of association between acute kidney injury and mortality

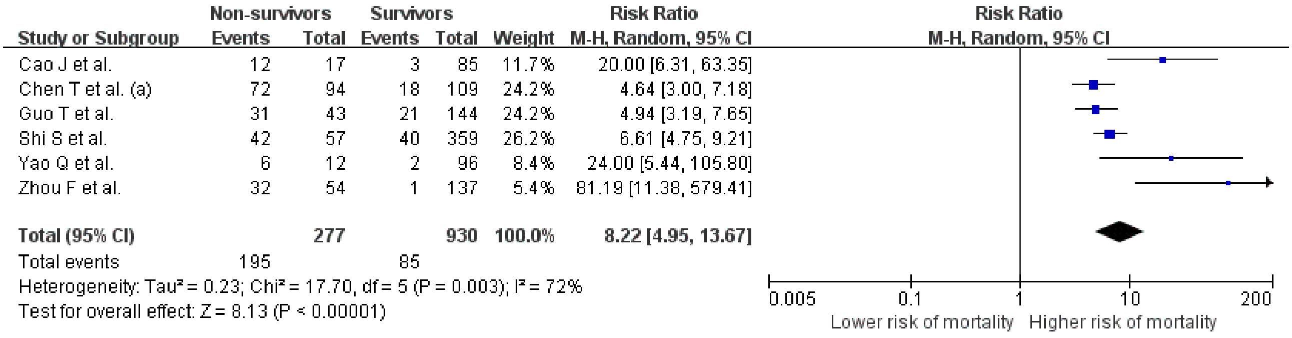

Figure S34. Forest plot of association between acute cardiac injury and mortality

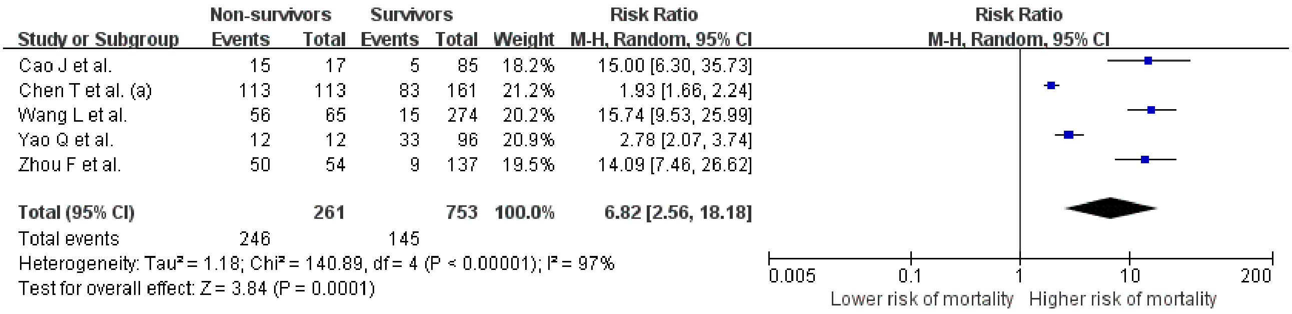

Figure S35. Forest plot of association between acute respiratory distress syndrome and mortality

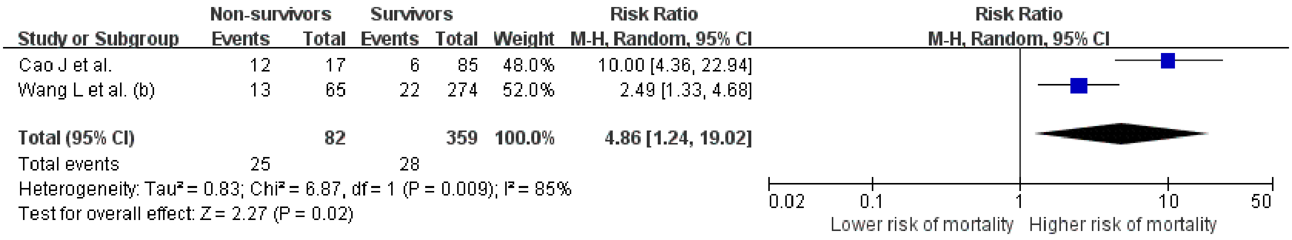

Figure S36. Forest plot of association between arrhythmia and mortality

Study or Subgroup	Non-survivors Events	Non-survivors Total	Survivors Events	Survivors Total	Weight	Risk Ratio M-H, Random, 95% CI
Chen T et al. (a)	41	83	3	94	14.6%	15.48 [4.98, 48.13]
Peng YD et al.	13	17	27	95	29.6%	2.69 [1.78, 4.07]
Wang L et al. (b)	25	65	33	274	28.9%	3.19 [2.05, 4.98]
Zhou F et al.	28	54	16	137	26.9%	4.44 [2.62, 7.52]
**Total (95% CI)**		**219**		**600**	**100.0%**	**4.18 [2.37, 7.36]**
Total events	107		79			

Heterogeneity: Tau² = 0.24; Chi² = 12.58, df = 3 (P = 0.006); I² = 76%
Test for overall effect: Z = 4.95 (P < 0.00001)

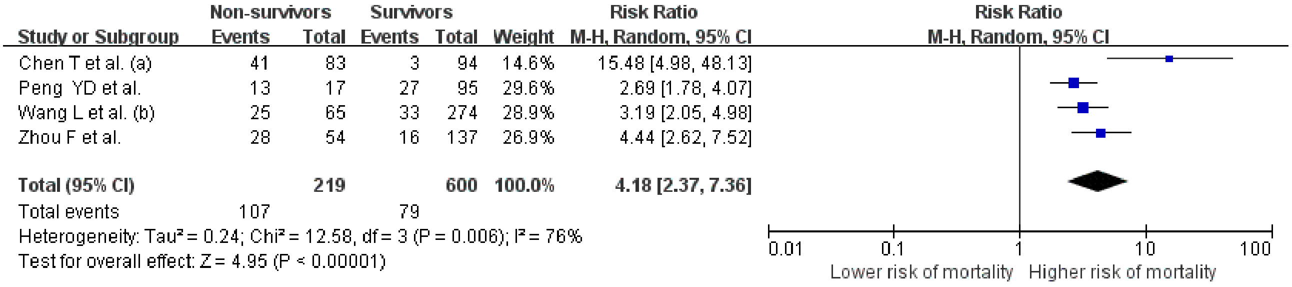

Figure S37. Forest plot of association between heart failure and mortality

Study or Subgroup	Non-survivors Events	Non-survivors Total	Survivors Events	Survivors Total	Weight	Risk Ratio M-H, Random, 95% CI
Cao J et al.	13	17	21	85	21.8%	3.10 [1.96, 4.88]
Chen T et al. (a)	10	113	3	161	17.8%	4.75 [1.34, 16.87]
Richardson S et al.	53	551	3	2046	18.4%	65.60 [20.58, 209.13]
Wang L et al. (b)	22	65	74	274	22.0%	1.25 [0.85, 1.85]
Yang X et al. (b)	9	32	6	20	20.0%	0.94 [0.39, 2.24]
**Total (95% CI)**		**778**		**2586**	**100.0%**	**3.78 [1.18, 12.14]**
Total events	107		107			

Heterogeneity: Tau² = 1.57; Chi² = 59.25, df = 4 (P < 0.00001); I² = 93%
Test for overall effect: Z = 2.24 (P = 0.03)

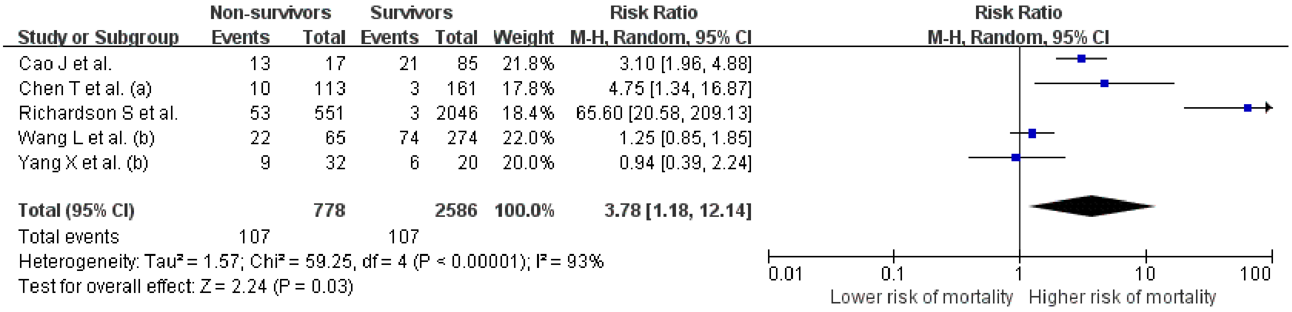

Figure S38. Forest plot of association between acute liver injury and mortality

Study or Subgroup	Non-survivors Events	Non-survivors Total	Survivors Events	Survivors Total	Weight	Risk Ratio M-H, Random, 95% CI
Chen T et al. (a)	113	113	66	161	41.3%	2.43 [2.02, 2.92]
Yao Q et al.	11	12	24	96	19.1%	3.67 [2.49, 5.40]
Zhou F et al.	54	54	58	137	39.6%	2.34 [1.92, 2.84]
**Total (95% CI)**		**179**		**394**	**100.0%**	**2.59 [2.11, 3.17]**
Total events	178		148			

Heterogeneity: Tau² = 0.02; Chi² = 4.37, df = 2 (P = 0.11); I² = 54%
Test for overall effect: Z = 9.20 (P < 0.00001)

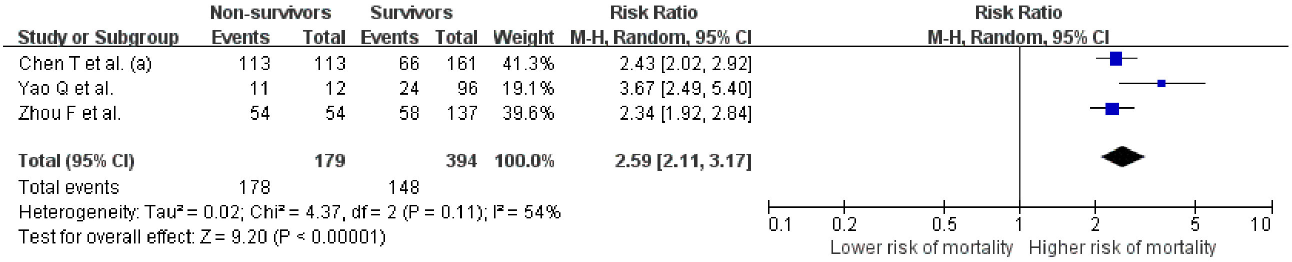

Figure S39. Forest plot of association between sepsis and mortality

Study or Subgroup	Non-survivors Events	Non-survivors Total	Survivors Events	Survivors Total	Weight	Risk Ratio M-H, Random, 95% CI
Cao J et al.	5	17	1	85	17.3%	25.00 [3.11, 200.70]
Chen T et al. (a)	3	113	0	161	8.6%	9.95 [0.52, 190.72]
Richardson S et al.	78	553	3	2081	57.0%	97.84 [31.00, 308.79]
Yao Q et al.	1	12	0	96	7.6%	22.38 [0.96, 521.23]
Zhou F et al.	10	54	0	137	9.5%	52.69 [3.14, 883.78]
**Total (95% CI)**		**749**		**2560**	**100.0%**	**53.46 [22.45, 127.28]**
Total events	97		4			

Heterogeneity: Tau² = 0.00; Chi² = 3.27, df = 4 (P = 0.51); I² = 0%
Test for overall effect: Z = 8.99 (P < 0.00001)

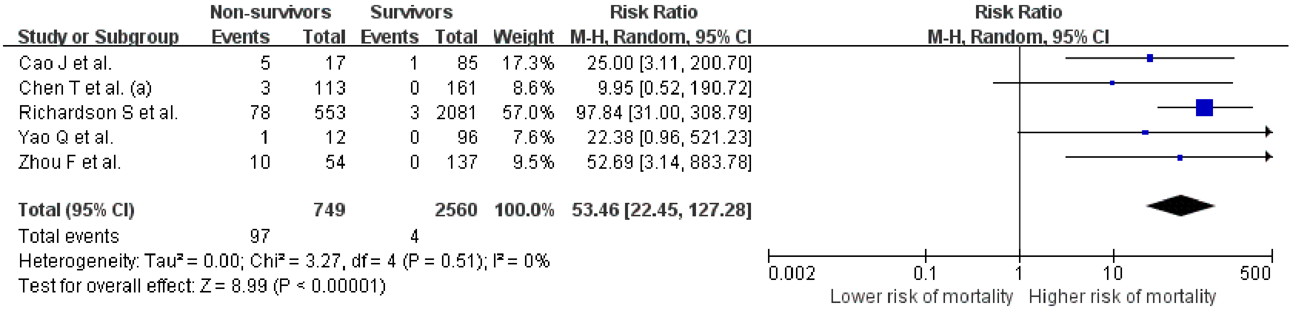

Figure S40. Forest plot of association between renal replacement therapy and mortality

Study or Subgroup	Non-survivors Events	Non-survivors Total	Survivors Events	Survivors Total	Weight	Risk Ratio M-H, Random, 95% CI
Cao J et al.	12	17	2	85	4.9%	30.00 [7.37, 122.08]
Chen T et al. (a)	17	113	0	161	1.2%	49.74 [3.02, 818.64]
Richardson S et al.	282	553	38	2081	90.3%	27.93 [20.17, 38.67]
Wu C et al.	5	44	0	157	1.2%	38.62 [2.18, 685.32]
Xu B et al.	9	28	0	117	1.2%	77.31 [4.63, 1290.11]
Yao Q et al.	10	12	0	96	1.2%	156.69 [9.75, 2519.04]
**Total (95% CI)**		**767**		**2697**	**100.0%**	**29.30 [21.50, 39.92]**
Total events	335		40			

Heterogeneity: Tau² = 0.00; Chi² = 2.14, df = 5 (P = 0.83); I² = 0%
Test for overall effect: Z = 21.40 (P < 0.00001)

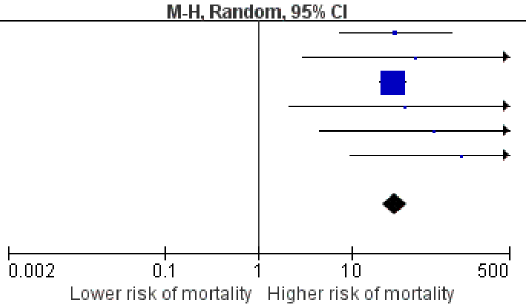

Figure S41. Forest plot of association between invasive mechanical ventilation and mortality

Study or Subgroup	Non-survivors Events	Non-survivors Total	Survivors Events	Survivors Total	Weight	Risk Ratio M-H, Random, 95% CI
Cao J et al.	3	17	2	85	2.1%	7.50 [1.35, 41.54]
Chen T et al. (a)	76	113	26	161	44.6%	4.16 [2.86, 6.06]
Wu C et al.	38	44	23	157	40.1%	5.90 [3.97, 8.75]
Xu B et al.	13	28	9	117	11.3%	6.04 [2.87, 12.69]
Yao Q et al.	2	12	2	96	1.8%	8.00 [1.24, 51.69]
**Total (95% CI)**		**214**		**616**	**100.0%**	**5.12 [3.98, 6.57]**
Total events	132		62			

Heterogeneity: Tau² = 0.00; Chi² = 2.33, df = 4 (P = 0.67); I² = 0%
Test for overall effect: Z = 12.78 (P < 0.00001)

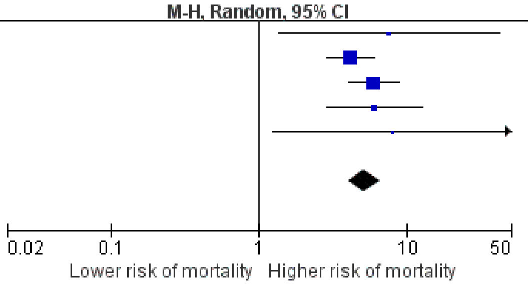

Figure S42. Forest plot of association between noninvasive ventilation and mortality

Study or Subgroup	Non-survivors Events	Non-survivors Total	Survivors Events	Survivors Total	Weight	Risk Ratio M-H, Random, 95% CI
Chen T et al. (a)	77	113	8	161	46.0%	13.71 [6.90, 27.26]
Xu B et al.	2	28	1	117	5.6%	8.36 [0.79, 88.93]
Yao Q et al.	0	12	4	96	3.9%	0.83 [0.05, 14.53]
Zhou F et al.	33	54	8	137	44.4%	10.47 [5.17, 21.19]
**Total (95% CI)**		**207**		**511**	**100.0%**	**10.60 [5.97, 18.82]**
Total events	112		21			

Heterogeneity: Tau² = 0.06; Chi² = 3.61, df = 3 (P = 0.31); I² = 17%
Test for overall effect: Z = 8.06 (P < 0.00001)

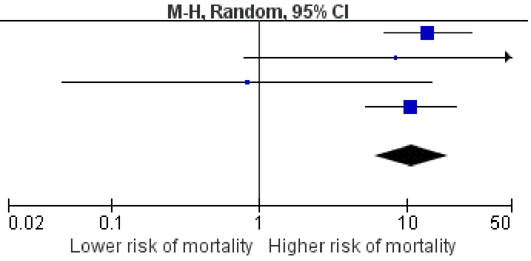

Figure S43. Forest plot of association between high flow nasal cannula and mortality

Study or Subgroup	Higher risk of mortality Events	Higher risk of mortality Total	Survivors Events	Survivors Total	Weight	Risk Ratio M-H, Random, 95% CI
Cao J et al.	1	17	2	85	48.1%	2.50 [0.24, 26.04]
Chen T et al. (a)	1	113	0	161	25.9%	4.26 [0.18, 103.71]
Wu C et al.	1	44	0	157	26.0%	10.53 [0.44, 254.16]
**Total (95% CI)**		**174**		**403**	**100.0%**	**4.17 [0.82, 21.19]**
Total events	3		2			

Heterogeneity: Tau² = 0.00; Chi² = 0.51, df = 2 (P = 0.77); I² = 0%
Test for overall effect: Z = 1.72 (P = 0.08)

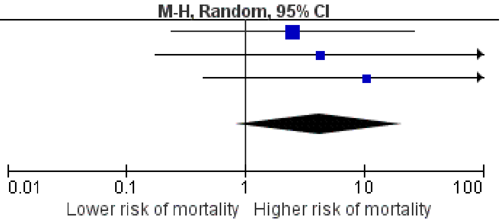

Figure S44. Forest plot of association between ECMO and mortality

Study or Subgroup	Non-survivors Events	Non-survivors Total	Survivors Events	Survivors Total	Weight	Risk Ratio M-H, Random, 95% CI
Cao J et al.	11	17	40	85	18.2%	1.38 [0.91, 2.09]
Chen T et al. (a)	99	113	118	161	23.7%	1.20 [1.06, 1.34]
Wu C et al.	23	44	39	157	18.8%	2.10 [1.42, 3.11]
Xu B et al.	24	28	63	117	22.2%	1.59 [1.27, 2.00]
Yao Q et al.	10	12	20	96	17.2%	4.00 [2.51, 6.37]
**Total (95% CI)**		**214**		**616**	**100.0%**	**1.79 [1.25, 2.55]**
Total events	167		280			

Heterogeneity: $Tau^2 = 0.13$; $Chi^2 = 33.13$, df = 4 (P < 0.00001); $I^2 = 88\%$
Test for overall effect: Z = 3.21 (P = 0.001)

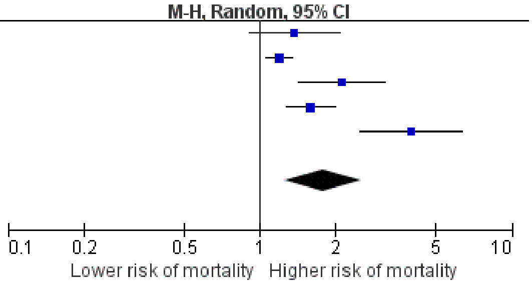

Figure S45. Forest plot of association between corticosteroid therapy and mortality

Study or Subgroup	Non-survivors Events	Non-survivors Total	Survivors Events	Survivors Total	Weight	Risk Ratio M-H, Random, 95% CI
Cao J et al.	17	17	84	85	21.6%	0.99 [0.91, 1.08]
Chen T et al. (a)	105	113	144	161	21.9%	1.04 [0.97, 1.12]
Wu C et al.	43	44	153	157	22.4%	1.00 [0.95, 1.06]
Xu B et al.	28	28	92	117	20.8%	1.25 [1.13, 1.39]
Yao Q et al.	12	12	36	96	13.3%	2.56 [1.93, 3.38]
**Total (95% CI)**		**214**		**616**	**100.0%**	**1.20 [1.02, 1.40]**
Total events	205		509			

Heterogeneity: $Tau^2 = 0.03$; $Chi^2 = 63.17$, df = 4 (P < 0.00001); $I^2 = 94\%$
Test for overall effect: Z = 2.23 (P = 0.03)

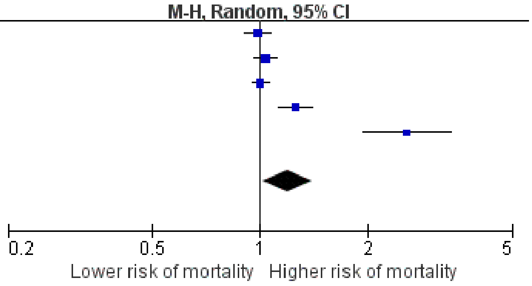

Figure S46. Forest plot of association between antibiotic therapy and mortality

Study or Subgroup	Non-survivors Events	Non-survivors Total	Control Events	Control Total	Weight	Risk Ratio M-H, Random, 95% CI
Cao J et al.	0	17	5	85	7.2%	0.43 [0.03, 7.51]
Chen T et al. (a)	44	113	59	161	25.8%	1.06 [0.78, 1.45]
Xu B et al.	15	28	54	117	25.2%	1.16 [0.78, 1.73]
Yao Q et al.	3	12	9	96	18.3%	2.67 [0.84, 8.51]
Zhou F et al.	36	54	10	137	23.5%	9.13 [4.88, 17.08]
**Total (95% CI)**		**224**		**596**	**100.0%**	**2.00 [0.82, 4.88]**
Total events	98		137			

Heterogeneity: $Tau^2 = 0.78$; $Chi^2 = 41.60$, df = 4 (P < 0.00001); $I^2 = 90\%$
Test for overall effect: Z = 1.52 (P = 0.13)

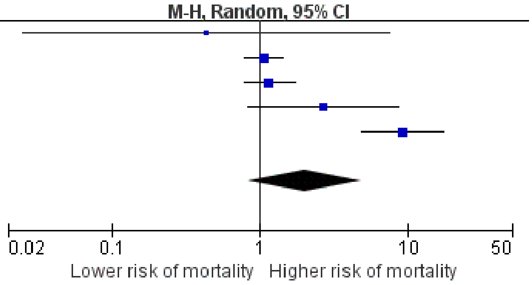

Figure S47. Forest plot of association between immunoglobulin therapy and mortality

Study or Subgroup	Non-survivors Events	Non-survivors Total	Survivors Events	Survivors Total	Weight	Risk Ratio M-H, Random, 95% CI
Cao J et al.	17	17	83	85	27.2%	1.00 [0.92, 1.09]
Chen T et al. (a)	89	113	147	161	26.6%	0.86 [0.78, 0.96]
Wu C et al.	25	44	145	157	19.7%	0.62 [0.47, 0.80]
Yao Q et al.	12	12	96	96	26.5%	1.00 [0.90, 1.12]
**Total (95% CI)**		**186**		**499**	**100.0%**	**0.87 [0.71, 1.08]**
Total events	143		471			

Heterogeneity: $Tau^2 = 0.04$; $Chi^2 = 36.17$, df = 3 (P < 0.00001); $I^2 = 92\%$
Test for overall effect: Z = 1.26 (P = 0.21)

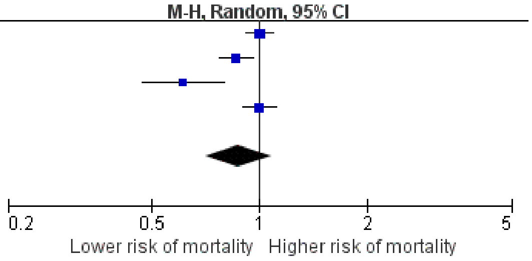

Figure S48. Forest plot of association between antiviral therapy and mortality

Study or Subgroup	Non-survivors Mean	SD	Total	Survivors Mean	SD	Total	Weight	Mean Difference IV, Random, 95% CI
Chen T et al. (a)	10	5.56	113	5	1.94	161	24.8%	5.00 [3.93, 6.07]
Chen T et al. (b)	11.7	17.62	19	7.2	9.26	36	1.3%	4.50 [-3.98, 12.98]
Du RH et al.	8.93	6.6	21	5.4	2.62	158	9.1%	3.53 [0.68, 6.38]
Wang L et al. (b)	9.04	6.05	65	5.82	2.5	274	19.5%	3.22 [1.72, 4.72]
Wu C et al.	8.65	4.25	44	5.19	2.86	117	21.1%	3.46 [2.10, 4.82]
Xu B et al.	7.16	4.18	28	5.34	2.44	117	18.3%	1.82 [0.21, 3.43]
Yao Q et al.	9.67	4.47	12	5.91	5.12	13	5.9%	3.76 [-0.00, 7.52]
**Total (95% CI)**			**302**			**876**	**100.0%**	**3.53 [2.53, 4.54]**

Heterogeneity: Tau² = 0.76; Chi² = 11.48, df = 6 (P = 0.07); I² = 48%
Test for overall effect: Z = 6.90 (P < 0.00001)

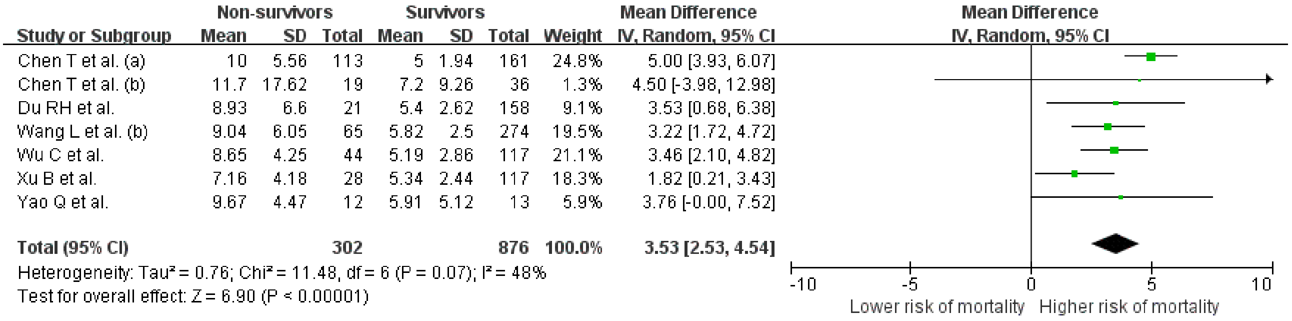

Figure S49. Forest plot of mean difference in white blood cell count between nonsurvivors and survivors

Study or Subgroup	Non-survivors Mean	SD	Total	Survivors Mean	SD	Total	Weight	Mean Difference IV, Random, 95% CI
Chen T et al. (a)	9.03	5.48	113	3.37	1.57	161	22.3%	5.66 [4.62, 6.70]
Chen T et al. (b)	9.5	15.22	19	6.1	9.26	36	2.1%	3.40 [-4.08, 10.88]
Du RH et al.	7.4	6.76	21	4.2	2.62	158	9.6%	3.20 [0.28, 6.12]
Wang L et al. (b)	7.91	5.6	65	4.2	2.49	274	19.4%	3.71 [2.32, 5.10]
Wu C et al.	7.73	4.18	44	3.55	2.65	117	20.0%	4.18 [2.85, 5.51]
Xu B et al.	5.93	3.96	28	3.69	2.21	117	18.3%	2.24 [0.72, 3.76]
Yao Q et al.	6.53	5.26	12	3.46	2.56	13	8.2%	3.07 [-0.22, 6.36]
**Total (95% CI)**			**302**			**876**	**100.0%**	**3.86 [2.74, 4.99]**

Heterogeneity: Tau² = 1.19; Chi² = 15.39, df = 6 (P = 0.02); I² = 61%
Test for overall effect: Z = 6.73 (P < 0.00001)

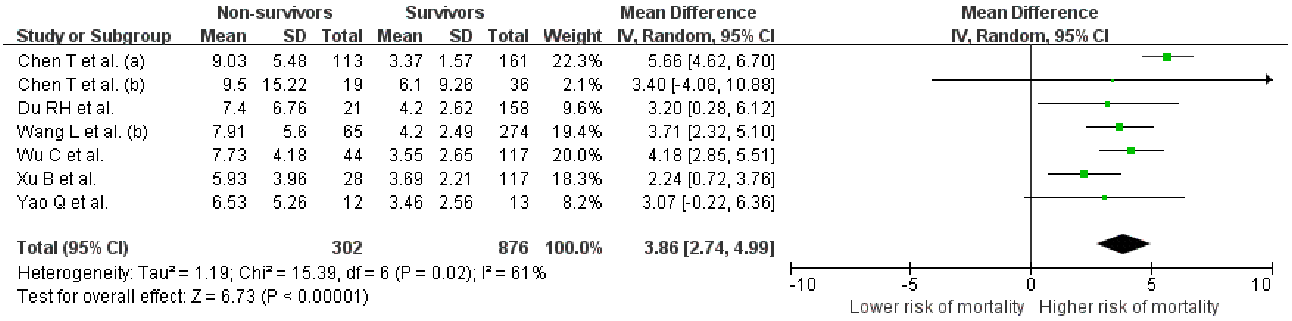

Figure S50. Forest plot of mean difference in neutrophil count between nonsurvivors and survivors

Study or Subgroup	Non-survivors Mean	SD	Total	Survivors Mean	SD	Total	Weight	Mean Difference IV, Random, 95% CI
Chen T et al. (a)	0.57	0.23	113	1.03	0.52	161	20.6%	-0.46 [-0.55, -0.37]
Chen T et al. (b)	2.33	4.81	19	0.93	1.54	36	0.3%	1.40 [-0.82, 3.62]
Du RH et al.	0.67	0.24	21	0.83	0.37	158	19.3%	-0.16 [-0.28, -0.04]
Wang L et al. (b)	0.6	0.34	65	1.01	0.51	274	20.1%	-0.41 [-0.51, -0.31]
Wu C et al.	0.6	0.2	44	1.08	0.55	117	19.4%	-0.48 [-0.60, -0.36]
Xu B et al.	0.61	0.48	28	0.98	0.54	117	14.6%	-0.37 [-0.57, -0.17]
Yao Q et al.	0.99	0.8	12	0.79	0.26	13	5.6%	0.20 [-0.27, 0.67]
**Total (95% CI)**			**302**			**876**	**100.0%**	**-0.34 [-0.47, -0.21]**

Heterogeneity: Tau² = 0.02; Chi² = 27.80, df = 6 (P = 0.0001); I² = 78%
Test for overall effect: Z = 5.17 (P < 0.00001)

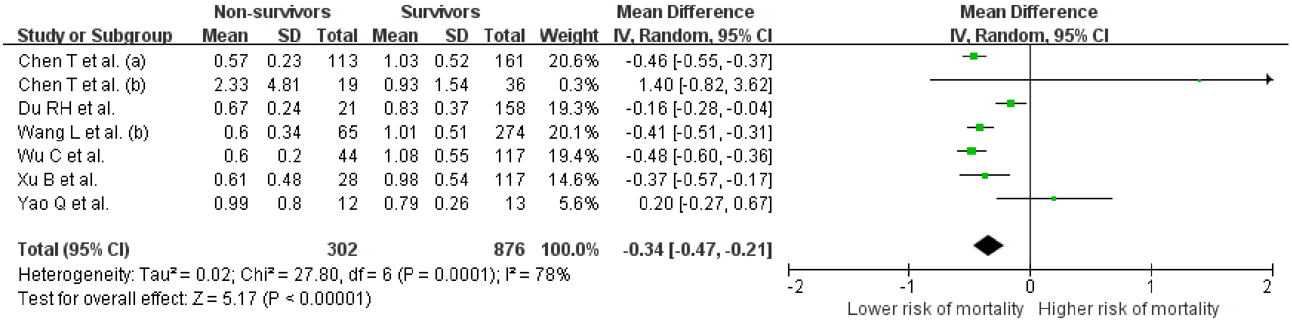

Figure S51. Forest plot of mean difference in lymphocyte count between nonsurvivors and survivors

Study or Subgroup	Non-survivors Mean	SD	Total	Survivors Mean	SD	Total	Weight	Mean Difference IV, Random, 95% CI
Chen T et al. (a)	0.4	0.3	113	0.4	0.15	161	25.9%	0.00 [-0.06, 0.06]
Wang L et al. (b)	0.34	0.2	65	0.45	0.24	274	27.1%	-0.11 [-0.17, -0.05]
Wu C et al.	0.3	0.19	44	0.34	0.14	117	25.4%	-0.04 [-0.10, 0.02]
Xu B et al.	0.32	0.18	28	0.41	0.19	117	21.5%	-0.09 [-0.17, -0.01]
**Total (95% CI)**			**250**			**669**	**100.0%**	**-0.06 [-0.11, -0.01]**

Heterogeneity: Tau² = 0.00; Chi² = 7.89, df = 3 (P = 0.05); I² = 62%
Test for overall effect: Z = 2.29 (P = 0.02)

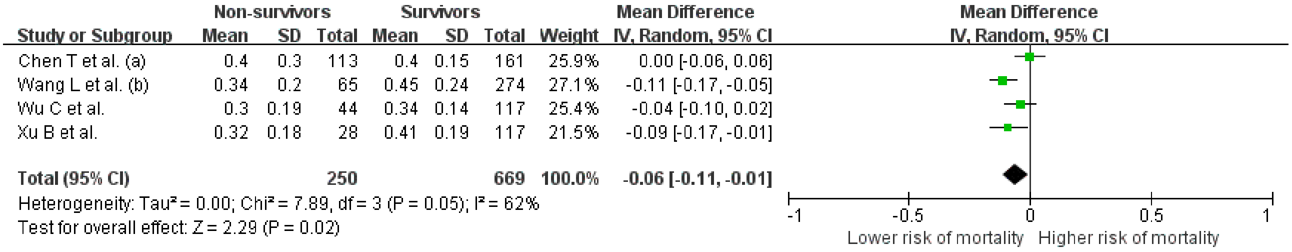

Figure S52. Forest plot of mean difference in monocyte count between nonsurvivors and survivors

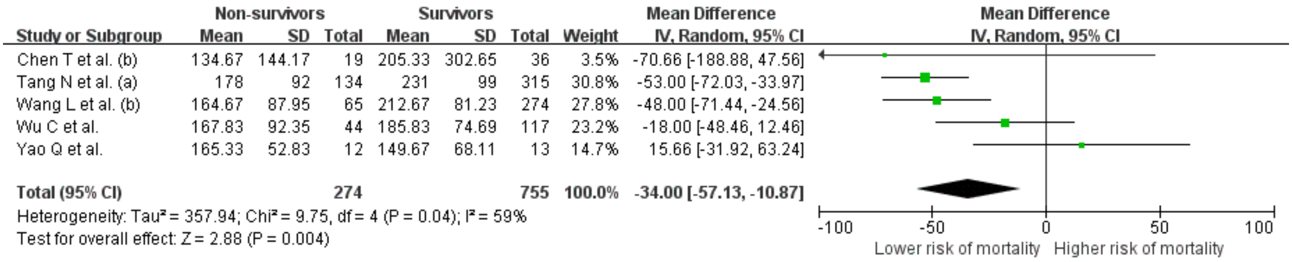

Figure S53. Forest plot of mean difference in platelet count between nonsurvivors and survivors

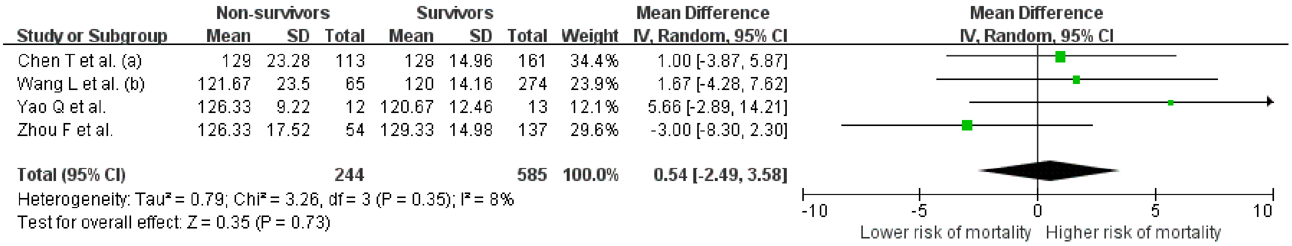

Figure S54. Forest plot of mean difference in hemoglobin between nonsurvivors and survivors

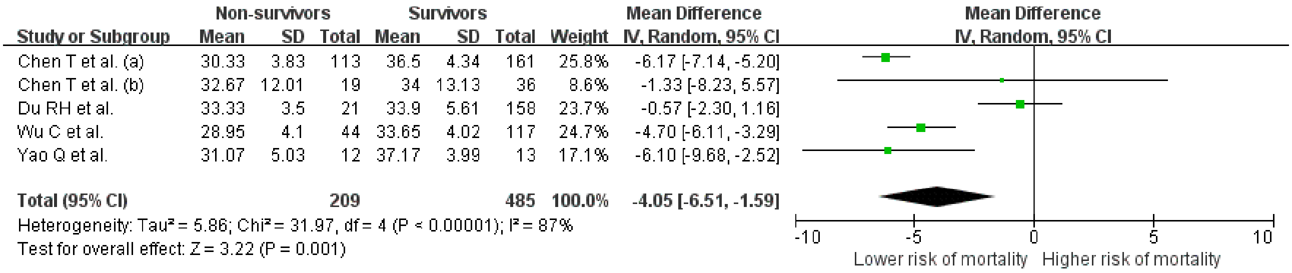

Figure S55. Forest plot of mean difference in albumin between nonsurvivors and survivors

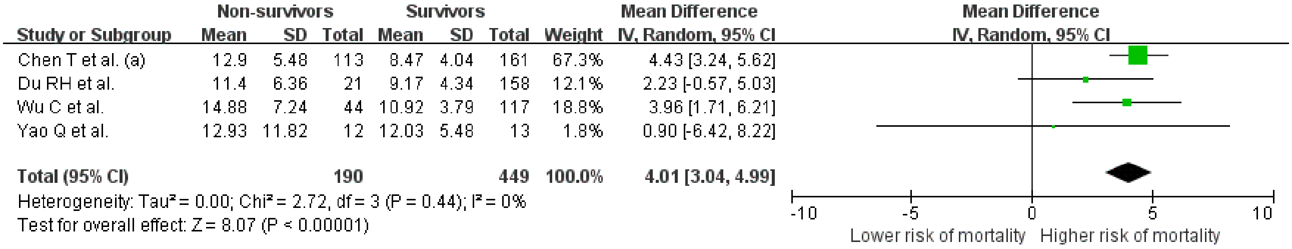

Figure S56. Forest plot of mean difference in total bilirubin between nonsurvivors and survivors

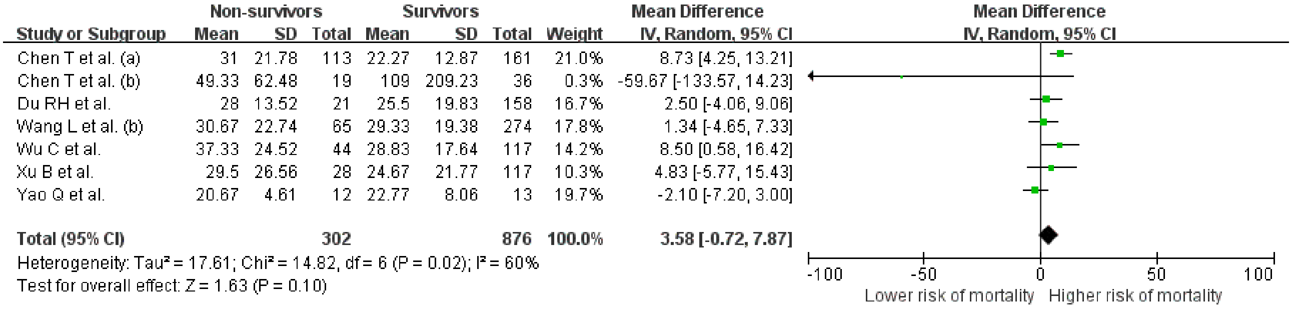

Figure S57. Forest plot of mean difference in ALT between nonsurvivors and survivors

Study or Subgroup	Non-survivors Mean	SD	Total	Survivors Mean	SD	Total	Weight	Mean Difference IV, Random, 95% CI
Chen T et al. (a)	47.67	27.03	113	26.1	9.95	161	25.4%	21.57 [16.35, 26.79]
Chen T et al. (b)	88	120.15	19	94.67	145.92	36	0.5%	-6.67 [-78.72, 65.38]
Du RH et al.	42.83	27.43	21	29.5	17.21	158	12.2%	13.33 [1.30, 25.36]
Wang L et al. (b)	47	28.81	65	31.33	15.65	274	20.6%	15.67 [8.43, 22.91]
Wu C et al.	39.67	16.86	44	30.83	10.88	117	25.0%	8.84 [3.48, 14.20]
Xu B et al.	40	24.61	28	25.67	12.76	117	16.3%	14.33 [4.93, 23.73]
**Total (95% CI)**			**290**			**863**	**100.0%**	**14.84 [9.56, 20.11]**

Heterogeneity: Tau² = 21.44; Chi² = 11.64, df = 5 (P = 0.04); I² = 57%
Test for overall effect: Z = 5.52 (P < 0.00001)

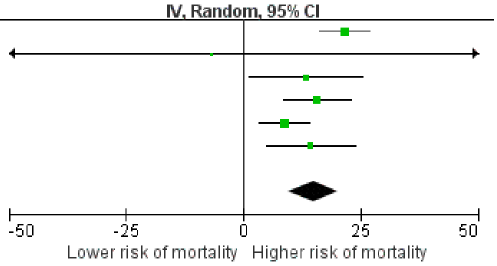

Figure S58. Forest plot of mean difference in AST between nonsurvivors and survivors

Study or Subgroup	Non-survivors Mean	SD	Total	Survivors Mean	SD	Total	Weight	Mean Difference IV, Random, 95% CI
Chen T et al. (a)	89.33	36.05	113	68	22.44	161	21.4%	21.33 [13.83, 28.83]
Chen T et al. (b)	328	579.9	19	270.33	500.31	36	0.1%	57.67 [-250.06, 365.40]
Du RH et al.	90	38.96	21	66.67	18.7	158	12.0%	23.33 [6.41, 40.25]
Wang L et al. (b)	83	44.73	65	60	16.4	274	17.5%	23.00 [11.95, 34.05]
Wu C et al.	72.38	22.42	44	69.07	17.71	117	21.6%	3.31 [-4.05, 10.67]
Xu B et al.	89.82	24.84	28	70.5	21.32	117	18.7%	19.32 [9.34, 29.30]
Yao Q et al.	91.93	30.11	12	66.2	25.42	13	8.7%	25.73 [3.79, 47.67]
**Total (95% CI)**			**302**			**876**	**100.0%**	**18.01 [10.09, 25.92]**

Heterogeneity: Tau² = 61.46; Chi² = 16.87, df = 6 (P = 0.010); I² = 64%
Test for overall effect: Z = 4.46 (P < 0.00001)

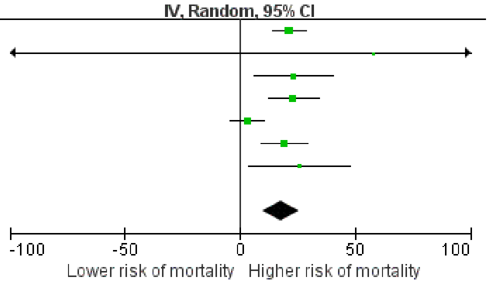

Figure S59. Forest plot of mean difference in creatinine between nonsurvivors and survivors

Study or Subgroup	Non-survivors Mean	SD	Total	Survivors Mean	SD	Total	Weight	Mean Difference IV, Random, 95% CI
Chen T et al. (a)	8.9	5.18	113	4.03	1.57	161	53.5%	4.87 [3.88, 5.86]
Xu B et al.	7.67	3.77	28	4.81	1.87	117	46.5%	2.86 [1.42, 4.30]
**Total (95% CI)**			**141**			**278**	**100.0%**	**3.94 [1.97, 5.90]**

Heterogeneity: Tau² = 1.62; Chi² = 5.11, df = 1 (P = 0.02); I² = 80%
Test for overall effect: Z = 3.93 (P < 0.0001)

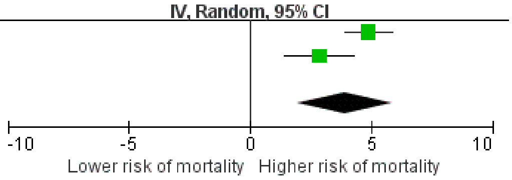

Figure S60. Forest plot of mean difference in blood urea nitrogen between nonsurvivors and survivors

Study or Subgroup	Non-survivors Mean	SD	Total	Survivors Mean	SD	Total	Weight	Mean Difference IV, Random, 95% CI
Wang L et al. (b)	9.7	6.22	65	5.33	2.31	274	42.4%	4.37 [2.83, 5.91]
Wu C et al.	7.08	2.8	44	4.37	1.5	117	57.6%	2.71 [1.84, 3.58]
**Total (95% CI)**			**109**			**391**	**100.0%**	**3.41 [1.81, 5.02]**

Heterogeneity: Tau² = 0.97; Chi² = 3.39, df = 1 (P = 0.07); I² = 71%
Test for overall effect: Z = 4.16 (P < 0.0001)

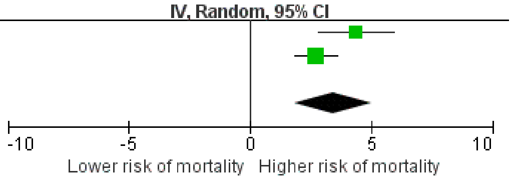

Figure S61. Forest plot of mean difference in urea between nonsurvivors and survivors

Study or Subgroup	Non-survivors Mean	SD	Total	Survivors Mean	SD	Total	Weight	Mean Difference IV, Random, 95% CI
Du RH et al.	14.17	3.18	21	13.77	2.09	158	3.0%	0.40 [-1.00, 1.80]
Tang N et al. (a)	16.5	8.4	134	14.6	2.1	315	2.9%	1.90 [0.46, 3.34]
Wang L et al. (b)	12.97	1.67	65	12.07	0.75	274	34.3%	0.90 [0.48, 1.32]
Wu C et al.	11.72	1.03	44	10.73	1.05	117	46.0%	0.99 [0.63, 1.35]
Xu B et al.	13.65	1.68	28	12.57	1.13	117	13.8%	1.08 [0.42, 1.74]
**Total (95% CI)**			**292**			**981**	**100.0%**	**0.98 [0.74, 1.22]**

Heterogeneity: Tau² = 0.00; Chi² = 2.46, df = 4 (P = 0.65); I² = 0%
Test for overall effect: Z = 7.89 (P < 0.00001)

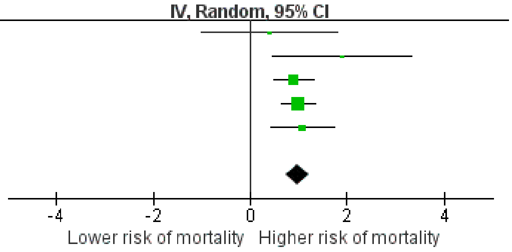

Figure S62. Forest plot of mean difference in prothrombin time between nonsurvivors and survivors

Study or Subgroup	Non-survivors Mean	SD	Total	Survivors Mean	SD	Total	Weight	Mean Difference IV, Random, 95% CI
Chen T et al. (a)	41.03	8.49	113	40.63	5.31	161	26.2%	0.40 [-1.37, 2.17]
Du RH et al.	36.7	8.51	21	35.1	6.14	158	19.3%	1.60 [-2.16, 5.36]
Wang L et al. (b)	29.43	3.34	65	28.37	4.17	274	28.2%	1.06 [0.11, 2.01]
Wu C et al.	24.9	4.67	44	29.38	5.48	117	26.4%	-4.48 [-6.18, -2.78]
**Total (95% CI)**			**243**			**710**	**100.0%**	**-0.47 [-3.31, 2.37]**

Heterogeneity: Tau² = 7.23; Chi² = 32.31, df = 3 (P < 0.00001); I² = 91%
Test for overall effect: Z = 0.32 (P = 0.75)

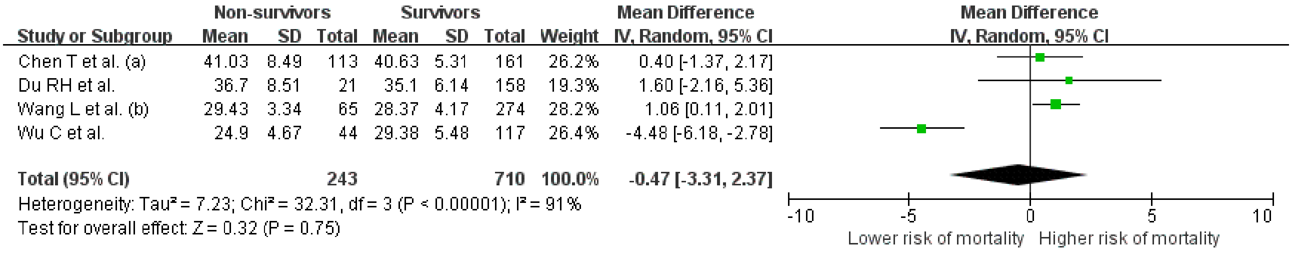

Figure S63. Forest plot of mean difference in APTT between nonsurvivors and survivors

Study or Subgroup	Non-survivors Mean	SD	Total	Survivors Mean	SD	Total	Weight	Mean Difference IV, Random, 95% CI
Chen T et al. (a)	116.83	74.57	113	30.23	35.23	161	16.5%	86.60 [71.81, 101.39]
Chen T et al. (b)	146.33	217.86	19	70.33	115.04	36	5.2%	76.00 [-28.92, 180.92]
Du RH et al.	76.6	53.74	21	48.77	53.64	158	15.3%	27.83 [3.37, 52.29]
Wang L et al. (b)	116.1	97.42	65	46.63	51.2	274	15.3%	69.47 [45.02, 93.92]
Wu C et al.	98.47	88.48	44	29.28	38.39	117	15.0%	69.19 [42.14, 96.24]
Xu B et al.	88.65	54.54	28	24.11	35.53	117	15.8%	64.54 [43.34, 85.74]
Yao Q et al.	38.33	12.33	12	25.57	15.61	13	16.9%	12.76 [1.77, 23.75]
**Total (95% CI)**			**302**			**876**	**100.0%**	**55.87 [27.27, 84.48]**

Heterogeneity: Tau² = 1232.35; Chi² = 75.86, df = 6 (P < 0.00001); I² = 92%
Test for overall effect: Z = 3.83 (P = 0.0001)

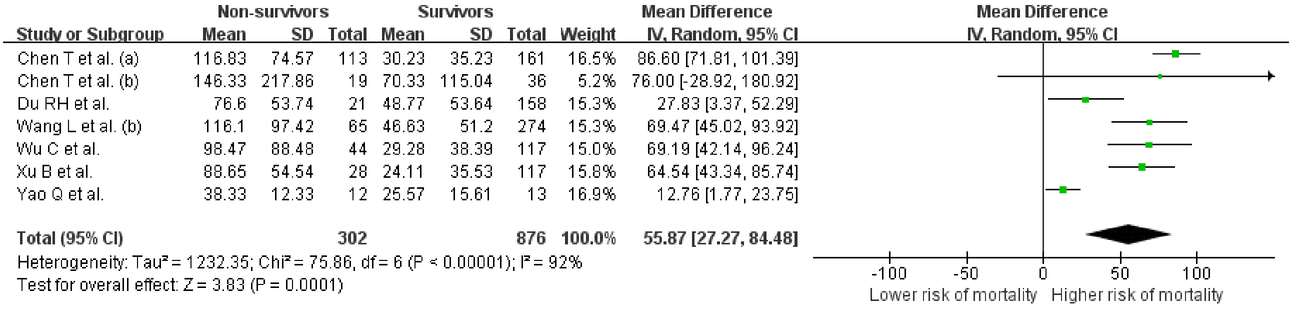

Figure S64. Forest plot of mean difference in C-reactive protein between nonsurvivors and survivors

Study or Subgroup	Non-survivors Mean	SD	Total	Survivors Mean	SD	Total	Weight	Mean Difference IV, Random, 95% CI
Chen T et al. (b)	5.6	12.04	19	6.73	14.45	36	4.9%	-1.13 [-8.31, 6.05]
Du RH et al.	4	8.03	21	0.67	0.67	158	16.2%	3.33 [-0.11, 6.77]
Tang N et al. (a)	9.04	14.67	134	2.14	2.52	315	23.7%	6.90 [4.40, 9.40]
Wang L et al. (b)	7.57	11.9	65	1.22	1.14	274	20.1%	6.35 [3.45, 9.25]
Wu C et al.	5.35	7.52	44	0.59	0.45	117	26.7%	4.76 [2.54, 6.98]
Xu B et al.	7.03	14.49	28	0.62	0.46	117	8.2%	6.41 [1.04, 11.78]
Yao Q et al.	33.41	66.12	12	1.94	1.4	13	0.2%	31.47 [-5.95, 68.89]
**Total (95% CI)**			**323**			**1030**	**100.0%**	**5.26 [3.58, 6.93]**

Heterogeneity: Tau² = 1.46; Chi² = 8.68, df = 6 (P = 0.19); I² = 31%
Test for overall effect: Z = 6.14 (P < 0.00001)

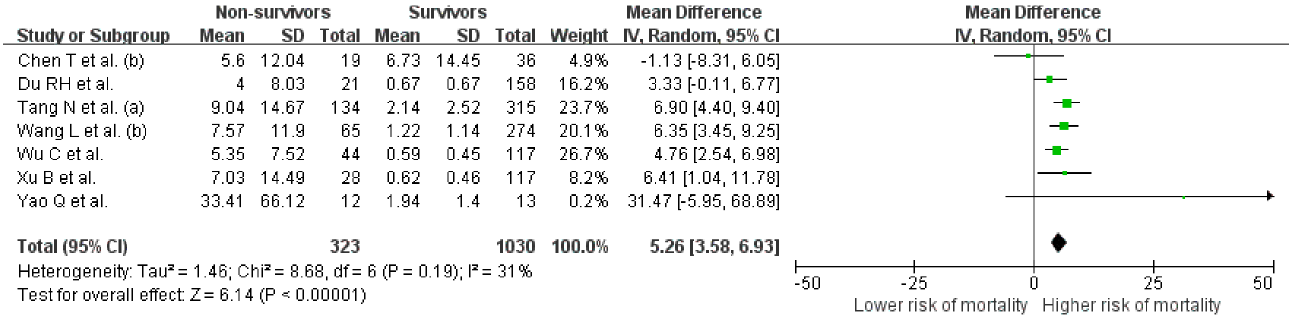

Figure S65. Forest plot of mean difference in D-dimer between nonsurvivors and survivors

Study or Subgroup	Non-survivors Mean	SD	Total	Survivors Mean	SD	Total	Weight	Mean Difference IV, Random, 95% CI
Chen T et al. (a)	0.37	0.38	113	0.05	0.04	161	28.5%	0.32 [0.25, 0.39]
Chen T et al. (b)	3.08	6.41	19	0.36	0.77	36	0.2%	2.72 [-0.17, 5.61]
Wang L et al. (b)	0.49	0.77	65	0.08	0.07	274	18.7%	0.41 [0.22, 0.60]
Xu B et al.	0.25	0.32	28	0.04	0.04	117	24.6%	0.21 [0.09, 0.33]
Yao Q et al.	2.75	4.75	12	0.25	0.25	13	0.2%	2.50 [-0.19, 5.19]
Zhou F et al.	0.23	0.3	54	0.1	0.001	137	27.8%	0.13 [0.05, 0.21]
**Total (95% CI)**			**291**			**738**	**100.0%**	**0.27 [0.14, 0.40]**

Heterogeneity: Tau² = 0.01; Chi² = 21.12, df = 5 (P = 0.0008); I² = 76%
Test for overall effect: Z = 4.06 (P < 0.0001)

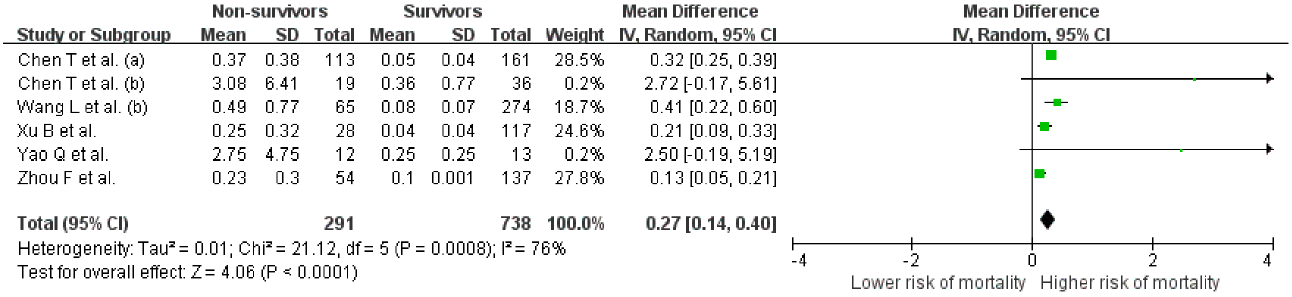

Figure S66. Forest plot of mean difference in procalcitonin between nonsurvivors and survivors

Study or Subgroup	Non-survivors Mean	SD	Total	Survivors Mean	SD	Total	Weight	Mean Difference IV, Random, 95% CI
Chen T et al. (a)	1.5233	0.99184	113	0.53927	0.45362	161	68.3%	0.98 [0.79, 1.18]
Wu C et al.	1.21854	1.10402	44	0.46135	0.35948	117	31.7%	0.76 [0.42, 1.09]
**Total (95% CI)**			**157**			**278**	**100.0%**	**0.91 [0.71, 1.12]**

Heterogeneity: Tau² = 0.01; Chi² = 1.33, df = 1 (P = 0.25); I² = 25%
Test for overall effect: Z = 8.64 (P < 0.00001)

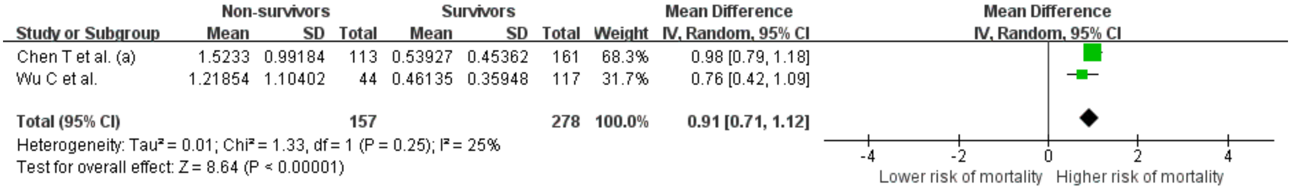

Figure S67. Forest plot of mean difference in ferritin between nonsurvivors and survivors

Study or Subgroup	Non-survivors Mean	SD	Total	Survivors Mean	SD	Total	Weight	Mean Difference IV, Random, 95% CI
Chen T et al. (a)	570.43	213.87	113	266.27	76.45	161	32.4%	304.16 [263.00, 345.32]
Chen T et al. (b)	468.67	498.2	19	383	320.41	36	6.8%	85.67 [-161.59, 332.93]
Wang L et al. (b)	480.67	214.56	65	287	100.61	274	30.2%	193.67 [140.17, 247.17]
Wu C et al.	467.83	166.68	44	262.83	82.19	117	30.6%	205.00 [153.55, 256.45]
**Total (95% CI)**			**241**			**588**	**100.0%**	**225.57 [153.85, 297.29]**

Heterogeneity: Tau² = 3690.21; Chi² = 15.39, df = 3 (P = 0.002); I² = 81%
Test for overall effect: Z = 6.16 (P < 0.00001)

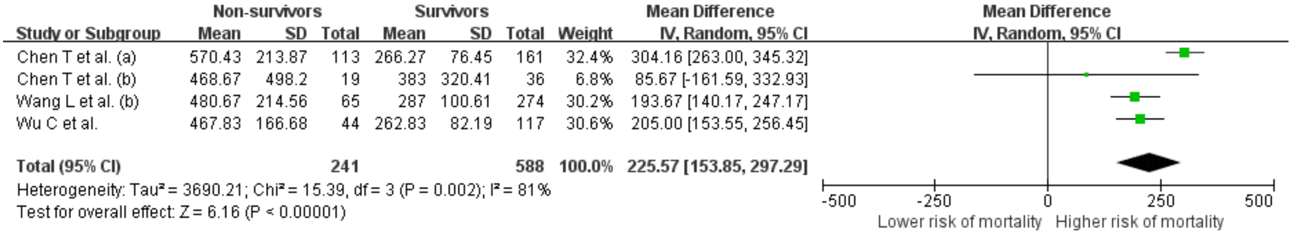

Figure S68. Forest plot of mean difference in lactate dehydrogenase between nonsurvivors and survivors

Study or Subgroup	Non-survivors Mean	SD	Total	Survivors Mean	SD	Total	Weight	Mean Difference IV, Random, 95% CI
Chen T et al. (a)	219.33	210.26	113	91.7	66.95	161	18.8%	127.63 [87.51, 167.75]
Chen T et al. (b)	406.33	751.31	19	125.67	189.16	36	0.9%	280.66 [-62.77, 624.09]
Wang L et al. (b)	118.67	130.4	65	60	16.4	274	21.0%	58.67 [26.91, 90.43]
Xu B et al.	142.33	65.62	28	86.17	67.18	117	22.1%	56.16 [28.98, 83.34]
Yao Q et al.	97.7	52.16	12	106.17	85.96	13	15.1%	-8.47 [-63.74, 46.80]
Zhou F et al.	69.83	100.16	54	27.53	29.67	137	22.1%	42.30 [15.13, 69.47]
**Total (95% CI)**			**291**			**738**	**100.0%**	**59.34 [26.18, 92.50]**

Heterogeneity: Tau² = 1102.48; Chi² = 20.04, df = 5 (P = 0.001); I² = 75%
Test for overall effect: Z = 3.51 (P = 0.0005)

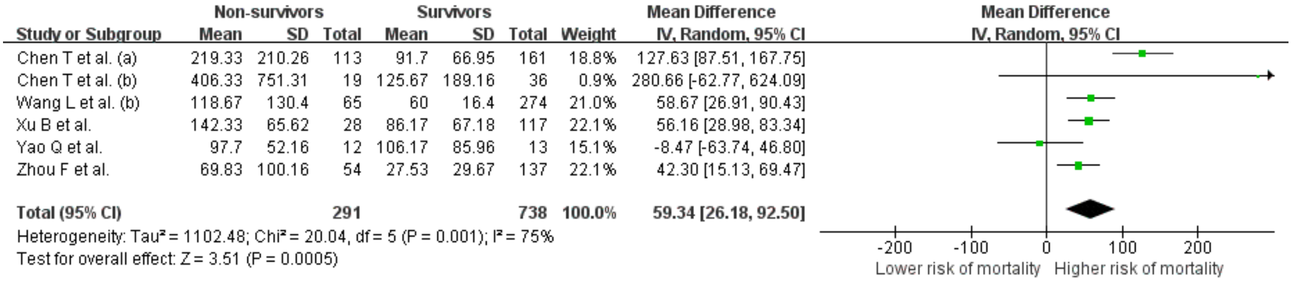

Figure S69. Forest plot of mean difference in creatine kinase between nonsurvivors and survivors

Study or Subgroup	Non-survivors Mean	SD	Total	Survivors Mean	SD	Total	Weight	Mean Difference IV, Random, 95% CI
Chen T et al. (a)	46.33	32.29	113	30.77	19.67	161	51.3%	15.56 [8.88, 22.24]
Du RH et al.	27.17	20.27	21	33.5	28.06	158	48.7%	-6.33 [-16.04, 3.38]
**Total (95% CI)**			**134**			**319**	**100.0%**	**4.91 [-16.53, 26.35]**

Heterogeneity: Tau² = 221.50; Chi² = 13.24, df = 1 (P = 0.0003); I² = 92%
Test for overall effect: Z = 0.45 (P = 0.65)

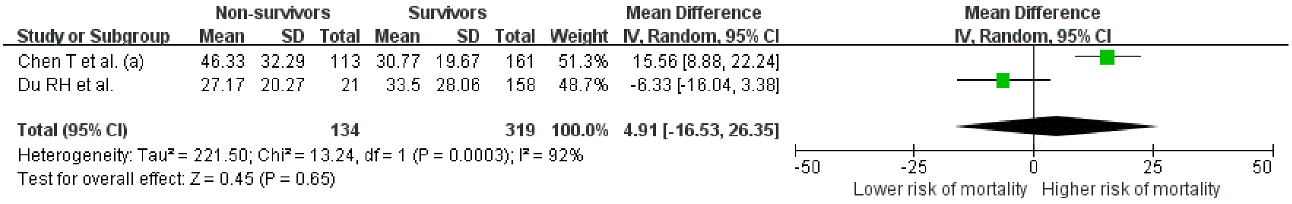

Figure S70. Forest plot of mean difference in γ-glutamyl transpeptidase between nonsurvivors and survivors

Study or Subgroup	Non-survivors Mean	SD	Total	Survivors Mean	SD	Total	Weight	Mean Difference IV, Random, 95% CI
Chen T et al. (a)	40.6	31.76	113	29.6	21.84	161	58.3%	11.00 [4.24, 17.76]
Chen T et al. (b)	50	69.68	19	49.67	64.08	36	1.9%	0.33 [-37.35, 38.01]
Wu C et al.	57.17	25.29	44	50.67	18.24	117	39.9%	6.50 [-1.67, 14.67]
**Total (95% CI)**			**176**			**314**	**100.0%**	**9.01 [3.85, 14.16]**

Heterogeneity: Tau² = 0.00; Chi² = 0.90, df = 2 (P = 0.64); I² = 0%
Test for overall effect: Z = 3.42 (P = 0.0006)

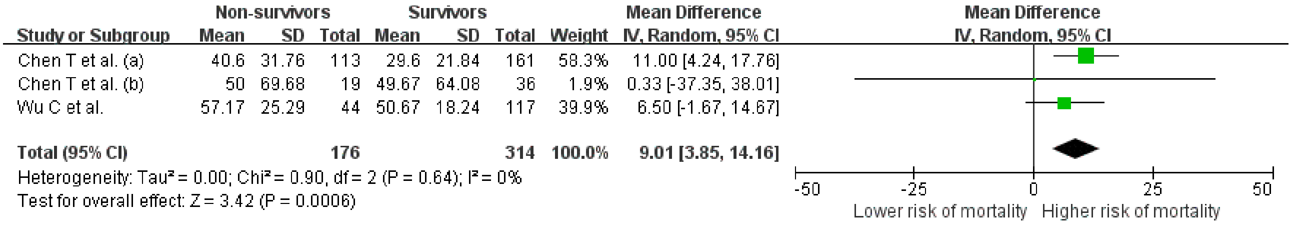

Figure S71. Forest plot of mean difference in ESR between nonsurvivors and survivors

Study or Subgroup	Non-survivors Mean	SD	Total	Survivors Mean	SD	Total	Weight	Mean Difference IV, Random, 95% CI
Wang L et al.	2.85	2.27	65	1.29	0.82	274	40.8%	1.56 [1.00, 2.12]
Wu C et al.	16.67	5.36	44	15.33	5.25	117	32.7%	1.34 [-0.51, 3.19]
Xu B et al.	20.83	10.55	28	13.67	6	117	17.8%	7.16 [3.10, 11.22]
Yao Q et al.	22.57	11.32	12	13.83	4.57	13	8.6%	8.74 [1.87, 15.61]
**Total (95% CI)**			**149**			**521**	**100.0%**	**3.11 [0.84, 5.37]**

Heterogeneity: Tau² = 3.18; Chi² = 11.38, df = 3 (P = 0.010); I² = 74%
Test for overall effect: Z = 2.69 (P = 0.007)

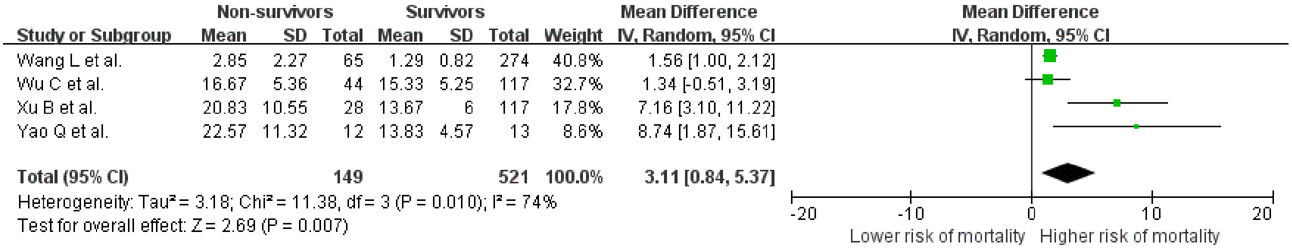

Figure S72. Forest plot of mean difference in creatine kinase-MB between nonsurvivors and survivors

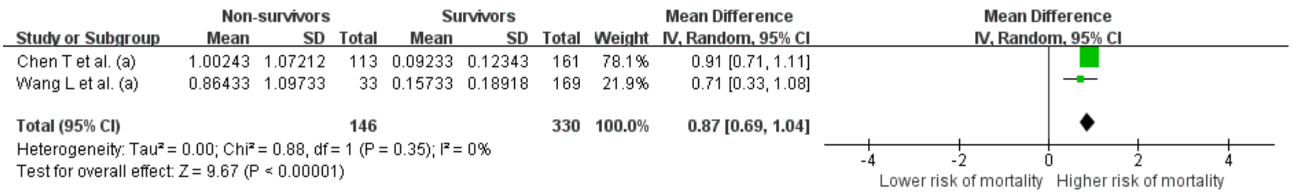

Figure S73. Forest plot of mean difference in NT-proBNP between nonsurvivors and survivors

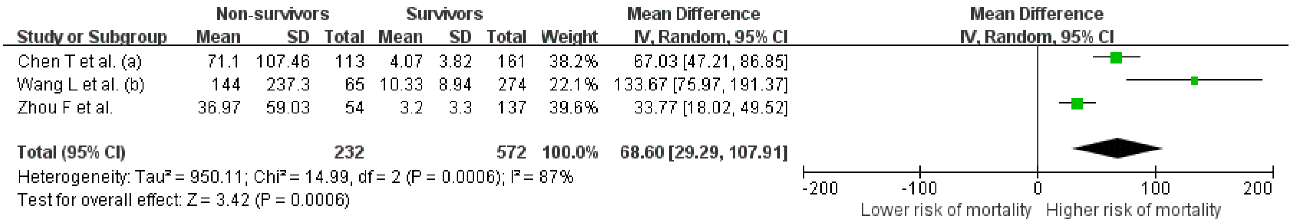

Figure S74. Forest plot of mean difference in hs-cTnI I between nonsurvivors and survivors

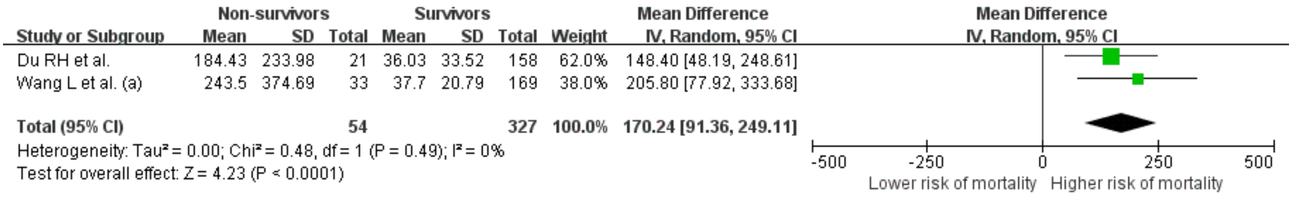

Figure S75. Forest plot of mean difference in myoglobin between nonsurvivors and survivors

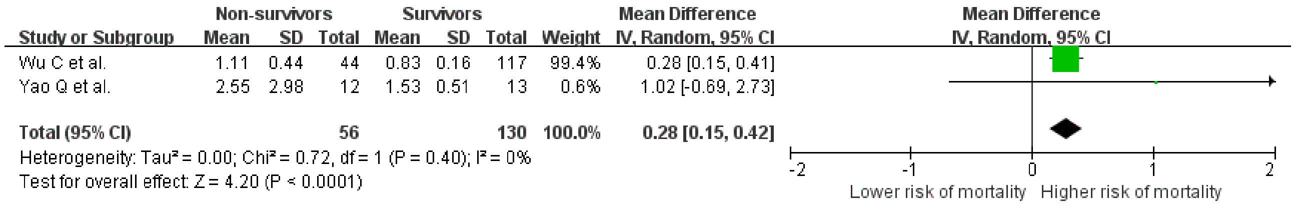

Figure S76. Forest plot of mean difference in cystatin C between nonsurvivors and survivors

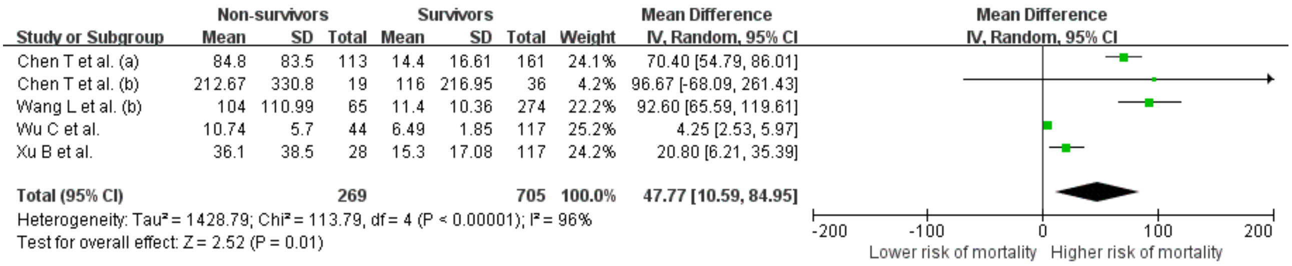

Figure S77. Forest plot of mean difference in interleukin-6between nonsurvivors and survivors

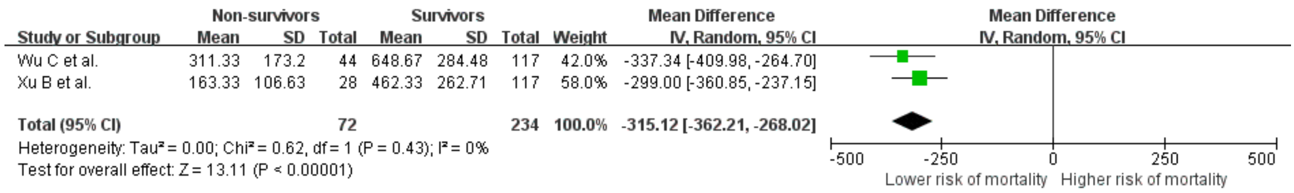

Figure S78. Forest plot of mean difference in CD3+ cell count between nonsurvivors and survivors

Study or Subgroup	Non-survivors Mean	SD	Total	Survivors Mean	SD	Total	Weight	Mean Difference IV, Random, 95% CI
Wang L et al. (b)	193.33	132.68	65	360.67	222.83	274	34.5%	-167.34 [-209.01, -125.67]
Wu C et al.	202.33	141.01	44	408.67	216.93	117	20.3%	-206.34 [-263.62, -149.06]
Xu B et al.	97.42	68.94	28	249.67	131.36	117	45.1%	-152.25 [-187.16, -117.34]
**Total (95% CI)**			**137**			**508**	**100.0%**	**-168.46 [-196.08, -140.84]**

Heterogeneity: Tau² = 122.79; Chi² = 2.50, df = 2 (P = 0.29); I² = 20%
Test for overall effect: Z = 11.95 (P < 0.00001)

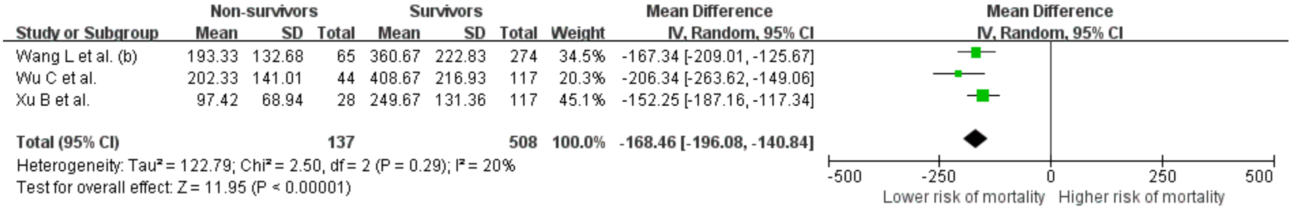

Figure S79. Forest plot of mean difference in CD4+ cell count between nonsurvivors and survivors

Study or Subgroup	Non-survivors Mean	SD	Total	Survivors Mean	SD	Total	Weight	Mean Difference IV, Random, 95% CI
Wang L et al. (b)	91.67	89.46	65	199.67	149.8	274	34.4%	-108.00 [-136.06, -79.94]
Wu C et al.	102.17	58.63	44	241	123.1	117	34.1%	-138.83 [-167.07, -110.59]
Xu B et al.	70.33	56.24	28	171.33	122.35	117	31.5%	-101.00 [-131.42, -70.58]
**Total (95% CI)**			**137**			**508**	**100.0%**	**-116.32 [-139.16, -93.48]**

Heterogeneity: Tau² = 190.18; Chi² = 3.75, df = 2 (P = 0.15); I² = 47%
Test for overall effect: Z = 9.98 (P < 0.00001)

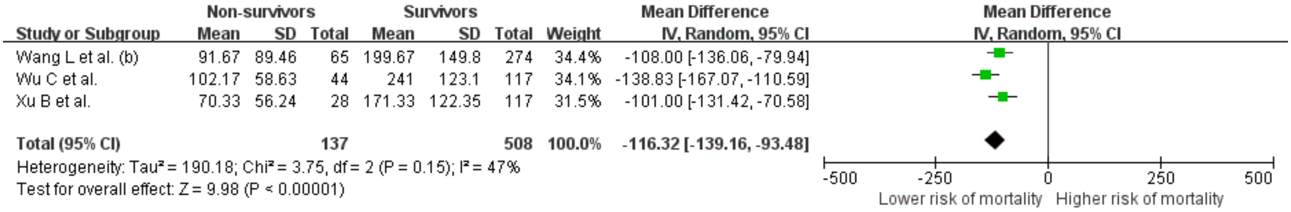

Figure S80. Forest plot of mean difference in CD8+ cell count between nonsurvivors and survivors

Study or Subgroup	Non-survivors Events	Total	Survivors Events	Total	Weight	Risk Ratio M-H, Random, 95% CI
Chen T et al. (a)	56	113	6	161	26.2%	13.30 [5.93, 29.80]
Chen T et al. (b)	4	19	6	36	22.9%	1.26 [0.41, 3.94]
Yao Q et al.	9	12	3	96	22.6%	24.00 [7.52, 76.61]
Zhou F et al.	25	54	15	137	28.3%	4.23 [2.42, 7.38]
**Total (95% CI)**		**198**		**430**	**100.0%**	**6.41 [2.18, 18.82]**
Total events	94		30			

Heterogeneity: Tau² = 0.98; Chi² = 18.35, df = 3 (P = 0.0004); I² = 84%
Test for overall effect: Z = 3.38 (P = 0.0007)

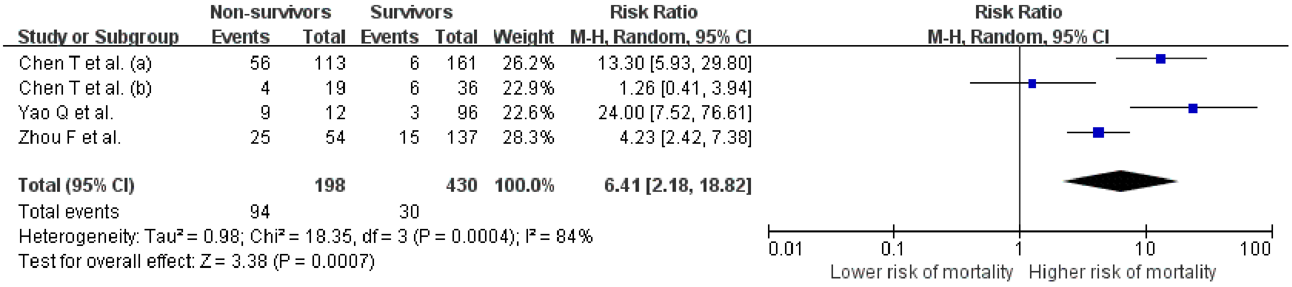

Figure S81. Forest plot of association between increased white blood cell count (≥10 ×10⁹/L) and mortality

Study or Subgroup	Non-survivors Events	Total	Survivors Events	Total	Weight	Risk Ratio M-H, Random, 95% CI
Chen T et al. (a)	87	113	48	161	56.8%	2.58 [2.00, 3.34]
Yao Q et al.	5	12	18	96	6.1%	2.22 [1.01, 4.89]
Zhou F et al.	41	54	36	137	37.2%	2.89 [2.10, 3.97]
**Total (95% CI)**		**179**		**394**	**100.0%**	**2.67 [2.20, 3.24]**
Total events	133		102			

Heterogeneity: Tau² = 0.00; Chi² = 0.51, df = 2 (P = 0.77); I² = 0%
Test for overall effect: Z = 9.91 (P < 0.00001)

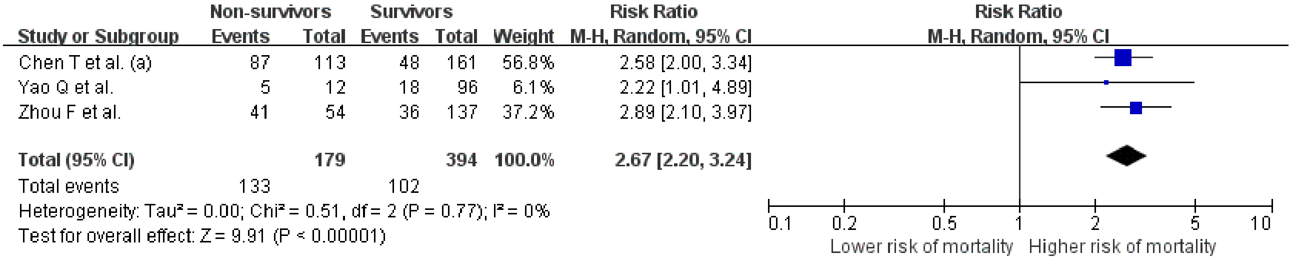

Figure S82. Forest plot of association between decreased lymphocyte count (<0.8 × 10⁹/L) and mortality

Study or Subgroup	Non-survivors Events	Total	Survivors Events	Total	Weight	Risk Ratio M-H, Random, 95% CI
Cao J et al.	11	17	54	85	23.1%	1.02 [0.69, 1.50]
Du RH et al.	19	21	114	158	76.9%	1.25 [1.06, 1.49]
**Total (95% CI)**		**38**		**243**	**100.0%**	**1.20 [0.98, 1.46]**
Total events	30		168			

Heterogeneity: Tau² = 0.01; Chi² = 1.26, df = 1 (P = 0.26); I² = 21%
Test for overall effect: Z = 1.75 (P = 0.08)

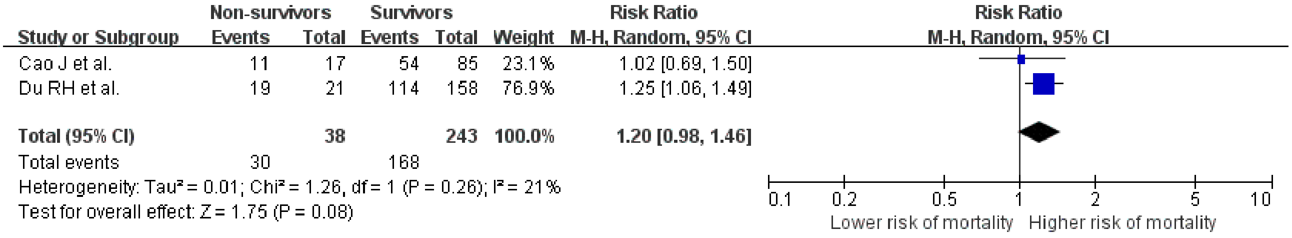

Figure S83. Forest plot of association between decreased lymphocyte count (<1.1 × 10⁹/L) and mortality

Study or Subgroup	Non-survivors Events	Non-survivors Total	Survivors Events	Survivors Total	Weight	Risk Ratio M-H, Random, 95% CI
Chen T et al. (b)	5	19	4	36	38.8%	2.37 [0.72, 7.80]
Yao Q et al.	1	12	9	96	27.0%	0.89 [0.12, 6.42]
Zhou F et al.	11	54	2	137	34.3%	13.95 [3.20, 60.89]
**Total (95% CI)**		**85**		**269**	**100.0%**	**3.34 [0.75, 14.78]**
Total events	17		15			

Heterogeneity: Tau² = 1.12; Chi² = 5.76, df = 2 (P = 0.06); I² = 65%
Test for overall effect: Z = 1.59 (P = 0.11)

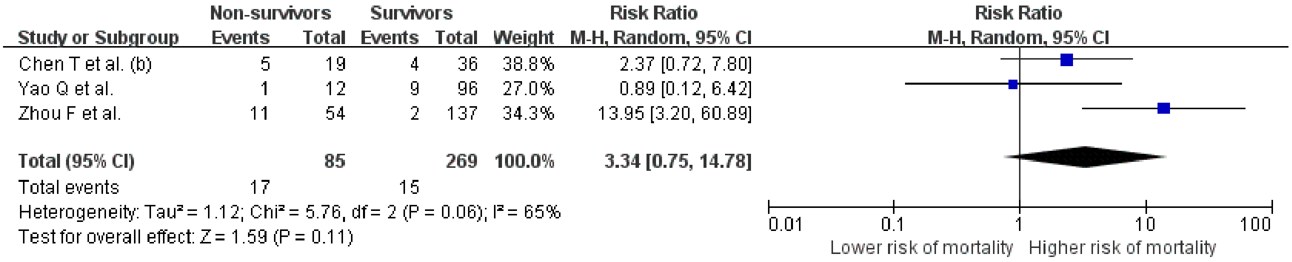

Figure S84. Forest plot of association between decreased platelet count (<100 × 10^9/L) and mortality

Study or Subgroup	Non-survivors Events	Non-survivors Total	Survivors Events	Survivors Total	Weight	Risk Ratio M-H, Random, 95% CI
Liu Y et al. (b)	21	49	47	334	47.1%	3.05 [2.01, 4.63]
Yang X et al. (a)	173	238	133	1238	52.9%	6.77 [5.66, 8.09]
**Total (95% CI)**		**287**		**1572**	**100.0%**	**4.65 [2.13, 10.15]**
Total events	194		180			

Heterogeneity: Tau² = 0.29; Chi² = 11.85, df = 1 (P = 0.0006); I² = 92%
Test for overall effect: Z = 3.85 (P = 0.0001)

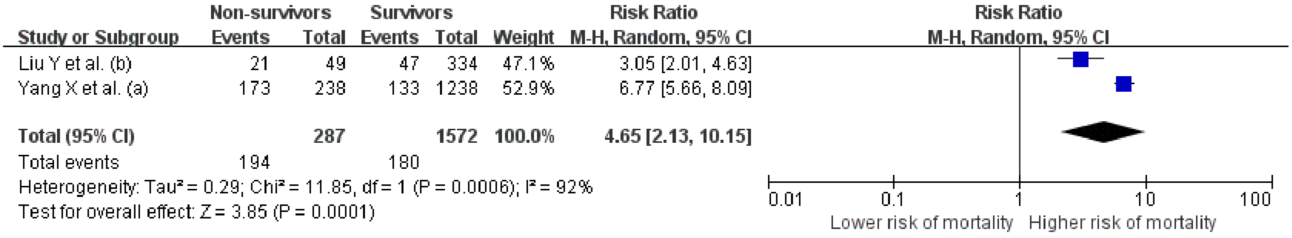

Figure S85. Forest plot of association between decreased platelet count (<125 × 10^9/L) and mortality

Study or Subgroup	Non-survivors Events	Non-survivors Total	Survivors Events	Survivors Total	Weight	Risk Ratio M-H, Random, 95% CI
Cao J et al.	7	17	18	84	15.5%	1.92 [0.95, 3.87]
Chen T et al. (a)	30	113	30	161	38.3%	1.42 [0.91, 2.22]
Zhou F et al.	26	54	33	135	46.2%	1.97 [1.31, 2.96]
**Total (95% CI)**		**184**		**380**	**100.0%**	**1.73 [1.32, 2.28]**
Total events	63		81			

Heterogeneity: Tau² = 0.00; Chi² = 1.24, df = 2 (P = 0.54); I² = 0%
Test for overall effect: Z = 3.91 (P < 0.0001)

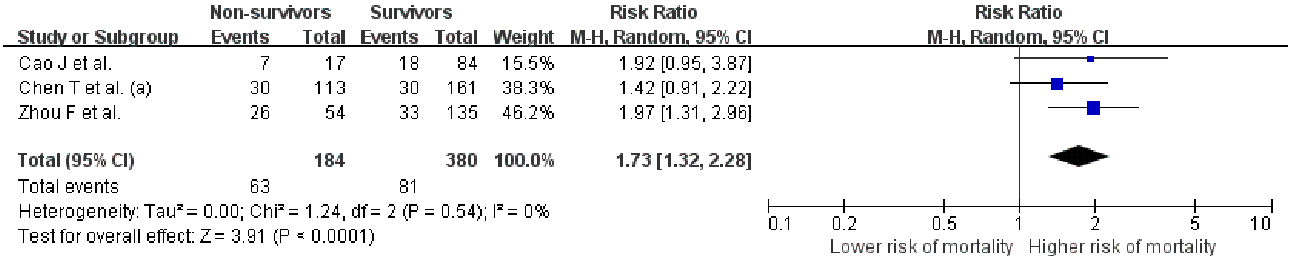

Figure S86. Forest plot of association between increased ALT (>40 U/L) and mortality

Study or Subgroup	Non-survivors Events	Non-survivors Total	Survivors Events	Survivors Total	Weight	Risk Ratio M-H, Random, 95% CI
Chen T et al. (a)	59	113	25	161	34.6%	3.36 [2.25, 5.02]
Chen T et al. (b)	14	19	18	36	34.0%	1.47 [0.97, 2.25]
Du RH et al.	10	21	47	158	31.4%	1.60 [0.96, 2.66]
**Total (95% CI)**		**153**		**355**	**100.0%**	**2.01 [1.13, 3.58]**
Total events	83		90			

Heterogeneity: Tau² = 0.21; Chi² = 10.18, df = 2 (P = 0.006); I² = 80%
Test for overall effect: Z = 2.38 (P = 0.02)

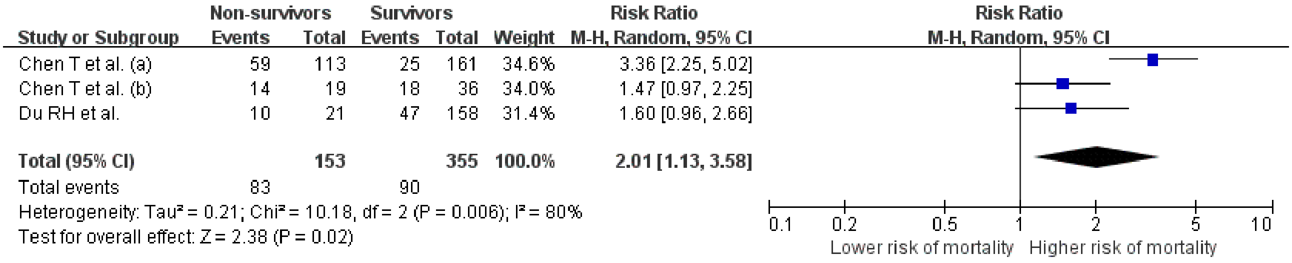

Figure S87. Forest plot of association between increased AST (>40 U/L) and mortality

Study or Subgroup	Non-survivors Events	Non-survivors Total	Survivors Events	Survivors Total	Weight	Risk Ratio M-H, Random, 95% CI
Yao Q et al.	1	12	3	96	29.0%	2.67 [0.30, 23.64]
Zhou F et al.	5	54	3	132	71.0%	4.07 [1.01, 16.45]
**Total (95% CI)**		**66**		**228**	**100.0%**	**3.60 [1.11, 11.68]**
Total events	6		6			

Heterogeneity: Tau² = 0.00; Chi² = 0.10, df = 1 (P = 0.75); I² = 0%
Test for overall effect: Z = 2.14 (P = 0.03)

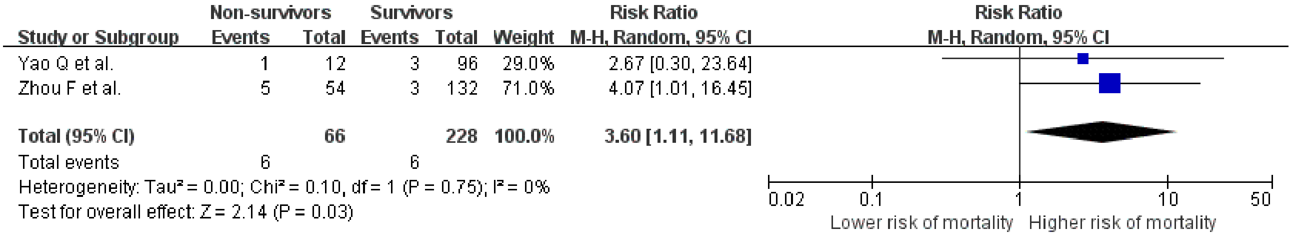

Figure S88. Forest plot of association between increased creatinine (>133 μmol/L) and mortality

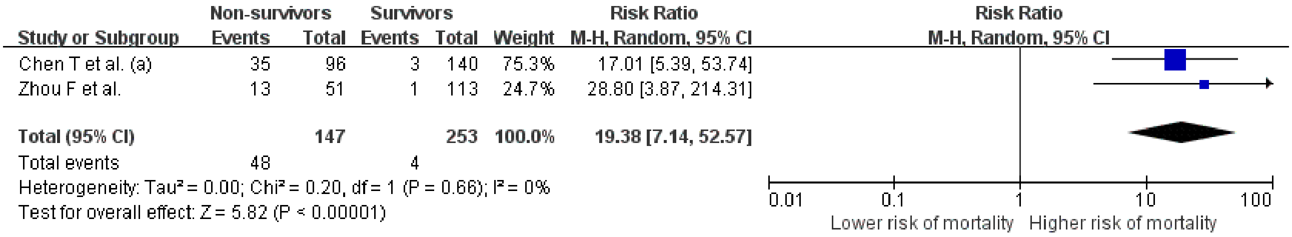

Figure S89. Forest plot of association between increased procalcitonin (≥0.5 ng/mL) and mortality

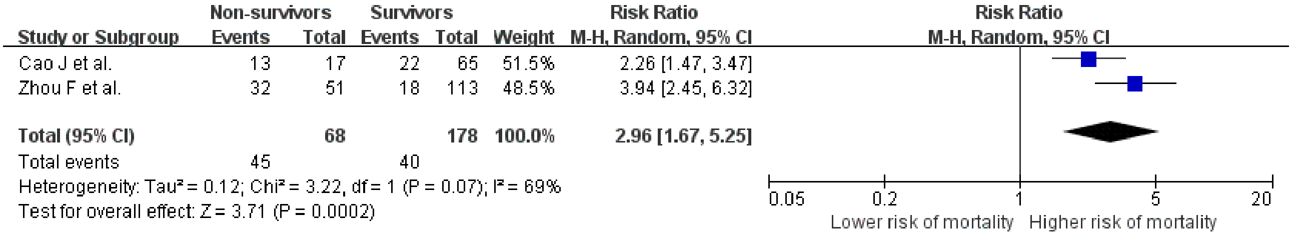

Figure S90. Forest plot of association between increased procalcitonin (≥0.1 ng/mL) and mortality

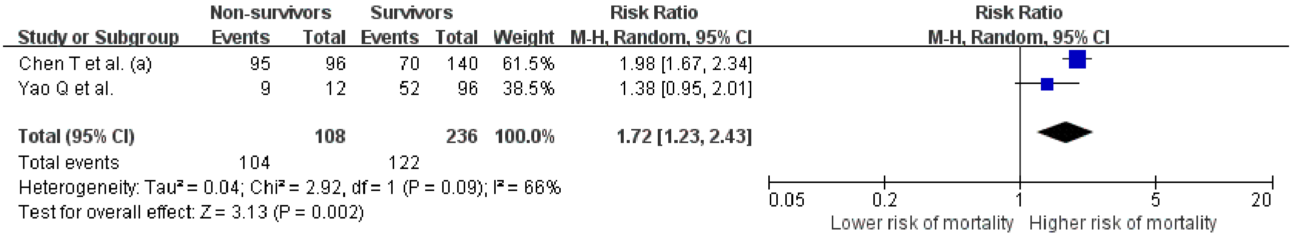

Figure S91. Forest plot of association between increased procalcitonin (≥0.05 ng/mL) and mortality

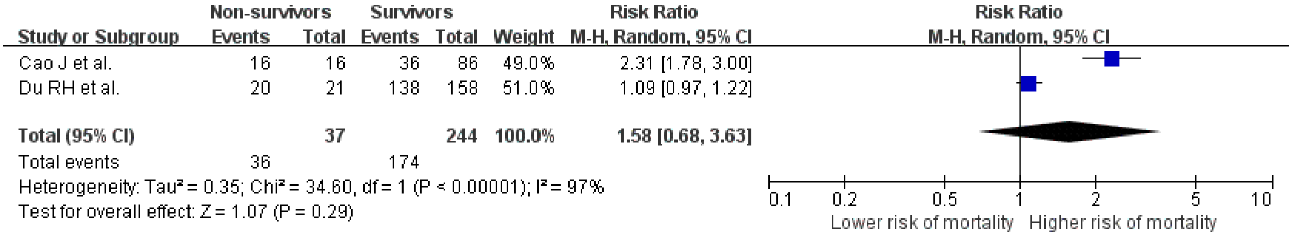

Figure S92. Forest plot of association between increased C-reactive protein (≥10 mg/L) and mortality

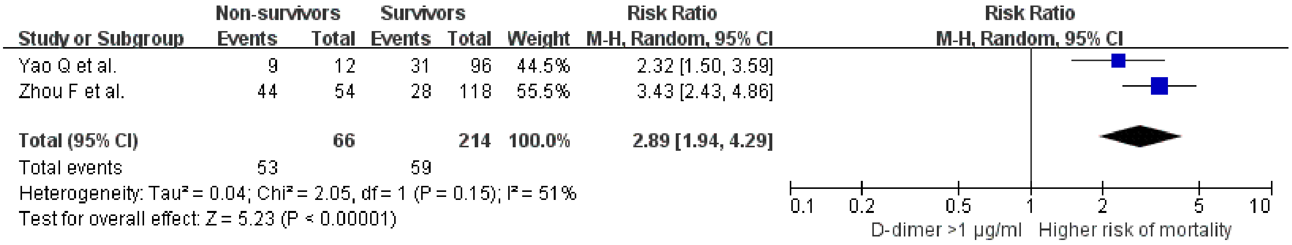

Figure S93. Forest plot of association between increased D-dimer (>1.0 µg/mL) and mortality

Study or Subgroup	Non-survivors Events	Non-survivors Total	Survivors Events	Survivors Total	Weight	Risk Ratio M-H, Random, 95% CI
Chen T et al. (a)	30	113	5	161	68.1%	8.55 [3.42, 21.36]
Liu Y et al. (a)	4	33	4	211	31.9%	6.39 [1.68, 24.33]
**Total (95% CI)**		**146**		**372**	**100.0%**	**7.79 [3.66, 16.58]**
Total events	34		9			

Heterogeneity: Tau² = 0.00; Chi² = 0.13, df = 1 (P = 0.71); I² = 0%
Test for overall effect: Z = 5.33 (P < 0.00001)

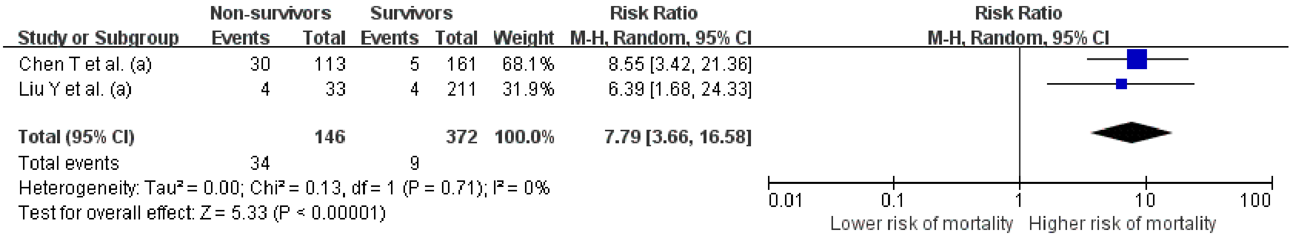

Figure S94. Forest plot of association between respiratory rate ≥30 breaths per min and mortality

Study or Subgroup	Non-survivors Events	Non-survivors Total	Survivors Events	Survivors Total	Weight	Risk Ratio M-H, Random, 95% CI
Chen T et al. (a)	66	113	22	161	51.9%	4.27 [2.81, 6.49]
Zhou F et al.	34	54	22	137	48.1%	3.92 [2.54, 6.05]
**Total (95% CI)**		**167**		**298**	**100.0%**	**4.10 [3.03, 5.54]**
Total events	100		44			

Heterogeneity: Tau² = 0.00; Chi² = 0.08, df = 1 (P = 0.78); I² = 0%
Test for overall effect: Z = 9.18 (P < 0.00001)

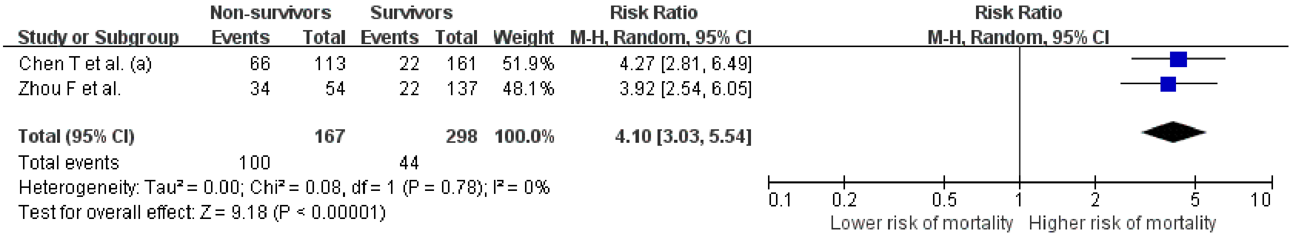

Figure S95. Forest plot of association between respiratory rate ≥24 breaths per min and mortality

Study or Subgroup	Non-survivors Events	Non-survivors Total	Survivors Events	Survivors Total	Weight	Risk Ratio M-H, Random, 95% CI
Cao J et al.	14	17	58	85	1.9%	1.21 [0.93, 1.57]
Chen T et al. (a)	113	113	152	161	81.6%	1.06 [1.02, 1.10]
Yao Q et al.	12	12	86	96	8.0%	1.08 [0.95, 1.23]
Zhang J et al.	20	25	530	638	3.3%	0.96 [0.79, 1.18]
Zhou F et al.	45	54	98	137	5.2%	1.16 [0.99, 1.37]
**Total (95% CI)**		**221**		**1117**	**100.0%**	**1.06 [1.03, 1.10]**
Total events	204		924			

Heterogeneity: Tau² = 0.00; Chi² = 3.75, df = 4 (P = 0.44); I² = 0%
Test for overall effect: Z = 3.34 (P = 0.0008)

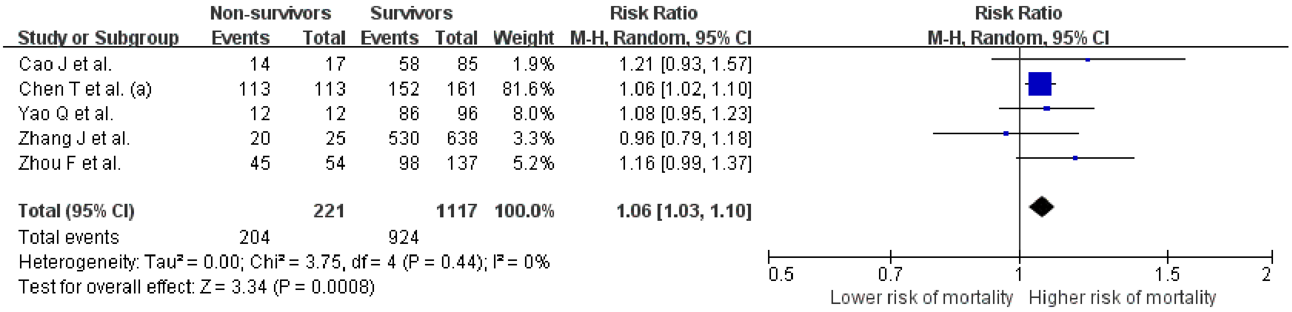

Figure S96. Forest plot of association between bilateral pneumonia and mortality

Study or Subgroup	Non-survivors Events	Non-survivors Total	Survivors Events	Survivors Total	Weight	Risk Ratio M-H, Random, 95% CI
Wu C et al.	14	44	63	157	57.5%	0.79 [0.49, 1.27]
Yao Q et al.	3	12	7	96	42.5%	3.43 [1.02, 11.52]
**Total (95% CI)**		**56**		**253**	**100.0%**	**1.48 [0.36, 6.12]**
Total events	17		70			

Heterogeneity: Tau² = 0.86; Chi² = 4.88, df = 1 (P = 0.03); I² = 80%
Test for overall effect: Z = 0.54 (P = 0.59)

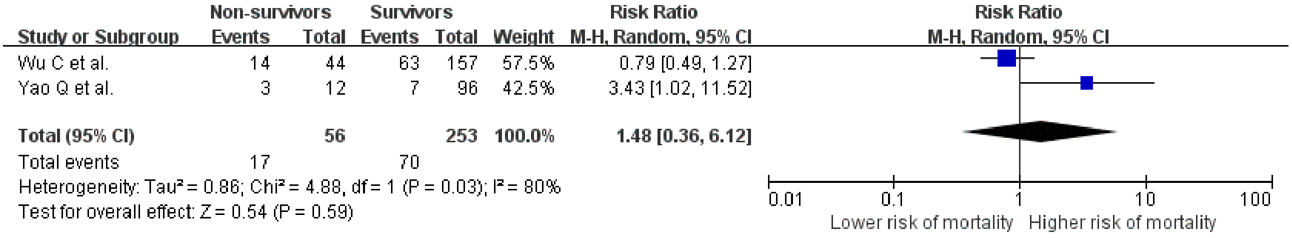

Figure S97. Forest plot of association between highest temperature ≥39 ℃ and mortality

Study or Subgroup	Non-survivors Mean	Non-survivors SD	Non-survivors Total	Survivors Mean	Survivors SD	Survivors Total	Weight	Mean Difference IV, Random, 95% CI
Chen T et al. (a)	24.67	7.51	113	20.33	0.75	161	26.4%	4.34 [2.95, 5.73]
Du RH et al.	24.83	11.53	21	20.33	0.75	158	9.6%	4.50 [-0.43, 9.43]
Hu H et al.	23.21	5.38	19	20.27	3.27	86	19.7%	2.94 [0.42, 5.46]
Wang L et al. (b)	23	9.1	65	19.33	1.49	274	21.4%	3.67 [1.45, 5.89]
Yao Q et al.	20.33	2.52	12	20.33	2.49	13	22.9%	0.00 [-1.97, 1.97]
**Total (95% CI)**			**230**			**692**	**100.0%**	**2.94 [1.10, 4.79]**

Heterogeneity: Tau² = 2.86; Chi² = 13.19, df = 4 (P = 0.01); I² = 70%
Test for overall effect: Z = 3.12 (P = 0.002)

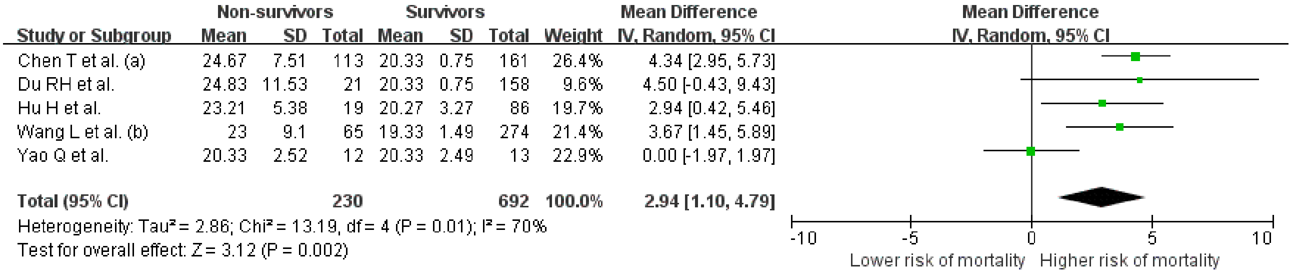

Figure S98. Forest plot of mean difference in respiratory rate between nonsurvivors and survivors

Study or Subgroup	Non-survivors Mean	Non-survivors SD	Non-survivors Total	Survivors Mean	Survivors SD	Survivors Total	Weight	Mean Difference IV, Random, 95% CI
Chen T et al. (a)	98	21.78	113	91.33	18.7	161	27.2%	6.67 [1.72, 11.62]
Du RH et al.	93.83	25.04	21	87.6	15.94	158	6.3%	6.23 [-4.76, 17.22]
Hu H et al.	87.47	13.56	19	84.23	17.48	86	14.3%	3.24 [-3.89, 10.37]
Wang L et al. (b)	87.67	17.44	65	82.67	10.43	274	32.8%	5.00 [0.58, 9.42]
Yang X et al. (b)	89	15	32	89	20	20	7.3%	0.00 [-10.19, 10.19]
Yao Q et al.	81	12.58	12	84.33	5.81	13	12.1%	-3.33 [-11.12, 4.46]
**Total (95% CI)**			**262**			**712**	**100.0%**	**3.91 [1.09, 6.72]**

Heterogeneity: Tau² = 1.20; Chi² = 5.51, df = 5 (P = 0.36); I² = 9%
Test for overall effect: Z = 2.72 (P = 0.006)

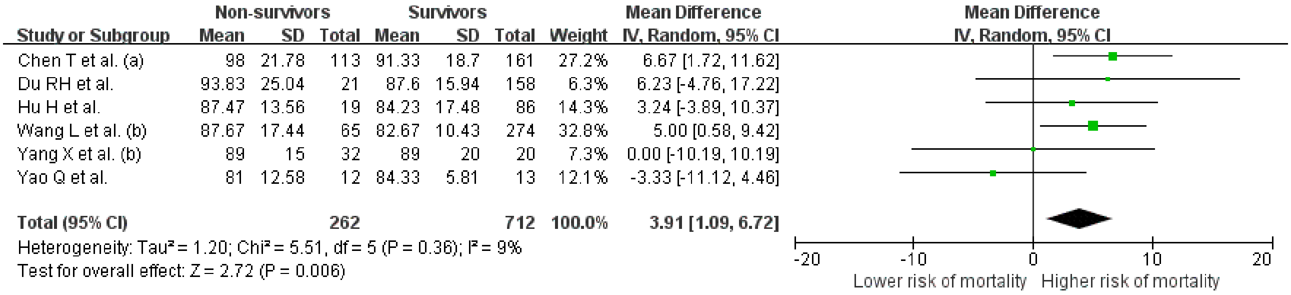

Figure S99. Forest plot of mean difference in heart rate between nonsurvivors and survivors

Study or Subgroup	Non-survivors Mean	Non-survivors SD	Non-survivors Total	Survivors Mean	Survivors SD	Survivors Total	Weight	Mean Difference IV, Random, 95% CI
Chen T et al. (a)	61.07	25.66	35	124.97	56.42	32	29.4%	-63.90 [-85.22, -42.58]
Du RH et al.	58.67	17.49	21	75.17	24.69	158	36.5%	-16.50 [-24.91, -8.09]
Peng YD et al.	78.67	28.29	17	90	12.04	95	34.0%	-11.33 [-24.99, 2.33]
**Total (95% CI)**			**73**			**285**	**100.0%**	**-28.68 [-52.71, -4.65]**

Heterogeneity: Tau² = 392.93; Chi² = 18.50, df = 2 (P < 0.0001); I² = 89%
Test for overall effect: Z = 2.34 (P = 0.02)

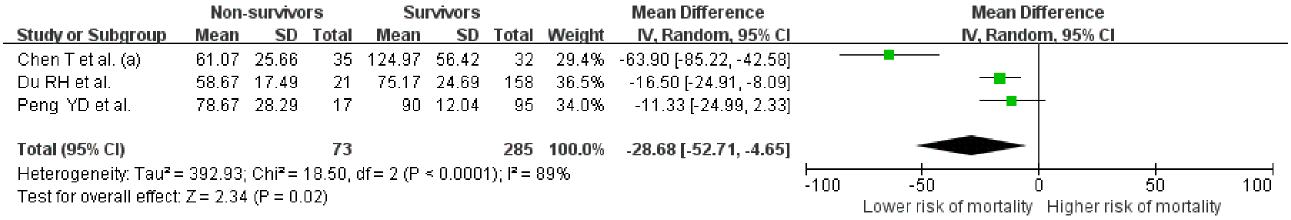

Figure S100. Forest plot of mean difference in PaO_2 between nonsurvivors and survivors

Study or Subgroup	Non-survivors Mean	Non-survivors SD	Non-survivors Total	Survivors Mean	Survivors SD	Survivors Total	Weight	Mean Difference IV, Random, 95% CI
Chen T et al. (a)	31.93	5.49	35	37.93	4.89	32	59.3%	-6.00 [-8.49, -3.51]
Du RH et al.	34.67	9.54	21	37.33	5.24	158	21.1%	-2.66 [-6.82, 1.50]
Peng YD et al.	37	8.89	17	41.67	4.52	95	19.6%	-4.67 [-8.99, -0.35]
**Total (95% CI)**			**73**			**285**	**100.0%**	**-5.03 [-6.95, -3.12]**

Heterogeneity: Tau² = 0.00; Chi² = 1.86, df = 2 (P = 0.40); I² = 0%
Test for overall effect: Z = 5.16 (P < 0.00001)

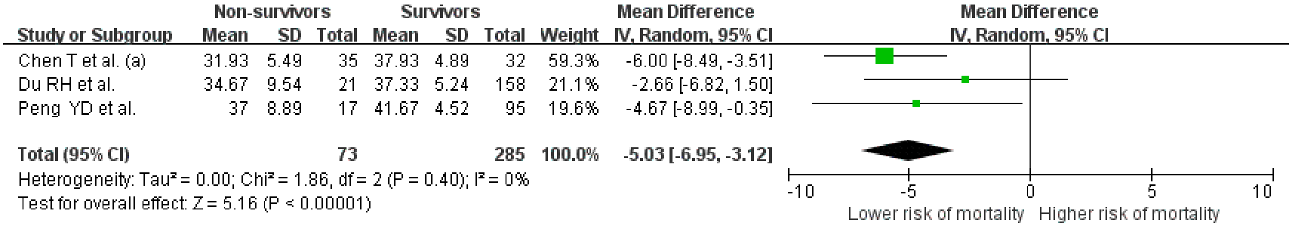

Figure S101. Forest plot of mean difference in $PaCO_2$ between nonsurvivors and survivors

Study or Subgroup	Non-survivors Mean	Non-survivors SD	Non-survivors Total	Survivors Mean	Survivors SD	Survivors Total	Weight	Mean Difference IV, Random, 95% CI
Chen T et al. (a)	84.67	14.27	113	96.77	2.02	161	34.3%	-12.10 [-14.75, -9.45]
Hu H et al.	86.53	12.46	19	96.17	3.1	86	30.6%	-9.64 [-15.28, -4.00]
Peng YD et al.	95.33	3.23	17	96.33	2.26	95	35.1%	-1.00 [-2.60, 0.60]
**Total (95% CI)**			**149**			**342**	**100.0%**	**-7.45 [-15.86, 0.96]**

Heterogeneity: Tau² = 51.83; Chi² = 53.05, df = 2 (P < 0.00001); I² = 96%
Test for overall effect: Z = 1.74 (P = 0.08)

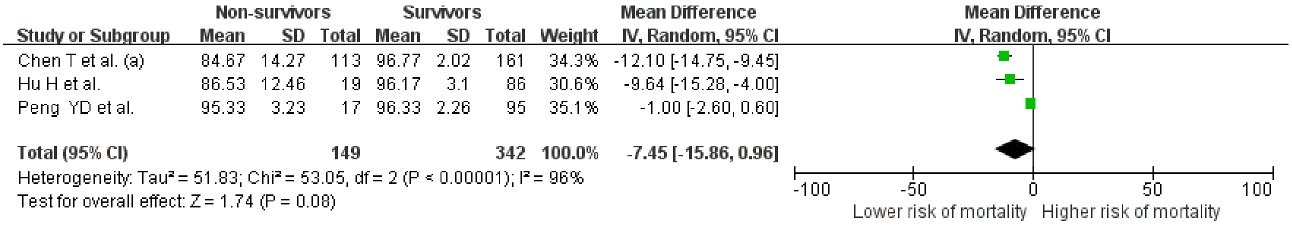

Figure S102. Forest plot of mean difference in SpO2 between nonsurvivors and survivors

Study or Subgroup	Non-survivors Mean	Non-survivors SD	Non-survivors Total	Survivors Mean	Survivors SD	Survivors Total	Weight	Mean Difference IV, Random, 95% CI
Chen T et al. (a)	117.13	71.5	35	329.67	151.33	32	25.5%	-212.54 [-270.07, -155.01]
Du RH et al.	185.5	64.8	21	261.5	108.2	158	28.3%	-76.00 [-108.45, -43.55]
Peng YD et al.	215.67	253.02	17	429.33	25.59	95	17.0%	-213.66 [-334.05, -93.27]
Yang X et al. (b)	62.8	17.15	32	99.03	47.95	20	29.2%	-36.23 [-58.07, -14.39]
**Total (95% CI)**			**105**			**305**	**100.0%**	**-122.62 [-198.25, -46.98]**

Heterogeneity: Tau² = 4981.89; Chi² = 38.20, df = 3 (P < 0.00001); I² = 92%
Test for overall effect: Z = 3.18 (P = 0.001)

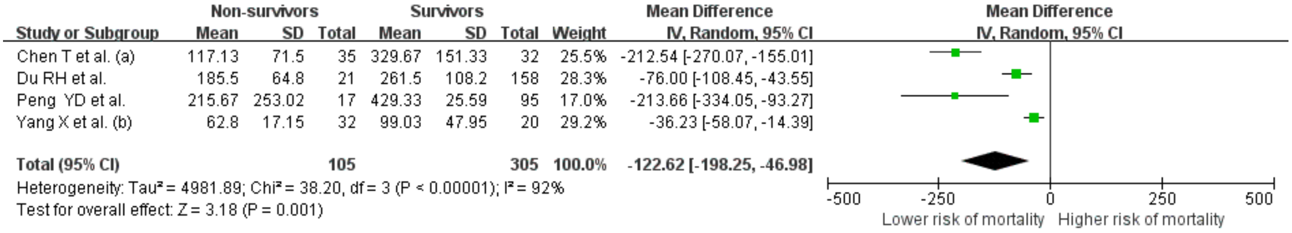

Figure S103. Forest plot of mean difference in PaO_2/FiO_2 between nonsurvivors and survivors

Study or Subgroup	Non-survivors Mean	SD	Total	Survivors Mean	SD	Total	Weight	Mean Difference IV, Random, 95% CI
Yang X et al. (b)	18	3.1	32	14.33	3.99	20	90.7%	3.67 [1.62, 5.72]
Yao Q et al.	11.67	10.9	12	6	3.32	13	9.3%	5.67 [-0.76, 12.10]
**Total (95% CI)**			**44**			**33**	**100.0%**	**3.86 [1.90, 5.81]**

Heterogeneity: Tau^2 = 0.00; Chi^2 = 0.34, df = 1 (P = 0.56); I^2 = 0%
Test for overall effect: Z = 3.87 (P = 0.0001)

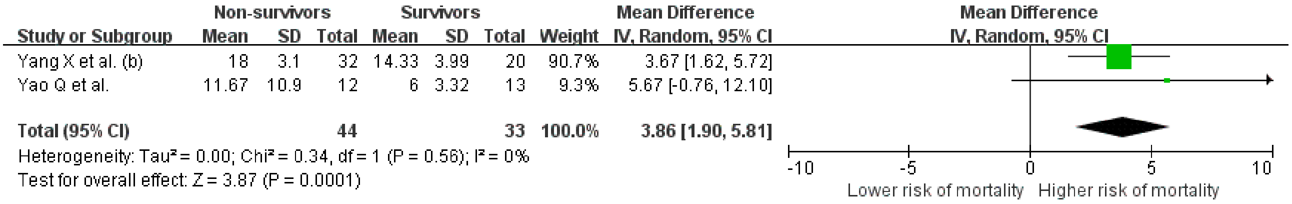

Figure S104. Forest plot of mean difference in APACHE II score between nonsurvivors and survivors

Study or Subgroup	Non-survivors Mean	SD	Total	Survivors Mean	SD	Total	Weight	Mean Difference IV, Random, 95% CI
Yao Q et al.	4.33	5.03	12	2	1.66	13	2.0%	2.33 [-0.66, 5.32]
Zhou F et al.	4.83	1.52	54	1.33	0.75	137	98.0%	3.50 [3.08, 3.92]
**Total (95% CI)**			**66**			**150**	**100.0%**	**3.48 [3.06, 3.90]**

Heterogeneity: Tau^2 = 0.00; Chi^2 = 0.58, df = 1 (P = 0.45); I^2 = 0%
Test for overall effect: Z = 16.22 (P < 0.00001)

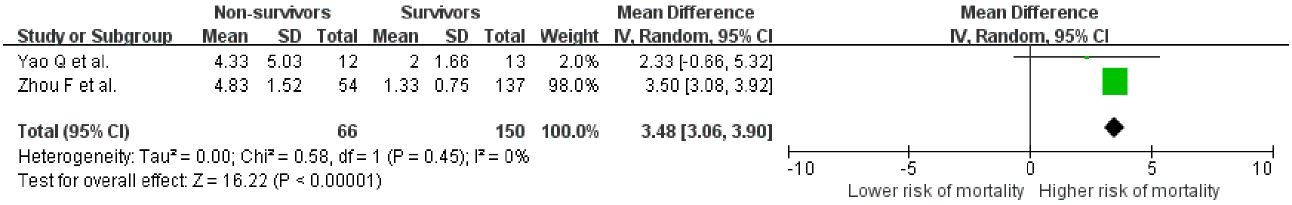

Figure S105. Forest plot of mean difference in SOFA score between nonsurvivors and survivors

Study or Subgroup	Non-survivors Mean	SD	Total	Survivors Mean	SD	Total	Weight	Mean Difference IV, Random, 95% CI
Cao J et al.	5.67	4.04	17	5.33	3.02	85	10.5%	0.34 [-1.68, 2.36]
Chen T et al. (a)	10	4.51	113	9	4.49	161	36.7%	1.00 [-0.08, 2.08]
Hu H et al.	8.37	5.78	19	10.24	7.51	86	4.6%	-1.87 [-4.92, 1.18]
Wang L et al. (b)	10.33	5.31	65	10	4.47	274	22.1%	0.33 [-1.07, 1.73]
Yao Q et al.	7.33	2.52	12	6	3.32	13	8.1%	1.33 [-0.97, 3.63]
Zhou F et al.	11.33	5.33	54	10.67	3.75	137	17.8%	0.66 [-0.89, 2.21]
**Total (95% CI)**			**280**			**756**	**100.0%**	**0.62 [-0.04, 1.27]**

Heterogeneity: Tau^2 = 0.00; Chi^2 = 3.65, df = 5 (P = 0.60); I^2 = 0%
Test for overall effect: Z = 1.84 (P = 0.07)

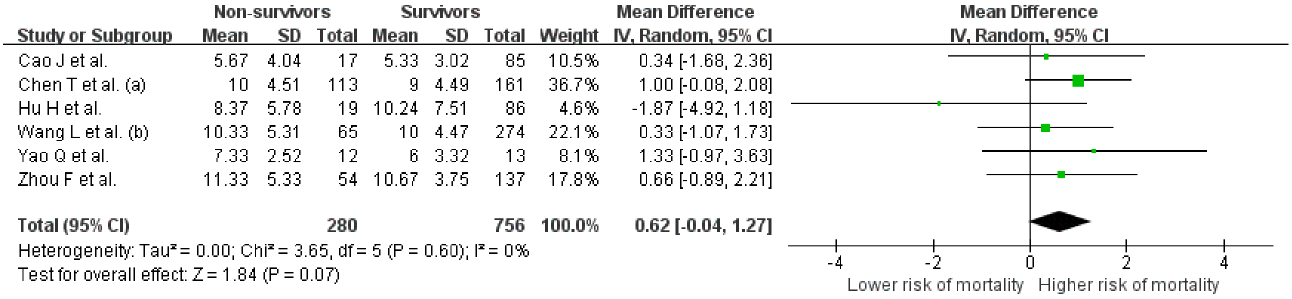

Figure S106. Forest plot of mean difference in Time from illness onset to hospital admission between nonsurvivors and survivors
